# Aid, debt, International Monetary Fund conditionalities and domestic health financing in low- and middle-income countries

**DOI:** 10.1093/heapol/czag070

**Published:** 2026-05-21

**Authors:** Frederik Federspiel, Josephine Borghi

**Affiliations:** Department of Global Health and Development, London School of Hygiene and Tropical Medicine, 15-17 Tavistock Place, London WC1H 9SH, United Kingdom; Department of Global Health and Development, London School of Hygiene and Tropical Medicine, 15-17 Tavistock Place, London WC1H 9SH, United Kingdom; Health, Ageing and Health Systems Research Group, International Institute for Applied Systems Analysis, Schlossplatz 1, Laxenburg A-2361, Austria

**Keywords:** aid, debt, conditionality, IMF, health financing, LMICs

## Abstract

Across low- and middle-income countries (LMICs), public external debt burdens as well as the number of International Monetary Fund (IMF) loan conditionalities have grown over time. These externally derived macro-fiscal factors, along with official development assistance (ODA), may influence the fiscal space for health and policy decisions that co-determine to what extent countries finance their health systems with domestic government funds (GHE-S), and to what extent they rely on household out-of-pocket payments (OOP). The levels and balance of these sources have great implications for health service access and health outcomes, particularly among poorer population groups. However, we did not identify studies that have jointly examined how these key external factors are associated with GHE-S and OOP, nor compared their correlation sizes. This is key for understanding which might be the most effective policy levers for pursuing universal health coverage (UHC). We performed a panel data study of 105 LMICs from 2005 to 2019, investigating associations between GHE-S and OOP, and a set of ODA-, public external debt- and IMF programme, and conditionality variables. We used the generalized method of moments estimator and performed a range of robustness checks. Increases in ODA via the recipient country public sector were associated with modest reductions in both OOP and GHE-S, measured per GDP. Increases in public external debt servicing per GDP were associated with slight relative increases in OOP and slight relative decreases in GHE-S per CHE. We found no relationship between IMF programme participation or conditionalities and GHE-S or OOP. Our findings support less donor concern of aid fungibility in the health sector, while adding that both on- and off-budget ODA for health also appear to modestly subsidize OOP. Our findings for debt indicated a small shift in the burden of payment from the government onto the user from increasing public external debt servicing. This provides some added support to calls for debt resolution among more heavily indebted LMICs to avoid the negative health service access implications from OOP.

Key messages

- Debt burdens are growing across a number of LMICs, threatening to crowd out government health spending with resulting reliance on user payments for health services. Debt servicing occurs in a context of additional external macroeconomic influence that includes both development assistance flows and IMF conditionalities, which can also influence health financing.
- We provide the first joint analysis of the associations between these three factors and domestic government health spending and user fees in LMICs and subject our results to extensive sensitivity analysis.
- We find a small shift in the burden of payment from the government onto the user from increasing public external debt servicing, adding modest empirical support to the argument for debt resolution among more heavily indebted LMICs from a Universal Health Coverage perspective.
- We also confirm previous findings of fungibility of on-budget ODA for health, though at a modest level, and add to the literature that both on- and off-budget ODA for health also appear to mildly subsidize OOP. These findings should encourage development partners: While they do to a limited extent act as subsidizing agents for recipient governments, it also appears they help subsidize health service costs paid with the most inequitable source of financing.

## Introduction

Each year, international development agencies and international financial institutions [IFIs, i.e. the International Monetary Fund (IMF), the World Bank (WB), and others], send billions of dollars of development assistance and foreign credit to recipient countries, broadly aiming to reduce poverty and achieve socioeconomic development, and in the case of the IMF, to achieve macroeconomic stability including stabilizing the balance of payments, improve credit ratings and ensure a country’s ability to repay its debts (IMF 2024c). Part of this external financial assistance is provided as loans that require repayment, and come with specific policy conditionalities, that often extend beyond the basic terms of repayment. Specifically, loans obtained from the IMF come with loan conditionalities, traditionally involving measures of austerity, fiscal consolidation, and decentralization, with emphasis on private sector-led growth. Before the recent cuts to official development assistance (ODA) from several countries (Sheldrick 2025), ODA, external debt, and IMF conditionalities have all been on the rise during the last decade.

**Figure 1:**
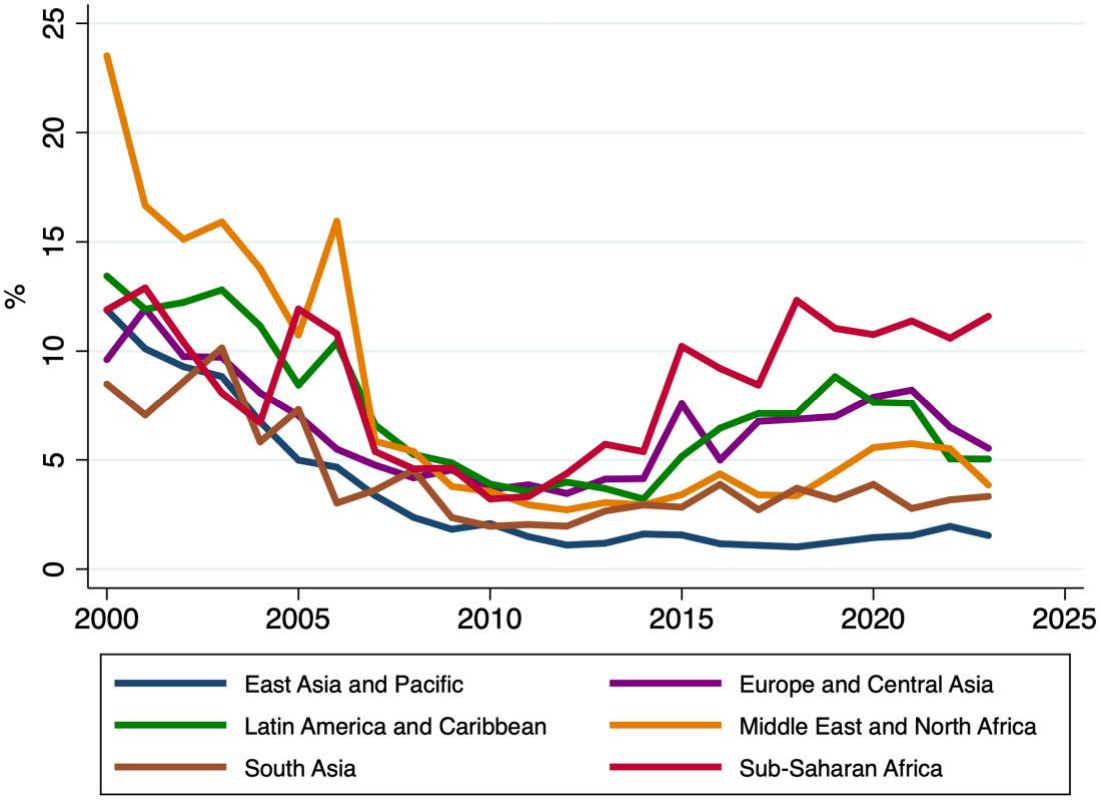
Public and publicly guaranteed (PPG) external debt service out of total government expenditure in 85 LMICs separated by region, 2000–23 (%) (IMF 2024b; World Bank 2024a). Data from 85 countries with complete data.

ODA, including for health, expanded substantially in real terms from 2002 to 2023 (OECD 2024). So did public external debt burdens of low- and middle-income countries (LMICs) after 2010 (Fig. 1) (IMF 2024b, World Bank 2024a). This has led the United Nations and the WB, among a number of civil society organizations and academics, to express concern of unsustainable debt levels and how this may affect countries’ ability to finance health under this growing fiscal pressure (Jubilee Debt Campaign 2020, Development Finance International 2023, Fan and Gupta 2023, United Nations Global Crisis Response Group 2023, World Bank 2023, Amnesty International 2024, OXFAM 2024, The Lancet 2024). In particular, sub-Saharan African (SSA) governments have the highest external debt burdens relative to their budgets (Fig. 1) (IMF 2024b, World Bank 2024a), and this is where health financing is already scarcest (WHO 2024). Contrary to common beliefs, after a decline in the 2000s, the mean number of structural conditions in IMF programmes increased since 2008, with some countries having experienced more than 1000 total IMF conditions between 1980 and 2019 (Kentikelenis and Stubbs 2023).

These externally derived macroeconomic factors have domestic economic impacts in recipient countries. Focusing specifically on health financing, this paper examines the relationships between these external factors and the main domestic health financing sources in LMICs: government health expenditure as a source (GHE-S) and out-of-pocket payments (OOP) (WHO 2024).

The effects of development assistance for health sectors on domestic government health spending have been rather extensively studied (variably disaggregated as government health spending from domestic revenue or from all revenue), generally reporting a negative effect consistent with fungibility i.e. crowding out/displacement (Farag et al. 2009, Mishra and Newhouse 2009, Lu et al. 2010, Stuckler et al. 2011, Xu et al. 2011, Gebrehanna and Upadhyay 2012, Dieleman et al. 2013, Fernandes Antunes et al. 2013, Van de Sijpe 2013a, 2013b, Dieleman and Hanlon 2014, Liang and Mirelman 2014, Barkat et al. 2016, Younsi et al. 2016, Patenaude 2021). Several of these authors have shown that displacement only occurs when development assistance for the health sector is channelled through the recipient country government (Lu et al. 2010, Dieleman et al. 2013, Van de Sijpe 2013a, 2013b, Dieleman and Hanlon 2014) (also done by Patenaude 2021) (Patenaude 2021).

If on-budget development assistance to the health sector can displace GHE-S, then on-budget development assistance to non-health sectors might also displace government expenditure from those sectors to the benefit of the health sector. These relationships are much less studied. Some have found a positive effect of overall ODA per gross domestic product (GDP) on SSA governments’ share allocation to health sectors (Fosu 2007, 2010), while others found that general budget support (GBS) did not significantly affect GHE-S (Fernandes Antunes et al. 2013).

The existing literature on the relationship between development assistance and OOP is mixed. Some authors have found a negative relationship between external health spending and OOP in SSA (Frimpong et al. 2022), and negative relationships between development assistance for health (DAH) and different measures of OOP per household spending, though only when DAH was channelled via the government (Gabani et al. 2024). Others have found no relationship between DAH and OOP at the global level (Patenaude 2021), and that neither overall development assistance nor development assistance for the health sector per capita affects OOP when measured as a proportion of private health spending in SSA between 1995 and 2015 (Ali et al. 2020). Contrarily, some authors have found positive associations between DAH and OOP in LMICs (Younsi et al. 2016), and between external funds for health per capita and OOP per capita in lower middle-income countries (LMCs), with no significant correlation in low-income countries (LICs) and upper middle-income countries (UMICs) (Xu et al. 2011).

In terms of debt, the majority of the identified literature has found a negative relationship between indebtedness and government health spending (variably disaggregated as government health spending from domestic revenue or from all revenue). A study of 134 LMICs found a negative effect of a higher debt-to-GDP ratio on overall domestic government health financing from 2000 to 2015 (Patenaude 2021). Five studies from SSA (1975–94, 2000–14, and 2010–20) have found negative effects of public debt burden indicators on the government share allocation to the health sector, per capita government health spending, and GHE-S measured as a proportion of current health expenditure (CHE) (Fosu 2007, 2010, Said and Morai 2020, Chipunza and Ntsalaze 2024, Boundioa 2025). Variable effects were found (positive, negative, and insignificant) in another study of overall government debt servicing on government health expenditure across 85 LMICs from 2000 to 2013 depending on time subperiod and country subset specifications (Behera and Dash 2019). Conversely, a study of LMICs between 1995 and 2010 found positive associations between per capita general government debt and both government health expenditure (GHE) and GHE-S (Liang and Mirelman 2014).

For social spending, others have found that the general debt-to-GDP ratio but not interest payments was negatively correlated with social spending per GDP (50 LMICs, 1985–2003) (Lora and Olivera 2007). Across 84 LMICs from 1990 to 2010, debt servicing per GNI has also been found to be negatively associated with social protection expenditure per GDP (Murshed et al. 2022). In a selected panel of seven South and Southeast Asian countries from 1980 to 2010, another study found a negative link between debt servicing and social sector spending (Shabbir and Yasin 2015).

Studies of relationships between debt and OOP are limited, based on covariate analysis and robustness checks, and not specifically investigating the role of public *external* debt. One study has found no significant relationship between general government debt per GDP and OOP (Patenaude 2021), while another has found a negative relationship in SSA (exact variables not reported for this association) (Said and Morai 2020).

The effects of IMF programmes and loan conditionalities on health financing have been debated for decades. On one side are academics and civil society, generally criticizing austerity measures from the IMF for constraining government health financing based on quantitative econometric analysis, qualitative/mixed-methods policy analysis and opinion writing (Ooms and Schrecker 2005, Nooruddin and Simmons 2006, Center for Global Development 2007, Stuckler and Basu 2009, Foley 2010, Kingston 2011, Stuckler and Basu 2013, Abocejo 2014, Birn et al. 2016, Stubbs et al. 2017, Brunswijck 2018, Stubbs and Kentikelenis 2018, Meurs et al. 2019, Isiani et al. 2021, Kentikelenis and Stubbs 2024). There are however some exceptions to this (Kentikelenis et al. 2015, Ochs 2017, Daoud and Reinsberg 2019), and IMF and WB staff members have rejected the above findings and criticisms and themselves shown that health spending is protected/increases under IMF programmes (van der Gaag and Barham 1998, IMF Independent Evaluation Office 2003, Martin and Segura-Ubiergo 2004, Clements et al. 2011, Gupta 2017, IMF 2017) (though also with some exceptions Thomas 2006). To the best of our knowledge, associations between IMF conditionalities and OOP are unexplored.

None of the identified studies investigate all of our determinant variables of interest jointly, thus not allowing for a joint comparison of correlation sizes and directions. Such a comparison allows one to examine which of the examined macro-fiscal indicators most strongly determines dependence on GHE-S vs. OOP, and thereby which might be the most effective policy levers for pursuing universal health coverage (UHC). Most of the data used in the econometric literature is dated to the Millenium Development Goal era or before, and a newer exploration of these relationships into the Sustainable Development Goal era is warranted. In general, there are much fewer studies of associations between the examined macroeconomic indicators and OOP than for GHE-S. Additionally, studies infrequently specify dependent variables as proportions of CHE as we do in this study, and few studies discuss any equity implications of their findings. Any shift in the balance between the main sources of health financing in LMICs affects the degree of overall progressivity in the mix of health financing sources, i.e. the degree of alignment with ability to pay (Wagstaff et al. 1989, McIntyre and Mooney 2007, WHO 2010, Mtei et al. 2012, Martinez-Alvarez et al. 2020). This tends to be higher for tax-based contributions as compared with user fees (Wagstaff et al. 1989, McIntyre and Mooney 2007, World Health Organization 2010, Mtei et al. 2012, Martinez-Alvarez et al. 2020).

This in turn has implications for financial inequality, health service access, and health outcomes, particularly among poorer population groups (Makinen et al. 2000, Xu et al. 2007, WHO 2010, World Health Organization 2010, Chuma and Okungu 2011, Moreno-Serra and Smith 2011, 2015, Akazili et al. 2017, Qin et al. 2018, Boundioa 2025). By examining associations with the degree of reliance on GHE-S and OOP out of CHE, our study uses model specifications that explore relationships with direct relevance to UHC, the degree of progressivity of financing, and health service access for poor population groups in LMICs.

In this paper, we aim to investigate the relationships between development assistance, public external debt, and IMF loan conditionalities and the levels and balance of GHE-S and OOP. We do so by performing a cross-country panel data study of 105 LMICs between 2005 and 2019.

## Materials and methods

### Data sources, time period, and variables used

The data availability for our included variables allowed us to examine the time period 2005–19. The upper bound of our time period was determined by the IMF conditionality dataset (IMF Monitor 2023) and the lower bound by OECD CRS data separated by channel (OECD 2024). Our dataset included all countries that were counted as LMICs by the WB in October 2023 (*n* = 134) (World Bank 2024b), excluding countries with more than 5 years of missing data in our time period for health financing, development assistance, debt variables, IMF conditionality variables or GDP (*n* = 29), resulting in 105 countries (see Supplementary Appendix for full list). Data were compiled in a Microsoft Excel (Microsoft® 2024) spreadsheet and analysed in Stata (StataCorp 2015). Table 1 shows summary descriptive statistics for the variables used in our models.

**Table 1:** Descriptive statistics for variables used in models.

Variable	Obs	Mean	Std. dev.	Min	Max
OOP/CHE	1566	40.21	19.4	2.17	84.79
OOP/GDP	1566	2.17	1.4	.13	14.95
GHE-S/CHE	1566	40.23	19.05	4.16	80.5
GHE-S/GDP	1566	2.2	1.41	.14	6.82
ODA^+^ for health (public)/GDP	1573	.36	.59	0	6.34
ODA^+^ for health (civ./priv.)/GDP	1547	.16	.3	0	1.97
ODA^+^ for non-health (public)/GDP	1574	2.38	3.54	0	66.13
ODA^+^ for non-health (civ./priv.)/GDP	1574	.47	.78	0	7.19
PPG external debt service/GDP	1574	1.89	2.51	0	46.71
PPG external debt stock/GDP	1574	25.76	20.08	.12	232.42
IMF conditionalities	1575	12.48	20.08	0	122
IMF participation	1575	.35	.48	0	1
GDPpc	1574	3537.77	3053.67	200.36	15 432.25
IMR	1575	35.95	23.25	2.4	124.6
Government effectiveness	1575	−.57	.55	−2.32	1.16
Corruption control	1575	−.6	.52	−1.7	1.16
Conflict	1575	.17	.38	0	1
Colonial independence from UK	1575	.26	.44	0	1
Colonial independence from France	1575	.22	.41	0	1
Colonial independence from Spain	1575	.06	.23	0	1

#### Variables and data sources

**Figure 2:**
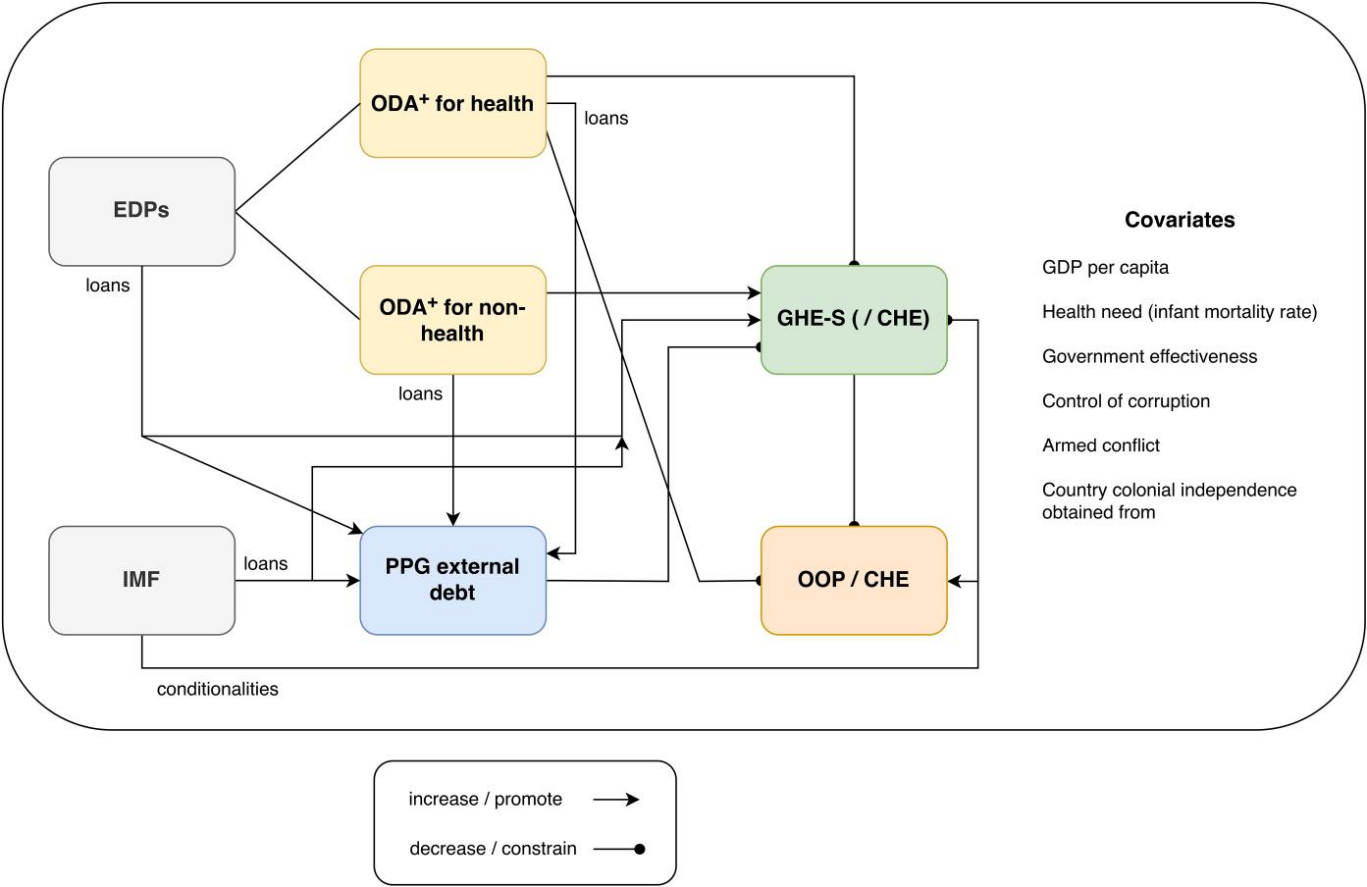
Main hypothesized relationships for this study.

Figure 2 displays the main relationships hypothesized for this study. The different relationships are explained in the below sections.

*Dependent variables*. We used the following dependent variables $Y_{it}^{\prime }$.

OOP per CHE in a given country *i* in year *t* (in %) (model 1). A higher proportion means that in relative terms, more of a country’s health financing comes directly from users at the point of care, relative to other sources of health financing.

OOP per GDP (in %; natural logged) (model 2). This variable measures how much households in a country spend on health services out of pocket relative to the overall size of the economy.

GHE-S per CHE (in %) (model 3). This variable measures the level of government health spending out of its own revenue, excluding external transfers, relative to total CHE.

GHE-S per GDP (in %; natural logged) (model 4). This measures the level of government health spending out of its own revenue, excluding external transfers, relative to the overall size of the economy.

Changes in the per CHE-variables reflect relative changes in the burden of payment for health services. Increases can reflect relative decreases in other health financing sources and vice versa, including faster and slower growth rates, and they do not provide any information about the absolute amount of financing.

Increases in the per GDP-variables can also reflect relative decreases in GDP and vice versa, and faster and slower growth rates. However, because year-on-year GDP growth rates were positive in most years in our dataset, an increase on average in a health financing source relative to GDP will reflect a real expansion of health sector activity financed by OOP or GHE-S, respectively.

We used health financing data from the World Health Organization (WHO) Global Health Expenditure Database (WHO 2024) and GDP data from the WB (WHO 2024, World Bank 2024b).

*Independent variables*. Our independent variables included the following: ODA plus disbursements from the Bill and Melinda Gates Foundation (BMGF), together ODA^+^ (Arregoces et al. 2015), which counts ODA disbursements (minus debt relief, administrative expenses, in-donor country expenses, and promotion of development awareness) plus BMGF grants for health and non-health purposes respectively, measured per GDP (in %; natural logged; lagged). Following the majority of the identified literature, we mainly hypothesized a displacement/crowding out effect of ODA^+^ for health on GHE-S (i.e. fungibility) as well as on OOP. Also following the literature on government health spending (Lu et al. 2010, Dieleman et al. 2013, Van de Sijpe 2013a, 2013b, Dieleman and Hanlon 2014, Patenaude 2021), we hypothesized that a fungibility dynamic would depend on whether the development assistance is ‘on-budget’ or ‘off-budget’, which determines whether or not the government can see and predict development assistance coming in and adjust its own budget accordingly. We therefore disaggregated our ODA^+^-variables into development assistance channelled via the recipient country public sector, and channelled via non-governmental organizations, civil society organizations, or private sector institutions, jointly referred to as the civil/private sector. For OOP as a dependent variable, we mainly hypothesized that ODA^+^ for health via either channel would have an overall displacing effect, as either could work to subsidize or cover user fees. This would mainly manifest as decreased OOP/CHE, as ODA+ for health forms part of CHE, while changes in OOP/GDP are harder to predict due to growth-promoting effects of ODA^+^ for health through improved population health (Bloom et al. 2004). Decreases in OOP/CHE could, however, be (partially) counteracted by a displacement effect from ODA^+^ for health on GHE-S/CHE. Our main hypothesis for OOP was generated on the background of a diverging literature (Xu et al. 2011, Younsi et al. 2016, Ali et al. 2020, Patenaude 2021, Frimpong et al. 2022, Gabani et al. 2024).

We also examined non-health ODA^+^ (OECD 2024) to explore whether development assistance outside the health sector is associated with changes in government and household health financing (Feyzioglu et al. 1998, Ali et al. 2020). The main hypothesis tested was that of non-health sectoral aid benefiting the health sector by allowing governments to shift some extra funds towards the health sector, i.e. that on-budget non-health ODA^+^ would correlate positively with GHE-S per GDP and/or per CHE. However, if GDP growth effects are equal to or stronger than any positive effects on GHE-S, no correlation/negative correlation would be seen for GHE-S/GDP (we also run a GHE-S/capita analysis as sensitivity analysis in the Supplementary Tables A32–39). Importantly, the variables also control for the macroeconomic effects of non-health aid flows.

Using two-sided *T*-tests, we allowed for the alternative hypotheses that ODA^+^ for health could crowd in GHE-S and that ODA^+^ for non-health sectors could draw domestic government funds away from the health sector, e.g. through co-financing requirements.

Data on ODA were obtained from the Organisation for Economic Cooperation and Development (OECD) Creditor Reporting System (CRS) database (OECD 2024).

We included public and publicly guaranteed (PPG) external debt service and stock, respectively per GDP (in %; natural logged; lagged). In theory, increased debt servicing should take away money from the public budget, reducing the fiscal space for all government expenses including the health sector, leading to our main hypotheses of a negative association between PPG external debt servicing and GHE-S/GDP and/or GHE-S/CHE. Alternatively, the ability to service one’s debt could reflect favourable fiscal conditions and/or occur at a manageable level posing no risk of ‘debt distress’, with enough revenue to finance both debt and health, which would be supported by the confirmation of our null-hypothesis of no significant association. We further take into consideration the PPG external debt stock per GDP (World Bank 2024a), similar to Lora and Olivera (2007) (Lora and Olivera 2007). This allows us to adjust for the level of indebtedness, i.e. adjusted for the size of the debt relative to the size of the economy, are increases in debt servicing per GDP associated with decreases in GHE-S/GDP or GHE-S/CHE, and associated increases in OOP/GDP or OOP/CHE? It also allows us to examine any associations between the debt stock itself and our outcome variables (Lora and Olivera 2007). Debt variable data were obtained from the WB International Debt Statistics database (World Bank 2024a).

We included the number of binding and non-binding IMF conditions in place in a given country-year. More conditions could lead to less GHE-S generally through what can be referred to as an austerity effect (Ooms and Schrecker 2005, Center for Global Development 2007, Stuckler and Basu 2009, Foley 2010, Kingston 2011, Stuckler and Basu 2013, Abocejo 2014, Birn et al. 2016, Stubbs et al. 2017, Brunswijck 2018, Meurs et al. 2019, Isiani et al. 2021), which was our main hypothesis. IMF/WB authors have conversely found that they could lead to more GHE-S (van der Gaag and Barham 1998, Martin and Segura-Ubiergo 2004, Thomas 2006, Clements et al. 2013, Gupta 2017), meaning that conditions lead to growth and more fiscal space for health, and that health spending is protected from any austerity measures. As for OOP, we hypothesized that privatization and decentralization measures resulting from IMF conditionalities, as well as an indirect effect via the above austerity effect where the state funds less health services and users have to take over payment, could lead to increases in OOP. To isolate the marginal effect of an additional conditionality within an IMF programme, we included a dummy variable for IMF programme participation, generated from the conditionality variable (‘on’ when conditions are observed) (Forster et al. 2020). This also captures any effects of IMF programme participation separate from those acting through conditionalities, such as catalyzing development assistance and investment, or effects of technical assistance or other means of policy influence (Stubbs et al. 2016, Forster et al. 2020, Kentikelenis and Stubbs 2023).

IMF variable data were obtained from the IMF Monitor conditionality dataset (IMF Monitor 2023). To limit hypothesis proliferation, multiple comparisons, and multicollinearity, our models did not include disaggregated variables for lending, neither as ODA, or from the IMF. Contributions from these flows to PPG external debt are, however, captured in our models, as illustrated in Fig. 2. Figure 2 also shows how IMF loans could increase the fiscal space for health either through direct budget support or through expanding the government revenue basis (again not separately included for the above reasons).

*Covariates.* We adjusted our models using a set of covariates.

GHE-S per GDP (in %, natural logged) (WHO 2024) was included for models with OOP as the dependent variable, as government health spending may co-determine household OOP spending (Xu et al. 2011, McIntyre et al. 2017, Ahmed 2025).

We included GDP per capita (natural logged, constant 2020 US$) (World Bank 2024b), as this may co-determine both our outcome variables, independent variables of interest, and covariates.

We included infant mortality rate (IMR), defined as the number of children dying in their first year of life per 1000 live births, as a proxy for unmet population health need (Feyzioglu et al. 1998, Reidpath and Allotey 2003, Lee and Lim 2014), from the UN Inter-agency Group for Child Mortality Estimation (2024). Such an unmet need may reflect a low GHE-S and a high resulting reliance on OOP, as well as health needs driving OOP health expenditures. IMR has also been identified as the strongest health need determinant of DAH allocations, above under-5 mortality rate (U5MR) and human immunodeficiency virus prevalence (Lee and Lim 2014).

We included a variable capturing government effectiveness and a variable measuring control of corruption from the Worldwide Governance Indicators database (Kaufmann and Kraay 2023). Government effectiveness is a measure of the perceived quality of government, public and civil services, policy formulation and implementation, while corruption control is defined as the perceived degree of exercise of public power for private gain and state capture by elite and private interests (Kaufmann and Kraay 2023). Both were measured in standard deviations (Kaufmann and Kraay 2023). These are commonly used in the development assistance literature as they are found to co-determine aid flows and aid effectiveness, including development lending (e.g. Burnside and Dollar 2004, Quazi 2005, Nanda 2006, In’airat 2014, Oryema et al. 2017, Sterck et al. 2018, Jung et al. 2024). A country’s governance also results from and determines IMF programme participation, policy recommendations, and conditionalities (IMF 2024a). More corrupt governments have been found to spend less on health (Hashem 2014, Jajkowicz and Drobiszová 2015), and the same is true for less effective governments (Kaufmann and Kraay 2023, WHO 2024). These variables might also help explain why development assistance and GHE fail to translate into reduced dependency on OOP health spending.

We adjusted for the effects of a country being in armed conflict, by including a dummy variable for when the number of deaths in battle (We replaced missing values for battle-related deaths with 0’s, as this dataset only report recorded battle-related deaths and assign missing values to all other country-years.) was larger than or equal to 1 per million population in a country-year (1 when true, 0 when not), using the Uppsala Conflict Data Program/Peace Research Institute Oslo Battle-Related Deaths Dataset (Davies et al. 2023). Development assistance and lending flows have by some been found to differ (Yogo and Mallaye 2012, World Bank 2014), and by others not to differ (Collier and Hoeffler 2002), between countries depending on conflict status. Receiving development assistance has been found to be associated with a higher likelihood of conflict escalation (Bluhm et al. 2016), and GHE has been found to suffer in war-ridden countries (Mishra and Newhouse 2009). We further hypothesized that conflict might cause an increased reliance on OOP health spending in a destabilized health economy.

Finally, we examined for differences in the effects of colonial legacy between former British, French, and Spanish colonies. Bilateral donors tend to give more development assistance to their former colonies (e.g. Alesina and Dollar 2000, Neumayer 2003, Dietrich 2011, Matteis 2016), and the economies and institutions of formerly colonized nations are partly shaped by their colonial legacy (Grier 1999, Acemoglu et al. 2001, Jones 2013), including their health system models (Azevedo 2017). We hypothesized that this could also co-determine health system payment patterns, both by system inheritance and by ongoing policy influence from the former colonizer. We coded this as three dummy variables using the Issue Correlates Of War dataset (Hensel 2023).

#### Variable transformations

As per above, all variables showing a skewed distribution were natural-log transformed. This (i) normalized their distribution, and (ii) eased economic interpretation as elasticities. All financial variables except per CHE-variables entered models as proportions of GDP, similar to other authors, e.g. (Feyzioglu et al. 1998, Lora and Olivera 2007, Lu et al. 2010, Dieleman and Hanlon 2014) (in %; natural logged). Our resulting health financing measures thereby reflect real expansions/contractions of health financing sources beyond what would be expected simply as a result of background changes in the size of the economy and following inflation levels. The effects of absolute aid and debt flows may also greatly depend on at what level relative to the size of the economy they occur. By dividing OOP and GHE-S by GDP, the introduction of spurious correlations from dividing dependent and independent financial variables with the same deflator numbers was avoided, which would have been necessary to adjust for inflation, if financial variables were measured as absolute amounts or per capita (Kronmal 1993). It also meant that a population covariate could be omitted (necessary in the case of measuring variables directly), increasing parsimoniousness and degrees of freedom and reducing the risks of multicollinearity, instrument proliferation and overidentification. Measuring directly would also make some economic interpretations less meaningful (e.g. ‘Having an IMF programme was associated with an X$ higher/lower level of OOP expenditure’, or X% if log transformed).

The use of per CHE-variables has been motivated in the introduction.

### Econometric methods

#### Full model specification

Our initial full model specification was as follows:


$$\begin{array}{rl} Y_{it}\prime = & \;\beta_{0}+\beta_{1}\prime Y_{i,t-1}\prime +\beta_{2}\prime \ln{\left(\frac{\mathrm{OD}A^{+\;}\;\mathrm{for}\;\;\mathrm{health}}{\mathrm{GDP}}\right)}_{i,t-1}\prime \\ & +\beta_{3}\prime \ln{\left(\frac{\mathrm{OD}A^{+\;}\;\mathrm{for}\;\;\mathrm{non}\text{-}\mathrm{health}}{\mathrm{GDP}}\right)}_{i,t-1}\prime \\ & +\beta_{4}\ln{\left(\frac{\mathrm{PPG}\;\;\mathrm{external}\;\;\mathrm{debt}\;\;\mathrm{service}}{\mathrm{GDP}}\right)}_{i,t-1}\\ & +\;\beta_{5}\ln{\left(\frac{\mathrm{PPG}\;\;\mathrm{external}\;\;\mathrm{debt}\;\;\mathrm{stock}}{\mathrm{GDP}}\right)}_{i,t-1}\\ & +\;\beta_{6}\mathrm{IMF}\;\;\mathrm{conditionalitie}s_{it}+\beta_{7}\mathrm{IMF}\;\;\mathrm{participatio}n_{it}\\ & +\beta_{8}\ln{\left(\frac{\mathrm{GHES}}{\mathrm{GDP}}\right)}_{it}+\;\beta_{9}\ln(\mathrm{GDPpc}{)}_{it}+\beta_{10}\mathrm{IM}R_{it}\\ & +\beta_{11}\mathrm{gov}.\;\mathrm{effectivenes}s_{it}+\beta_{12}\;\mathrm{corruption}\;\;\mathrm{contro}l_{it}\\ & +\beta_{13}\mathrm{conflic}t_{it}+{\;\beta }_{14}\prime \;\mathrm{colonial}\;\;\mathrm{independence}\;{\;\mathrm{from}}_{it}^{\prime }\\ & +\mu_{i}+\nu_{t}+\varepsilon_{it} \end{array}$$

$\mu_{i}$ is unobserved, country-specific, time-invariant effects (country fixed effects, e.g. geography), $\nu_{t}$ is unobserved, cross-country, time-variant effects (year fixed effects, e.g. global shocks, included as year dummies), and $\varepsilon_{it}$ is the error term.

#### Variable reduction

Our full model specification was the product of a comprehensive and exploratory literature review, testing multiple hypotheses at once, some of which turned out not to be supported by the results. We resultingly ran backward stepwise regressions to generate reduced models, sequentially removing independent variables with a *P*-value > .2, starting with the variable with the highest *P*-value. This was done to (i) increase parsimoniousness, (ii) reduce the risk of type II error due to multicollinearity, (iii) minimize the issue of instrument proliferation, (iv) avoid overfitting, and (v) lower the risk of significant findings being due to multiple comparisons (Roodman 2009a, 2009b, Kiviet 2020). All covariates were still kept in these models and then changed/removed in the respective sensitivity analyses, yielding parsimonious models that identified significant relationships while still adjusting for covariates. The resulting reduced form models are presented in Table 2. The full model regression results are presented in the Supplementary Table A1.

**Table 2:** Two-step system GMM results for reduced form model specifications.

Dependent variable	(1a) OOP/CHE	(2a) Ln(OOP/GDP)	(3a) GHE-S/CHE^a^	(4a) Ln(GHE-S/GDP)
L. Dependent variable	0.662*** [0.059]	0.928*** [0.061]	0.710*** [0.080]	0.694*** [0.082]
L2. Dependent variable	—	—	0.229*** [0.075]	
L. ln[ODA^+^ for health (public)/GDP]	−0.412 [0.268]	−0.024** [0.010]	—	−0.026** [0.013]
L. ln[ODA^+^ for health (civ./priv.)/GDP]	−0.339 [0.215]	−0.012** [0.006]	—	—
L. ln[ODA^+^ for non-health (civ./priv.)/GDP]	−	−0.038** [0.018]	0.789* [0.467]	—
L. ln(PPG external debt service/GDP)	0.725** [0.350]	—	−0.664** [0.287]	—
ln(GHE-S/GDP)	−8.228*** [1.628]	0.072** [0.036]	—	—
ln(GDPpc)	−1.340 [1.801]	−0.251*** [0.074]	1.395 [2.191]	0.110 [0.089]
IMR	−0.144*** [0.053]	−0.003* [0.002]	−0.019 [0.058]	−0.001 [0.003]
Gov. effectiveness	1.144 [1.420]	−0.002 [0.045]	−1.144 [1.237]	−0.030 [0.063]
Corruption control	−2.241 [1.617]	0.063 [0.059]	3.180** [1.310]	0.054 [0.078]
Conflict	0.387 [0.748]	0.039* [0.021]	−0.064 [0.552]	0.037 [0.031]
Colonial ind. from UK	−1.966 [1.486]	−0.019 [0.045]	0.180 [0.941]	0.041 [0.077]
Colonial ind. from France	0.274 [1.589]	0.043 [0.043]	0.461 [1.164]	−0.083 [0.063]
Colonial ind. from Spain	−0.303 [1.626]	0.018 [0.056]	1.842 [1.150]	−0.004 [0.089]
Constant	30.492* [16.901]	1.890*** [0.608]	−4.797 [17.207]	−0.697 [0.763]
Observations	1429	1429	1356	1460
Countries	105	105	105	105
Instruments	77	97	88	73
Lag limits (years)	5	7	8	7
Country fixed effects	Yes	Yes	Yes	Yes
Year fixed effects	Yes	Yes	Yes	Yes
AB-test AR(2) (p-level)	0.944	0.785	0.659	0.194
Hansen test (p-level)	0.402	0.469	0.242	0.346
Diff.-in-Hansen test (p-level)	0.150	0.543	0.908	0.725
F-statistic	495.52***	128.67***	1794.7***	117.58***

AB-test AR(2), Arellano-bond test for first-order autocorrelation in the levels equation. Diff.-in-Hansen test, difference-in-Hansen test for exogeneity of GMM instruments in levels equation. Govt., government. IMR, infant mortality rate. Ind., independence (colonial). L., 1-year lag. L2., 2-year lag. Ln, natural logarithm.

^a^Second-order lag of dependent variable included as independent variable in model 3a to avoid serial correlation as determined by the AB-test.

Windmeijer-corrected robust standard errors in brackets; *P < .1, **P < .05, ***P < .01. We used a significance level of 0.05 for all regressions and associated discussions, while results significant at the 0.10-level were only discussed for sensitivity tests where main model specification results were significant at the 0.05-level, except for the case of contemporaneous IMF programme participation (Supplementary Appendix), which warranted separate discussion.

#### Estimation strategy

*Generalized method of moments*. To overcome serial autocorrelation and heteroskedasticity (see Supplementary Appendix), we adopted two-step system GMM estimation (Hansen 1982, Arellano and Bond 1991, Arellano and Bover 1995, Blundell and Bond 1998, Hall 2005, Roodman 2009a), using fixed effects following the Hausman test (Hausman 1978), employed with the Stata command ‘Xtabond2’ (Roodman 2009a). The two-step GMM is a more efficient estimator and has been designed for panel data analysis with a relatively small number of time periods and a larger number of units of observation (countries) (Blundell and Bond 1998, Roodman 2009a), as in our case.

All explanatory variables except colonial independence variables and year dummies were treated as endogenous (GMM-style instrumentation), while colonial legacy and year-dummies were treated as exogenous (instrumental variable (IV)-style instrumentation) (Roodman 2009a, Kripfganz 2019, Kiviet 2020). We used the Windmeijer-correction to correct for the tendency of two-step system GMM to produce downward-biased standard errors (Windmeijer 2005, Roodman 2009a). To lower the instrument count, we collapsed the instrument matrix (Roodman 2009a, 2009b, Kripfganz 2019, Kiviet 2020). We also applied upper bounds to the number of year lags used for GMM-style instrumentation (the so-called lag limit; Roodman 2009a), and ensured that each model was valid by adjusting the lag limit, guided by the instrument count and the following tests.

We used the Arellano–Bond test to check for first-order autocorrelation in the levels equation (Arellano and Bond 1991, Roodman 2009a, Kripfganz 2019, Kiviet 2020). We used the Hansen test (Hansen 1982) to explore the validity of our generated moment conditions and avoiding overidentification, ensuring *P*-values between .15 and .6 (Roodman 2012, Adeleye 2019, Kiviet 2020). We used the difference-in-Hansen test to ensure exogeneity of the generated GMM-style instruments in the levels equation (Roodman 2009a, 2009b).

Regression outputs were exported using the Stata command ‘Asdoc’ (Shah 2018) and are available in the Supplementary Appendix.

#### Lag structure

Our independent variables of interest could have some delayed effects on our dependent variables. We therefore systematically explored different lag structures by first specifying 2-year lags for all of these and then sequentially reducing the lag level (Kripfganz 2019, Kiviet 2020). This process showed that a parsimonious lag specification of 1-year lags for all financial independent variables of interest generally, with a few exceptions mentioned in the Results section, identified the highest level of significance and optimized the above test statistics. This specification type follows the work of other authors in our field, e.g. (Lora and Olivera 2007, Mishra and Newhouse 2009, Stubbs et al. 2017, Forster et al. 2020). Its consistency and parsimoniousness also limited confirmation bias and selective reporting of significant results at various lags. Economically, a 1-year lag of the financial variables of interest also made sense, as the fiscal implications of these macroeconomic factors may not be realized until the following year. When a government prepares its expenditure budget for the coming year, it does so based on its current income and expenses adjusted by future expectations. For example, when ODA arrives on the budget, some of this may not be programmed as sectoral expenses until the following year. IMF conditionalities and IMF programme participation, however, apply to the current financial year, they did not show the same significance patterns for their lags, and were therefore kept unlagged.

Further details of this process are provided in the Supplementary Appendix, along with our approach to the issue of serial correlation and diagnostic tests performed.

#### Sensitivity analysis

*Alternative lag structures.* To examine alternative durations of delay in the effects of independent variables on dependent variables, we ran our models with no lags (i.e. no delay) and with 2-year lags on the independent variables of interest.

We also examined the robustness of our results to changing the number of generated GMM-style lags by altering the lag limit (Roodman 2009a, 2009b, Kripfganz 2019).

*Alternative variables and transformations.* We ran our models with all financial variables, including dependent variables, being per capita instead of per GDP, as is often also done in the literature (e.g. Mishra and Newhouse 2009, Liang and Mirelman 2014). We used the same US$ Consumer Price Index data for deflation of all per capita financial variables (World Bank 2024b).

We tried swapping IMR with U5MR (UN Inter-agency Group for Child Mortality Estimation 2024) and maternal mortality ratio (MMR) as alternative health need indicators (WHO et al. 2023).

We ran our models without log-transformation.

We explored the effect of using alternative measures of conditionality, i.e. including only binding conditions or not, and attributing different weights to binding vs. non-binding conditions, as binding conditions could be more influential than non-binding conditions (IMF Monitor 2023).

To adjust for any regional and income group effects on our dependent variables, we ran our models with a regional dummy for SSA instead of colonial independence dummies; with LMC and LIC WB income group dummies instead of colonial independence dummies; and without any of these dummies altogether, as they were generally found to be insignificant.

We tried aggregating our ODA-variables into just health and non-health purposes, as was done in some of the earlier fungibility literature (e.g. Mishra and Newhouse 2009).

*Interaction terms.* We explored a battery of interaction terms between our main independent variables of interest and the region being SSA, being a LIC, having a higher degree of government effectiveness, having less corruption, and having a higher level of GHE-S relative to GDP. These tested the respective sets of hypotheses that ODA^+^ for health, PPG external debt service, and IMF conditionality could have different effects on GHE-S and OOP under these five conditions. For example: ODA^+^ for health could displace GHE-S more in SSA compared with other regions; debt service could shift the burden of payment more from the government onto the user in LICs; more effective governments could be subjected to milder conditionality with less impact on health financing—or could implement conditionality more effectively resulting in a larger impact; ODA^+^ for health could be more effective at reducing OOP in less corrupt countries; and debt servicing could have differential impact on the health system payment pattern at different levels of GHE-S.

*Additional checks.* We tested the effect of using US GDP deflator data instead of US CPI data for deflation (World Bank 2024b).

We tried adding Inverse Mills Ratios to our full models to adjust for any selection bias for IMF programme participation present even at a non-significant level (i.e. *P* > .05).

## Results

Table 2 shows the results of our four final reduced form model specifications. The results of our full model specifications are available in the Supplementary Online Appendix (Supplementary Table A1), and these are described in the Sensitivity analysis section. Findings are summarized for each set of independent variables of interest along with sensitivity testing. Significant findings for our covariates are described in the Supplementary Online Appendix.

### Official development assistance

In our analysis of ODA variables, we found some evidence that ODA+ for health channelled via the recipient country public sector was significantly associated with a reduction in OOP expenditures as well as a decrease in GHE-S measured as a share of GDP (evidence of displacement of both). Specifically, we found that a 1% increase in the lag of ODA+ for health purposes disbursed via the recipient country public sector per GDP was associated with a −0.024% reduction of OOP/GDP (*P* = .02), and a −0.026% decrease in GHE-S/GDP (*P* = .045). When channelled via the civil/private sector, the negative association with OOP/GDP remained but not with GHE-S/GDP, i.e. there was no evidence of off-budget ODA+ for health leading governments to lower GHE-S. We also did not find any evidence for our hypothesized positive effect of on-budget ODA+ for other purposes than health on GHE-S. We found no significant association between ODA+ for health via the public sector per GDP and OOP/CHE or GHE-S/CHE. Overall, correlation sizes were small.

### Debt

For debt, we found increases in PPG external debt servicing levels to correlate with small increases in OOP/CHE and small decreases in GHE-S/CHE (evidence of a small shift in the burden of payment from government to user). Specifically, we found that a 1% increase in lagged PPG external debt servicing per GDP was positively associated with a 0.007%-point increase in OOP/CHE (*P* = .041) and a −0.007%-point decrease in GHE-S/CHE (*P* = .023).

Lagged PPG external debt servicing did not correlate with the remaining dependent variables. The level of PPG external debt stock per GDP was not associated with any dependent variables. Again, correlation sizes were small.

### International Monetary Fund programmes and conditionalities

IMF programme participation and the number of IMF conditionalities were removed during our model reduction process, as they were not found to significantly influence any dependent variables in our full models or in sequential model reduction steps.

### Sensitivity testing

#### Full models, alternative lag specifications, and covariate swaps

Our significant results for ODA-variables were robust to some specification changes but not to others. The significant negative association between on-budget ODA^+^ for health purposes per GDP with GHE-S/GDP remained in our full model, but not for OOP/GDP (*P* = .086) (Supplementary Table A1).

Our findings that increased on-budget ODA^+^ for health per GDP was associated with significant reductions in both OOP and GHE-S per GDP were also present at the 2nd lag, but not in a contemporaneous specification (Supplementary Tables A18 and A22). The negative correlation with OOP/GDP was robust to increasing the GMM-style lag limit, but not to decreasing it, and it was robust to most but not all covariate swaps (Supplementary Tables A28 and A36). The negative correlation with GHE-S/GDP was only robust to increasing the lag limit by 1 but not to decreasing it, however, it was robust to all covariate swaps (Supplementary Tables A30 and A38).

In the alternative specification measuring our variables per capita, we did not find evidence of displacement of GHE-S by on-budget ODA^+^ for health. In this specification, we instead found that per capita ODA^+^ for non-health purposes provided via the public sector had a significant positive association with GHE-S per capita in our full model only (Supplementary Table A34).

The significant positive correlations between lagged PPG external debt servicing and OOP/CHE and negative with GHE-S/CHE were sensitive to some model modifications with the latter being more robust. The significantly positive association between lagged PPG external debt servicing per GDP and OOP/CHE was not robust to changes in lag limits, and only robust to excluding/swapping colonial history variables but not mortality indicators (Supplementary Tables A27 and A35). The negative association with GHE-S/CHE was robust to increasing, but not decreasing lag limits, and it was robust to all covariate swaps (Supplementary Tables A29 and A37). None of our debt variables were significant at the 2-year lag or unlagged. The variables were insignificant in full models (the positive OOP/CHE correlation for debt servicing was near-significant at *P* = .057).

We found a borderline significant negative association between IMF programme participation and GHE-S/CHE only in our full model (*P* = .08), which was significant in a contemporaneous full model version (−1.13%-point decrease from IMF participation (*P* = .029) (Supplementary Tables A1 and A15). In this model version, the finding was still present at GMM-style lag limits 3, 4, and 5, but not 2 and 6 (model overspecified at lag limit 6—Supplementary Table A31). It was robust to most covariate swaps (Supplementary Table A39). We checked for reverse causation, which was not present. IMF variables were not significant at the first or second lag and did not become significant by modifying lag limits.

Findings from our additional robustness checks are described in the Supplementary Online Appendix.

## Discussion

The objective of this study was to explore the relationships between external development financing, public external debt, loan conditionalities from the IMF, and key recipient country health financing sources. We discuss our main findings in relation to the literature in the below sections. Policy implications and research recommendations are discussed in the Conclusions section.

### ODA^+^ for health and health financing sources

Our first main finding was that both lagged on-budget and off-budget ODA^+^ for health were associated with reductions in OOP/GDP, but not OOP/CHE. This offers some evidence that these types of assistance from external development partners (EDPs) modestly displace or subsidize OOP, but do not do so in a manner in which the degree of reliance on OOP out of total CHE is reduced. The latter could at least in part be explained by the finding that on-budget ODA^+^ for health also displaces GHE-S (measured per GDP) with a negative elasticity of a comparable magnitude, although associations with other health financing sources were not investigated.

These findings broadly align with results from two previous studies. One panel study showed a negative relationship between external health spending and OOP in SSA (Frimpong et al. 2022), and a fixed-effects/pseudo-panel study based on household survey data among 65 LMICs showed a negative relationship between on-budget DAH (DAH-G) per capita and health OOP per total household spending (Gabani et al. 2024). Compared with the latter study, our findings, however, extend to also show a negative relationship between off-budget development assistance to the health sector and OOP, both measured per GDP.

Our findings, however, differ from other authors, but methodological differences readily explain this, and direct comparisons are difficult to make. Studies have found no association between DAH-G or DAH-NG and OOP on either a log-log scale or level scale (Patenaude 2021), and significant positive relationships between total DAH and OOP measured directly (i.e. without denominator) (Younsi et al. 2016) and between external funds for health/cap (essentially DAH/cap) and OOP/cap only in lower-MICs (Xu et al. 2011).

All of the cited studies differ internally and from ours in model specification, variable transformation, country inclusion and grouping choices, time period, and extent of diagnostic and sensitivity testing performed. Other authors have measured OOP as a proportion of private health spending, which again renders direct comparisons challenging (Ali et al. 2020). Our finding was not fully robust to sensitivity tests, and we resultingly advise some caution when interpreting this finding and its potential policy implications.

Our second main finding was that lagged ODA^+^ for health channelled via the recipient country public sector was associated with reductions in GHE-S/GDP. This association was not found when ODA^+^ for health was channelled outside of government or when measured jointly, i.e. irrespective of channel. This supports the interpretation of a modest displacing effect specifically of on-budget ODA^+^ for health on GHE-S (fungibility). This finding aligns with the majority of the extensive, and at times conflicting, body of literature on the fungibility of development assistance to the health sector (Farag et al. 2009, Mishra and Newhouse 2009, Lu et al. 2010, Stuckler et al. 2011, Xu et al. 2011, Gebrehanna and Upadhyay 2012, Dieleman et al. 2013, Fernandes Antunes et al. 2013, Van de Sijpe 2013a, 2013b, Dieleman and Hanlon 2014, Liang and Mirelman 2014, Barkat et al. 2016, Younsi et al. 2016, Patenaude 2021). In particular, the fact that we were unable to identify fungibility when not distinguishing between on- and off-budget DAH aligns with similar/analogous findings by others (Lu et al. 2010, Dieleman et al. 2013, Van de Sijpe 2013a, 2013b, Dieleman and Hanlon 2014, Patenaude 2021). A government can only respond to what it can see, and if ODA^+^ for health is distributed directly from an EDP to the civil or private sector, this may not be in clear view for the government and may thus not trigger any fiscal redistributions.

The caveat needs to be made that some of the above literature has faced methodological criticism (Lu et al. 2010, Roodman 2012). Again, our finding was not fully robust to all sensitivity tests, and the correlation size was small. We have, however, subjected the fungibility hypothesis for on-budget ODA^+^ for health to extensive scrutiny, going beyond existing studies, and our main findings are confirmatory, though at a considerably lower level than previous studies.

Our full model finding that per capita ODA^+^ for non-health purposes provided via the public sector had a significant positive association with GHE-S per capita would support the interpretation that external funding injections to other public sectors allow governments to shift some additional funds to the health sector. Our main reduced specification, however, did not confirm this relationship. This highlights the sensitivity of these types of models to variable choices. The fungibility hypothesis for on-budget development assistance to the health sector is now well supported in the literature, and future research could further scrutinize its inverse: if development assistance to non-health sectors free up domestic funds to the benefit of the health sector.

### Debt and health financing sources

We found evidence that increases in lagged public external debt servicing levels per GDP were associated with relative increases in OOP and relative decreases in GHE-S measured as proportions of CHE. The association with GHE-S/CHE was more robust than that for OOP/CHE, and neither was fully robust to all sensitivity tests. The fact that OOP/CHE was found to increase by a similar amount as GHE-S/CHE decreased points to the possibility of changes in OOP/CHE being mediated by changes in GHE-S/CHE. Overall, these findings provide some novel evidence in support of the hypothesis that increasing public external debt servicing reduces the remaining envelope for other government expenses, including for public health, resulting in a shift in the burden of payment for health services from the government towards the user. This can be seen as worsening the overall progressivity of health financing. On average, OOP and GHE-S each constituted 40% of CHE across our sample of country-years, meaning our dependent variables reflect the bulk of health financing across the studied LMICs and years.

The associations seen were, however, not identifiable when measuring GHE-S and OOP per GDP or per capita, which challenges the above interpretations, unless the effects from debt are indeed more compositional and relative in terms of government budget allocations and overall health system payment patterns, but neutral in proportion to increases over time in economic output and population.

This part of our findings broadly align to some extent with the majority of the relevant identified econometric literature, finding negative associations between debt indicators and government health spending indicators (Fosu 2007, 2010, Said and Morai 2020, Patenaude 2021, Chipunza and Ntsalaze 2024, Boundioa 2025), as well as with social spending indicators (Lora and Olivera 2007, Shabbir and Yasin 2015, Murshed et al. 2022) [Lora and Olivera (2007)  *‘*found the general debt-to-GDP ratio but not interest payments to be negatively correlated with social spending per GDP across 50 LMICs from 1985 to 2003’ (Lora and Olivera 2007)]. Variable associations with GHE have been found in one study (Behera and Dash 2019) and positive in another (Liang and Mirelman 2014).

One of these studies at the global level found no significant association between the general government debt-to-GDP ratio and OOP (Patenaude 2021), while another from SSA reported negative relationships between debt indicators and OOP as a robustness check, though without showing those regression results (Said and Morai 2020).

Differences between previous findings and ours may likely be due to significant differences in data used, model specification, and estimation technique, and we have addressed some methodological issues that may have been present in previous studies, including instrument proliferation. Viewed jointly, ours and other studies in the econometric literature mostly point to a negative link between debt and government health financing. However, the econometric evidence available, ours included, should be seen as one type of evidence improving our understanding of these relationships, which can then be supplemented by other types of evidence, e.g. country-case studies.

### International Monetary Fund programme participation, conditionalities, and health financing sources

We found no association between IMF programme participation or the number of IMF conditionalities and our dependent variables in our main models. Only in a contemporaneous full model version did we find a partially robust, significant negative association between IMF participation and GHE-S/CHE, and not with GHE-S/GDP (Supplementary Tables A15 and A16). Viewed jointly, our findings do not convincingly support the hypothesis of a constraining effect of IMF programmes and conditionalities on GHE-S, nor of a promotion of OOP.

As with the above literature, there are numerous differences in data used, specification choices, and estimation methods amongst previous studies of IMF effects on health financing, with equal divergence in the empirical findings from econometric studies. Our mostly negative findings align most with IMF/WB’s own work, that has used a variety of methods generally finding that government health spending is not impacted under IMF programmes, or even that it increases under IMF programmes (the latter of which our findings do not support), including from its Independent Evaluation Office (van der Gaag and Barham 1998, IMF Independent Evaluation Office 2003, Martin and Segura-Ubiergo 2004, Clements et al. 2011, IMF 2017) (although with some exceptions; Thomas 2006). While academics have at times found similar results (Kentikelenis et al. 2015, Ochs 2017, Daoud and Reinsberg 2019, Boachie et al. 2022), criticisms and findings of IMF programmes and conditionalities constraining government health spending prevail on the part of academics and civil society (Ooms and Schrecker 2005, Nooruddin and Simmons 2006, Center for Global Development 2007, Stuckler and Basu 2009, Foley 2010, Kingston 2011, Stuckler and Basu 2013, Abocejo 2014, Birn et al. 2016, Stubbs et al. 2017, Brunswijck 2018, Stubbs and Kentikelenis 2018, Meurs et al. 2019, Isiani et al. 2021, Kentikelenis and Stubbs 2024).

While this debate is yet unresolved in the econometric literature, qualitative investigations into the health financing impacts of IMF conditionality are scarce (e.g. Stubbs et al. 2017). Such studies might help elucidate why findings differ, and more importantly generate learnings at the country level for which IMF policies and conditionalities help promote pooled health spending and which do not.

### Limitations

Regardless of the complexity and detail of the statistical methods employed, using observational data limits the scope for causal inference compared with using experimental data. We have sought to use the optimal econometric methods currently available for exploring our research questions with the observational data available. The system GMM estimator eliminates time-invariant confounders (fixed effects) from the first-differenced but not the levels equation, while time-variant confounders remain (Roodman 2009a, Statalist 2019). Our choices of covariates and sensitivity testing with variable swaps should have addressed the most important confounding; however, some residual confounding will remain. As an example, there are limitless different health needs that may drive both health spending but also DAH, all of which cannot be captured by any single variable. The GMM estimator partially mitigates this issue by instrumenting, addressing the issue of endogeneity, and estimating a purer and more accurate relationship between independent and dependent variables (Ullah et al. 2018).

By prioritizing valid application of the best available estimator for our data, our study was limited in its ability to inform sub-group questions, such as differences in correlation sizes between regions and income groups. This may have caused us to overlook especially regional effect modification present in other regions than SSA. We thus advise caution in making inferences at the regional level—and in particular at the country level, as for other global-level studies. In terms of delayed effects, we systematically examined lags at the 2- and 1-year level, and exploratively at deeper levels, but the presence of significant associations between variables in some model lag structure versions beyond the two-year level is possible. The variables in our main reduced form models did not turn out significant in ways that allowed for a deeper comparison of effect sizes between independent variables as envisaged. Our study was thus limited in its ability to address comparative questions such as: ‘Does aid displace government health spending more than debt servicing constrains it?’, similar to e.g. (Fosu 2007). Alternative study designs, perhaps with longer panels if omitting IMF variables, might help address this.

The best IMF conditionality data available were numerical, counting conditions (IMF Monitor 2023). This naturally overlooks the intricate nature of individual IMF conditionalities, their associated policy recommendations, and their complex and variable impacts at the country level. As noted by the publishers of the dataset, systematically capturing this level of complexity has not yet been achieved (IMF Monitor 2023), which is likely to cause an unavoidable type II error for our study, i.e. failing to identify conditionality effects, at least at the individual country level. While our study used the best available data for approaching our research questions using quantitative methods, the accompanying data limitations have motivated an in-depth, mixed-methods case study of the influence of IMF/WB conditionalities on health financing in Senegal (forthcoming).

Further minor limitations are noted in the Supplementary Online Appendix.

In summary, we believe our analysis has provided the foundation for a careful causal interpretation for some of the associations identified, with some caveats, as described in the conclusions.

## Conclusions

### Policy implications

Our study provided confirmatory evidence for the existing fungibility hypothesis of on-budget ODA^+^ for health with GHE-S, though at a low level, and added some evidence that both on- and off-budget ODA^+^ for health also appear to mildly displace or subsidize OOP, when measured per GDP. These findings should encourage EDPs in the sense that while it appears they do to a limited extent act as subsidizing agents for recipient governments, it also seems they help subsidize health service costs paid with the most inequitable source of financing in LMICs.

Providing GBS would overcome the issue of fungibility of sectoral funding disbursements at the expense of EDP control of funds. Agreements for minimal levels of government health spending could accompany GBS to ensure benefit to the health sector. Co-financing requirements are an available alternative policy tool for addressing fungibility. This should, however, ideally only be considered in adherence with the Paris and Accra principles for development cooperation, particularly the principle of country ownership (OECD 2005, 2008), and keeping in mind the broader fiscal repercussions of multiple EDPs requiring co-financing for their preferred policy area. Importantly, while modest displacement of GHE-S was only identified for on-budget ODA^+^ for health, this should not be interpreted as an argument for providing off-budget ODA^+^ for health, as this may be associated with inefficiencies from poor/absent coordination, duplication of efforts, and may undermine domestically owned development plans for the health sector.

We also provided some novel evidence that increases in public external debt servicing were associated with relative shifts in the mix of domestic health financing sources from GHE-S towards OOP, measured per CHE, however, not when measured per GDP or per capita. Our results thus point to a small compositional effect in health system payment patterns from government onto user, found to be neutral in proportion to economic output and population, but implying decreasing progressivity in the mix of domestic health financing sources with increasing public external debt servicing obligations. These findings provide modest added support to the argument for avoiding large public external burdens from a health financing perspective and should be considered by both external creditors and governments in LMICs. The argument has been made by the United Nations (United Nations Global Crisis Response Group 2023), the WB (World Bank 2023), and a number of civil society organizations and academics (e.g. Jubilee Debt Campaign 2020, Birungi et al. 2022, Development Finance International 2023, Fan and Gupta 2023, Amnesty International 2024, OXFAM 2024, The Lancet 2024), that the critical debt levels in many countries may put government health financing at risk, and resolution is needed to ensure that all public sectors, health included, can deliver their essential services. Higher debt servicing obligations can be avoided by a number of ways, including more concessional terms of lending, debt restructuring, debt cancellation, debt-to-health swaps, and more (Wuennenberg 2022, United Nations 2023). The latter works to ensure that health sectors do indeed benefit from funds freed up from debt relief.

Our main findings showed no relationship between IMF programme participation or conditionalities and GHE-S or OOP. The latter absent relationships with OOP have not been explored before this study. Only in an alternative model specification did we find evidence of IMF programme participation, not the number of IMF conditionalities, having a significant negative association with GHE-S per CHE, and not with GHE-S per GDP. Overall, these findings mostly support the interpretation that GHE-S is not affected by IMF programmes, and that OOP is not promoted either. For the IMF, the obvious policy implication is that they should focus on developing policy at the central fiscal level that supports GHE-S increases across the Global South where government health spending levels are wholly inadequate for meeting population needs, particularly in LICs (Dieleman et al. 2017, 2018, McIntyre et al. 2017, WHO 2024). The predominant IMF policy of using social spending floors seemingly does not achieve increases in GHE-S on aggregate, and other policy measures such as dedicated health spending floors, or objectives for GHE-S increases in adjustment programmes for low-spending countries should be considered, as allowed within the IMF mandate.

### Research recommendations

The economic variables investigated are subject to constant change, particularly ODA, which has seen significant recent cuts. A follow-up study investigating the same main relationships would be warranted (subject to data availability), to explore whether our findings are resilient to such changes in newer time periods.

While our study adds knowledge on associations between public external debt and domestic health financing at the global level, future studies should focus on exploring links between debt obligations and domestic health financing in individual countries. Negative effects have already been identified in some countries in SSA (Kimalu 2002, OXFAM 2021, Yiega 2022). As debt burdens grow and warnings of health financing impacts are released, a key role of researchers would be to elicit single country experiences, both showing any negative implications, but also identifying policies that have been successful at insulating health sectors from debt servicing obligations.

Similarly, while our study has explored associations between IMF programmes and conditionalities and domestic health financing sources at the aggregate, global level, future studies could unpack the complexity of IMF policy at the country level from a health financing perspective, using qualitative or mixed methods. We have one such study forthcoming from Senegal, but more country experiences are needed. Such studies might help inform which particular policies support increasing progressivity of health financing sources, and which do not, which would be helpful for informing future IMF policy making that aligns with health sectoral objectives for UHC.

## HIC authorship

This research was conducted as part of Frederik Federspiel (F.F.)’s PhD at LSHTM using secondary data from publicly available databases. Supervision and methodological input was provided by LSHTM faculty in the UK. The study was global in its scope, exploring relationships across 105 LMICs using observational data. We did not focus on any single LMIC and therefore did not seek out a research collaboration with academics based in any particular LMIC. We kindly hope for the reader’s understanding of this circumstance.

## Supplementary Material

czag070_Supplementary_Data

## Data Availability

The dataset and code used for this article can be shared on reasonable request to the corresponding author.

## Reflexivity statement

The authors are balanced in terms of gender (one male and one female), and seniority: F.F. is an early-career researcher, and J.B. is a senior researcher (professor and research leader). Both are Caucasian and currently based in Western Europe. We acknowledge that the author group is not inclusive in this regard. We refer to the HIC authorship statement. Both authors have previously spent several years working and conducting research in a number of LMICs, primarily in sub-Saharan Africa, including during parts of the research process.
