# Supplementary material for: Aid, debt, International Monetary Fund conditionalities and domestic health financing in low- and middle-income countries
Source: Health Policy Plan. 2026 May 21;41(7):1049–66. doi: 10.1093/heapol/czag070 (PMC13481784; doi:10.1093/heapol/czag070)
Supplement: Supplementary file 1 [file czag070_supplementary_data.zip › ODA_debt_IMFcond_healthfinancing_HPP_appendix_21.docx]

# Appendix: Supplementary methods description, additional results, limitations and variable metadata

## Supplementary methods description

### Variable names

| Variable | Full name |
| --- | --- |
| oop_che | Out-Of-Pocket Spending per Current Health Expenditure |
| oop_gdp | Out-Of-Pocket Spending per Gross Domestic Product |
| ghes_che | Government Health Expenditure as Source per Current Health Expenditure |
| ghes_gdp | Government Health Expenditure as Source per Gross Domestic Product |
| odaplus_health_pub_gdp | Official Development Assistance+ for health channeled via recipient country public sector per Gross Domestic Product |
| odaplus_health_civpriv_gdp | Official Development Assistance+ for health channeled via NGO’s, CSO’s and private sector per Gross Domestic Product |
| odaplus_nonhealth_pub_gdp | Official Development Assistance+ for non-health purposes channeled via recipient country public sector per Gross Domestic Product |
| odaplus_nonhealth_civpriv_gdp | Official Development Assistance+ for non-health purposes channeled via NGO’s, CSO’s and private sector per Gross Domestic Product |
| debt_serv_gdp | Debt Service per Gross Domestic Product (external Public and Publicly Guaranteed) |
| debt_stock_gdp | Debt Stock per Gross Domestic Product (external Public and Publicly Guaranteed) |
| imf_cond_ba1tot | International Monetary Fund Conditionalities Burden of Adjustment Indicator 1, Total (all policy areas) |
| imf_part | International Monetary Fund programme participation |
| gdp_cap_c | Gross Domestic Product per Capita (constant 2020 US$) |
| imr | Infant Mortality Rate |
| gov_eff | Government Effectiveness |
| corr_control | Corruption Control |
| battle_dum | Battle Dummy |
| col_indfrom_uk | Colonial Independence from United Kingdom |
| col_indfrom_fra | Colonial Independence from France |
| col_indfrom_spa | Colonial Independence from Spain |
|  | |

Please see Metadata section for further details on variables in dataset.

### Country list

Albania

Algeria

Angola

Argentina

Armenia

Azerbaijan

Bangladesh

Belarus

Belize

Benin

Bolivia

Bosnia and Herzegovina

Botswana

Brazil

Burkina Faso

Burundi

Cambodia

Cameroon

Cape Verde

Central African Republic

Chad

China

Colombia

Comoros

Congo

Costa Rica

Cote d'Ivoire

Democratic Republic of Congo

Djibouti

Dominican Republic

Ecuador

Egypt

El Salvador

Eswatini

Ethiopia

Fiji

Gabon

Gambia

Georgia

Ghana

Guatemala

Guinea

Guinea-Bissau

Guyana

Haiti

Honduras

India

Indonesia

Iran

Jamaica

Jordan

Kazakhstan

Kenya

Kyrgyz Republic

Laos

Lebanon

Lesotho

Liberia

Madagascar

Malawi

Mali

Mauritania

Mauritius

Mexico

Moldova

Mongolia

Morocco

Mozambique

Myanmar

Nepal

Nicaragua

Niger

Nigeria

North Macedonia

Pakistan

Papua New Guinea

Paraguay

Peru

Philippines

Rwanda

Samoa

Sao Tome and Principe

Senegal

Serbia

Sierra Leone

Solomon Islands

South Africa

Sri Lanka

Sudan

Tajikistan

Tanzania

Thailand

Togo

Tonga

Tunisia

Turkey

Turkmenistan

Uganda

Ukraine

Uzbekistan

Vanuatu

Vietnam

Yemen

Zambia

Zimbabwe

### Supplementary variable description

Table 1 (main manuscript) shows summary descriptive statistics for the variables used in our models.

#### Dependent variables

Our dependent variables are described and motivated in the main manuscript.

#### Independent variables of interest

Official Development Assistance Plus (ODA+) for health from the OECD CRS database included disbursements from Development Assistance Committee (DAC) countries, non-DAC countries, multilaterals and the BMGF^[1]^ due to their substantial development assistance contributions (1, 2). The categories “Health, Total” and “Population Policies/Programmes and Reproductive Health, Total” were included in this variable, while other categories were counted as non-health ODA. As Government Health Expenditure as Agent in LMICs (GHE-A, i.e. all government health spending from domestic and external revenue) ≈ GHE-S + on-budget development assistance to the health sector (10), fungibility implies a negative relationship between on-budget ODA+ for health and GHE-S, reflecting that a $1 external injection of funds into the health sector did not raise GHE-A by $1 due to the government lowering its own financing (GHE-S) (7). We deducted debt relief, administrative expenses, other in-donor country expenses and promotion of development awareness from ODA^+^ variables to count only real financial transfers with a development purpose.

#### Covariates

Our covariates are described and motivated in the main manuscript.

### Approach to serial correlation

As expected, moderate to strong serial correlation was present in our dependent variables. This is commonly seen in the literature (e.g. (Dieleman and Hanlon, 2014, Lahiani et al., 2022, Oryema et al., 2017), including for GHE-S/GDP as dependent variable (Dieleman and Hanlon, 2014)). By including lagged dependent variables, the amount of explanatory power contained in last year’s variable level is accounted for, and our independent variable estimates and error terms contain the remaining explanatory power beyond this. The use of instrumentation in System GMM is the preferred approach when faced with serial correlation in panel data series (Roodman, 2009a). The Arellano-Bond test for first-order serial correlation of the error terms in the levels equation is the guiding test in this situation (Roodman, 2009a, Arellano and Bond, 1991). We made sure that all p-values were above 0.15, including by adding 2nd-order lags where necessary, thus safely accepting the null-hypothesis that lagged GMM-style instruments were not made invalid by serial correlation of residuals (Roodman, 2009a). By not breaking up our dataset into subsets, we ensured our dataset was large enough to ensure validity of this test. Our unit root tests further firmly confirmed the absence of a unit root (below). These estimation choices and testing should thus have adequately addressed potential inference issues present when examining dependent variables that are serially correlated.

### Lag structure

Contemporaneous variable versions, with the noted exceptions, were generally found to be insignificant, or in the case of joint insignificance at both *t* and *t-1*, had lower t-values than at *t-1*. When including both unlagged and 1-year lagged independent variables of interest, strong issues of multicollinearity were encountered, degrees of freedom lost, and issues of overspecification as well as instrument proliferation from inclusion of insignificant explanatory variables substantially reduced the room for manoeuvre in the search for an appropriate model within acceptable diagnostic test parameters, in turn increasing the risk of committing type-II errors. Contemporaneous variable versions for financial independent variables of interest were therefore not included.

### Diagnostic tests

We performed twelve unit root tests for each dependent variable (48 in total, available in the appendix): 4 variations of the Fisher-type test (augmented Dickey-Fuller and Phillips-Perron), Im-Pesaran Shin (IPS) test and Levin-Lin-Chu test – with trend, demeaned with trend, and with or without Akaike Information Criteria-determined lags (Appendix). As a significant linear time trend was present in all our dependent variables, we did not perform these tests without accounting for a time trend. All 48 tests rejected the null-hypotheses that panels contained unit roots.

We tested for multicollinearity by calculating Variance Inflation Factors (VIFs) for all models (Appendix, tables A4-A8). This showed the expected collinearity of 1^st^ and 2^nd^ order lags of the dependent variable in model 3a (VIFs 17-18), moderate multicollinearity of ODA-variables in full models (VIFs 5.7-6.4), and moderate multicollinearity of $ln\left( \frac{GDP}{cap} \right)$ in full models (VIF about 5). This among other factors led us to generate the reduced form models as seen in Table 2 (main manuscript), where VIFs were less than 5 for all variables, except for the dependent variable lags in model 3a, which were necessary to convincingly pass (p>0.15) the Arellano-Bond test for serial correlation in the levels equation. This approach should have adequately addressed the issue of multicollinearity and the associated risk of Type II error, and we did find a number of significant correlations. Correlation matrices are available in the appendix.

We tested for selection bias into an IMF programme using Heckman selection models (Clements et al. 2013; Heckman 1979; Kentikelenis et al. 2015) (described in the appendix). The results of these tests were insignificant, meaning that unobserved variables that make IMF programme participation more likely were not significantly associated with our dependent health financing variables, and an Inverse Mills Ratio was therefore not included in our main regressions.

## Supplementary findings

### Results from full model regressions

Table A1: Two-step system GMM results for full model specifications

| Dependent variable | 1) OOP/CHE | 2) Ln(OOP / GDP) | 3) GHE-S / CHE | 4) Ln(GHE-S / GDP) |
| --- | --- | --- | --- | --- |
| L. dependent variable | 0.708*** [0.053] | 0.903*** [0.052] | 0.823*** [0.077] | 0.819*** [0.061] |
| L. ln(ODA^+^ for health (public) / GDP) | -0.389 [0.301] | -0.018* [0.010] | -0.311 [0.282] | -0.022** [0.011] |
| L. ln(ODA^+^ for health (civ./priv.) / GDP) | -0.456** [0.195] | -0.014* [0.007] | -0.159 [0.260] | 0.003 [0.009] |
| L. ln(ODA^+^ for non-health (public) / GDP) | 0.086 [0.337] | -0.008 [0.014] | -0.018 [0.377] | 0.004 [0.016] |
| L. ln(ODA^+^ for non-health (civ./priv.) / GDP) | 0.149 [0.533] | -0.026 [0.019] | 0.311 [0.425] | 0.019 [0.018] |
| L. ln(PPG external debt service / GDP) | 0.563* [0.292] | 0.019 [0.017] | -0.401 [0.294] | 0.004 [0.011] |
| L. ln(PPG external debt stock / GDP) | 0.176 [0.688] | -0.026 [0.021] | -0.159 [0.821] | -0.018 [0.026] |
| IMF conditionalities | -0.015 [0.012] | -0.000 [0.000] | 0.016 [0.014] | -0.000 [0.000] |
| IMF participation | 0.375 [0.462] | 0.012 [0.022] | -1.131* [0.640] | 0.006 [0.022] |
| Ln(GHE-S / GDP) | -7.530*** [1.664] | 0.063* [0.037] | - | - |
| Ln(GDPpc) | -0.077 [1.697] | -0.155** [0.070] | 2.206 [2.336] | 0.055 [0.071] |
| IMR | -0.106** [0.050] | -0.002 [0.002] | -0.015 [0.060] | -0.000 [0.002] |
| Gov. effectiveness | -0.488 [1.480] | -0.049 [0.053] | -0.961 [1.715] | 0.012 [0.053] |
| Corruption control | -1.607 [1.391] | -0.016 [0.060] | 2.603[2.210] | 0.052 [0.059] |
| Conflict | 0.559 [0.745] | 0.019 [0.023] | 0.147 [0.607] | 0.031 [0.027] |
| Colonial ind. from UK | -1.933 [1.216] | -0.015 [0.043] | -0.447 [1.286] | -0.007 [0.054] |
| Colonial ind. from France | -0.220 [1.629] | 0.031 [0.054] | -0.154 [1.069] | -0.054 [0.037] |
| Colonial ind. from Spain | -0.532 [1.480] | 0.005 [0.059] | 0.524 [1.678] | 0.016 [0.047] |
| Constant | 16.028 [14.810] | 1.161** [0.580] | -8.310 [18.187] | -0.234 [0.586] |
| Observations | 1429 | 1429 | 1429 | 1429 |
| Countries | 105 | 105 | 105 | 105 |
| Instruments | 92 | 92 | 73 | 73 |
| Lag limits (years) | 4 | 4 | 3 | 3 |
| Country fixed effects | Yes | Yes | Yes | Yes |
| Year fixed effects | Yes | Yes | Yes | Yes |
| AB-test AR(2) (p-level) | 0.996 | 0.756 | 0.155 | 0.513 |
| Hansen test (p-level) | 0.262 | 0.366 | 0.198 | 0.544 |
| Diff.-in-Hansen test (p-level) | 0.229 | 0.305 | 0.847 | 0.190 |
| F-statistic | 520.66*** | 165.84*** | 886.84*** | 260.43*** |

Windmeijer-corrected robust standard errors in brackets; *p < 0.1, **p<0.05, ***p<0.01

Civ./priv: ODA^+^ channelled via NGO’s & civil society organisations or private sector institutions.

AB-test AR(2) = Arellano-Bond test for first-order autocorrelation in the levels equation. Diff.-in-Hansen test: Difference-in-Hansen test for exogeneity of GMM instruments in levels equation. Govt.= Government. IMR= Infant Mortality Rate. Ind. = Independence (colonial). L. = 1-year lag. Ln= natural logarithm.

### Other findings for ODA variables

ODA^+^ for health purposes disbursed to the civil/private sector measured per GDP, correlated negatively with OOP/CHE in our full model (semi-elasticity: -0.456) though not in our reduced model. This association was not present at other lag-levels, it only tolerated increasing the GMM-style lag limit by 1, and it was robust to most but not all covariate swaps (below). There was a near-significant negative correlation with OOP/GDP in the full model (p=0.050), which was just significant in our reduced model (p=0.047). This effect was also present at the 2^nd^ order lag but not when unlagged, at GMM-style lag limits 5, 7 and 8, and with all covariate swaps. We found no association with GHE-S/CHE or with GHE-S/GDP.

We also found a significant negative association between ODA^+^ for non-health purposes channelled via the civil/private sector and OOP/GDP in our reduced model (elasticity: -0.04), but not our full model. This finding was not robust to changes in the lag limit. The effect was also present in a 2^nd^ order lag version of our full model but not of the reduced model, and not in a contemporaneous version. It was robust to all covariate swaps.

#### Covariate findings

We made the following findings among our included covariates:

A 1% increase in GHES/GDP was associated with a -0.08 percentage point decrease in OOP/CHE, both in full ([-0.11: -0.04], p=0.000) and reduced ([-0.11: -0.05], p=0.000) models. This association remained significant when chancing variable lag-structures, GMM-style instrument lag limits (below) and covariates. A 1% increase in GHES/GDP was also associated with a 0.07% ([0.001: 0.14], p=0.048) increase in OOP/GDP in the reduced model, which was also robust to the same changes.

We found a significant negative association between GDP per capita and OOP/GDP (full model: elasticity: -0.15 ([-0.29: -0.02], p=0.02); reduced model: elasticity -0.25 ([-0.40: -0.11], p=0.001). This finding only remained in reduced model versions with 2^nd^ order lags of the independent variables of interest, but not other variable lag variations. It was however robust to changes in GMM-style lag limits and to many but not all covariate swaps.

One additional infant dying before reaching one year of age per 1,000 live births was associated with a -0.1 ([-0.20: -0.008], p=0.038) percentage point decrease in OOP/CHE in our full model, but this was insignificant in our reduced model (p=0.058). The finding was not robust to changes in lag-levels of independent variables of interest, but it was robust to changing GMM lag limits and to some, but not all covariate swaps.

A one standard-deviation increase in the level of control of corruption as measured in the Worldwide Governance Indicators (40) was associated with a 3.2 ([0.61: 5.75], p=0.017) percentage point increase in GHES/CHE in our reduced model. This finding was robust when independent variables of interest were lagged two years, but not when they were contemporaneous. The finding remained when increasing, but not decreasing lag limits, and it was robust to swapping mortality indicators but not colonial history covariates.

### Further sensitivity testing

#### Interaction terms

None of the tested interaction terms had any robustly significant effects on any of our relationships of interest within acceptable model test parameters (Appendix).

#### Alternative variable specifications

When measuring our financial variables per capita instead of per GDP, including dependent variables, we found some evidence of crowding out/displacement from other sectors benefiting the health sector: lagged ODA^+^ for non-health purposes channelled via the public sector per capita had a positive association with GHE-S per capita (semi-elasticity: -0.0004, p=0.019) (full model). Similar to our main findings, lagged debt service per capita had a negative association with GHE-S/CHE (p=0.02). Our findings for ODA^+^ for health via the public sector were not present when specifying per capita.

#### No log-transformation

When measuring our variables directly and not log-transformed, including dependent variables, this meant that we deviated from much of the literature in our field and introduced substantial right-skew of many variables into the models. The findings with this specification deviated substantially from our main model findings. We confirmed that no unit root was present for direct dependent variables. We were unable to arrive at a specification for model 1 that convincingly passed diagnostic tests without overspecification. We again found a significant negative correlation between ODA^+^ for health channelled via the public sector per GDP and OOP/GDP in both reduced and full models. We also found a new positive association between IMF programme participation two years prior and OOP/GDP (0.17 %-points, p=0.001). We found a new significant negative association between lagged PPG external debt stock per GDP and GHE-S/CHE (-0.06 %-points, p=0.015), and a new positive association between ODA^+^ for health per GDP channelled via the civil/private sector and GHE-S/GDP (0.17 %-points, p=0.037).

#### Alternative deflation data

Using US GDP deflator data instead of US consumer price index data for deflation of per-capita variables had no significant effect on any of our results (World Bank, 2025).

#### Aggregating ODA-variables

When aggregating our logged ODA-variables into just health and non-health purposes irrespective of channel of assistance, the “fungibility effect” of a decrease in GHE-S/GDP from an increase in lagged ODA^+^ for health per GDP was not found again, while the negative association with OOP/GDP remained (p=0.01) (Appendix). Again, the lag of debt service per GDP was positively associated with OOP/CHE (p=0.04), but no longer with a decrease in GHE-S/CHE.

#### Adding Inverse Mills Ratios

Finally, adding Inverse Mills Ratios to our full models did not cause any substantive changes to our overall results or conclusions.

Apart from when changing the variable being measured (per capita, not log-transformed, aggregated etc.), effect sizes did not change substantially in sensitivity tests. (*Some changes in coefficients and p-values were seen when including Inverse Mills Ratios, which was most likely due to the loss of degrees of freedom resulting from the inclusion of new explanatory variables with incomplete data for our Heckman selection model and the following need to adjust some model lag limits to avoid overspecification (see Appendix)*).

## Diagnostic test results

### Correlation matrices

Table A2: Pairwise correlations, significance level p<0.05

| Variables | (1) | (2) | (3) | (4) | (5) | (6) | (7) | (8) | (9) | (10) | (11) | (12) | (13) | (14) | (15) | (16) | (17) | (18) | (19) | (20) |
| --- | --- | --- | --- | --- | --- | --- | --- | --- | --- | --- | --- | --- | --- | --- | --- | --- | --- | --- | --- | --- |
| (1) oop_che | 1.00 |  |  |  |  |  |  |  |  |  |  |  |  |  |  |  |  |  |  |  |
| (2) oop_gdp | -0.65* | 1.00 |  |  |  |  |  |  |  |  |  |  |  |  |  |  |  |  |  |  |
| (3) ghes_che | 0.78* | -0.46* | 1.00 |  |  |  |  |  |  |  |  |  |  |  |  |  |  |  |  |  |
| (4) ghes_gdp | -0.56* | 0.82* | -0.13* | 1.00 |  |  |  |  |  |  |  |  |  |  |  |  |  |  |  |  |
| (5) ln_odaplus_health_pub_gdp | -0.10* | -0.39* | -0.17* | -0.32* | 1.00 |  |  |  |  |  |  |  |  |  |  |  |  |  |  |  |
| (6) ln_odaplus_health_civpriv_gdp | -0.06* | -0.47* | -0.08* | -0.35* | 0.82* | 1.00 |  |  |  |  |  |  |  |  |  |  |  |  |  |  |
| (7) ln_odaplus_nonhealth_pub_gdp | -0.07* | -0.29* | -0.08* | -0.21* | 0.81* | 0.70* | 1.00 |  |  |  |  |  |  |  |  |  |  |  |  |  |
| (8) ln_odaplus_nonhealth_civpriv_gdp | -0.03 | -0.38* | 0.01 | -0.25* | 0.77* | 0.79* | 0.82* | 1.00 |  |  |  |  |  |  |  |  |  |  |  |  |
| (9) ln_debt_serv_gdp | -0.08* | 0.29* | 0.02 | 0.29* | -0.18* | -0.20* | -0.05 | -0.11* | 1.00 |  |  |  |  |  |  |  |  |  |  |  |
| (10) ln_debt_stock_gdp | 0.00 | -0.11* | 0.06* | -0.05* | 0.36* | 0.32* | 0.47* | 0.48* | 0.54* | 1.00 |  |  |  |  |  |  |  |  |  |  |
| (11) imf_cond_ba1tot | 0.09* | -0.22* | 0.15* | -0.12* | 0.25* | 0.23* | 0.30* | 0.29* | 0.01 | 0.17* | 1.00 |  |  |  |  |  |  |  |  |  |
| (12) imf_part | 0.10* | -0.26* | 0.17* | -0.13* | 0.29* | 0.28* | 0.37* | 0.34* | 0.01 | 0.20* | 0.84* | 1.00 |  |  |  |  |  |  |  |  |
| (13) gdp_cap_c | -0.22* | 0.68* | -0.13* | 0.58* | -0.69* | -0.70* | -0.59* | -0.68* | 0.31* | -0.27* | -0.27* | -0.31* | 1.00 |  |  |  |  |  |  |  |
| (14) imr | 0.24* | -0.64* | 0.04 | -0.66* | 0.49* | 0.52* | 0.31* | 0.40* | -0.33* | 0.08* | 0.22* | 0.23* | -0.72* | 1.00 |  |  |  |  |  |  |
| (15) gov_eff | -0.33* | 0.56* | -0.19* | 0.56* | -0.36* | -0.46* | -0.26* | -0.45* | 0.29* | -0.10* | -0.16* | -0.19* | 0.60* | -0.61* | 1.00 |  |  |  |  |  |
| (16) corr_control | -0.49* | 0.51* | -0.33* | 0.53* | -0.08* | -0.18* | 0.03 | -0.16* | 0.19* | -0.01 | -0.09* | -0.07* | 0.40* | -0.41* | 0.74* | 1.00 |  |  |  |  |
| (17) battle_dum | 0.19* | -0.13* | 0.08* | -0.18* | -0.11* | -0.05* | -0.07* | 0.03 | -0.06* | -0.09* | 0.02 | 0.03 | -0.13* | 0.17* | -0.13* | -0.19* | 1.00 |  |  |  |
| (18) col_indfrom_uk | -0.21* | 0.00 | -0.27* | 0.00 | 0.23* | 0.18* | 0.05* | 0.06* | -0.06* | -0.02 | -0.07* | -0.08* | -0.05 | 0.12* | 0.12* | 0.18* | -0.01 | 1.00 |  |  |
| (19) col_indfrom_fra | 0.16* | -0.27* | 0.01 | -0.36* | 0.17* | 0.15* | 0.23* | 0.18* | -0.11* | 0.08* | 0.14* | 0.16* | -0.24* | 0.38* | -0.34* | -0.23* | 0.04 | -0.31* | 1.00 |  |
| (20) col_indfrom_spa | -0.08* | 0.21* | 0.05 | 0.23* | -0.32* | -0.22* | -0.27* | -0.20* | 0.11* | -0.06* | -0.10* | -0.11* | 0.26* | -0.19* | 0.12* | 0.03 | 0.00 | -0.14* | -0.13* | 1.00 |
|  | | | | | | | | | | | | | | | | | | | | |

Table A3: Pairwise correlations, significance level p<0.001

| Variables | (1) | (2) | (3) | (4) | (5) | (6) | (7) | (8) | (9) | (10) | (11) | (12) | (13) | (14) | (15) | (16) | (17) | (18) | (19) | (20) |
| --- | --- | --- | --- | --- | --- | --- | --- | --- | --- | --- | --- | --- | --- | --- | --- | --- | --- | --- | --- | --- |
| (1) oop_che | 1.00 |  |  |  |  |  |  |  |  |  |  |  |  |  |  |  |  |  |  |  |
| (2) oop_gdp | -0.65* | 1.00 |  |  |  |  |  |  |  |  |  |  |  |  |  |  |  |  |  |  |
| (3) ghes_che | 0.78* | -0.46* | 1.00 |  |  |  |  |  |  |  |  |  |  |  |  |  |  |  |  |  |
| (4) ghes_gdp | -0.56* | 0.82* | -0.13* | 1.00 |  |  |  |  |  |  |  |  |  |  |  |  |  |  |  |  |
| (5) ln_odaplus_health_pub_gdp | -0.10* | -0.39* | -0.17* | -0.32* | 1.00 |  |  |  |  |  |  |  |  |  |  |  |  |  |  |  |
| (6) ln_odaplus_health_civpriv_gdp | -0.06 | -0.47* | -0.08 | -0.35* | 0.82* | 1.00 |  |  |  |  |  |  |  |  |  |  |  |  |  |  |
| (7) ln_odaplus_nonhealth_pub_gdp | -0.07 | -0.29* | -0.08 | -0.21* | 0.81* | 0.70* | 1.00 |  |  |  |  |  |  |  |  |  |  |  |  |  |
| (8) ln_odaplus_nonhealth_civpriv_gdp | -0.03 | -0.38* | 0.01 | -0.25* | 0.77* | 0.79* | 0.82* | 1.00 |  |  |  |  |  |  |  |  |  |  |  |  |
| (9) ln_debt_serv_gdp | -0.08 | 0.29* | 0.02 | 0.29* | -0.18* | -0.20* | -0.05 | -0.11* | 1.00 |  |  |  |  |  |  |  |  |  |  |  |
| (10) ln_debt_stock_gdp | 0.00 | -0.11* | 0.06 | -0.05 | 0.36* | 0.32* | 0.47* | 0.48* | 0.54* | 1.00 |  |  |  |  |  |  |  |  |  |  |
| (11) imf_cond_ba1tot | 0.09* | -0.22* | 0.15* | -0.12* | 0.25* | 0.23* | 0.30* | 0.29* | 0.01 | 0.17* | 1.00 |  |  |  |  |  |  |  |  |  |
| (12) imf_part | 0.10* | -0.26* | 0.17* | -0.13* | 0.29* | 0.28* | 0.37* | 0.34* | 0.01 | 0.20* | 0.84* | 1.00 |  |  |  |  |  |  |  |  |
| (13) gdp_cap_c | -0.22* | 0.68* | -0.13* | 0.58* | -0.69* | -0.70* | -0.59* | -0.68* | 0.31* | -0.27* | -0.27* | -0.31* | 1.00 |  |  |  |  |  |  |  |
| (14) imr | 0.24* | -0.64* | 0.04 | -0.66* | 0.49* | 0.52* | 0.31* | 0.40* | -0.33* | 0.08 | 0.22* | 0.23* | -0.72* | 1.00 |  |  |  |  |  |  |
| (15) gov_eff | -0.33* | 0.56* | -0.19* | 0.56* | -0.36* | -0.46* | -0.26* | -0.45* | 0.29* | -0.10* | -0.16* | -0.19* | 0.60* | -0.61* | 1.00 |  |  |  |  |  |
| (16) corr_control | -0.49* | 0.51* | -0.33* | 0.53* | -0.08 | -0.18* | 0.03 | -0.16* | 0.19* | -0.01 | -0.09* | -0.07 | 0.40* | -0.41* | 0.74* | 1.00 |  |  |  |  |
| (17) battle_dum | 0.19* | -0.13* | 0.08 | -0.18* | -0.11* | -0.05 | -0.07 | 0.03 | -0.06 | -0.09* | 0.02 | 0.03 | -0.13* | 0.17* | -0.13* | -0.19* | 1.00 |  |  |  |
| (18) col_indfrom_uk | -0.21* | 0.00 | -0.27* | 0.00 | 0.23* | 0.18* | 0.05 | 0.06 | -0.06 | -0.02 | -0.07 | -0.08 | -0.05 | 0.12* | 0.12* | 0.18* | -0.01 | 1.00 |  |  |
| (19) col_indfrom_fra | 0.16* | -0.27* | 0.01 | -0.36* | 0.17* | 0.15* | 0.23* | 0.18* | -0.11* | 0.08 | 0.14* | 0.16* | -0.24* | 0.38* | -0.34* | -0.23* | 0.04 | -0.31* | 1.00 |  |
| (20) col_indfrom_spa | -0.08 | 0.21* | 0.05 | 0.23* | -0.32* | -0.22* | -0.27* | -0.20* | 0.11* | -0.06 | -0.10* | -0.11* | 0.26* | -0.19* | 0.12* | 0.03 | 0.00 | -0.14* | -0.13* | 1.00 |
|  | | | | | | | | | | | | | | | | | | | | |

### Variance Inflation Factors for multicollinearity

The below tables show variables with moderate/high degrees of multicollinearity, having Variance Inflation Factors (VIF) above 5, for all models:

Table A4: Model 1)

| Variable | VIF |
| --- | --- |
| L. ln_odaplus_nonhealth_pub_gdp | 6.42 |
| L. ln_odaplus_health_pub_gdp | 6.01 |
| L. ln_odaplus_nonhealth_civpriv_gdp | 5.86 |
| ln_gdp_cap_c | 5.04 |

Table A5: Model 2)

| Variable | VIF |
| --- | --- |
| L. ln_odaplus_nonhealth_pub_gdp | 6.40 |
| L. ln_odaplus_health_pub_gdp | 6.11 |
| L. ln_odaplus_nonhealth_civpriv_gdp | 5.88 |
| ln_gdp_cap_c | 5.17 |

Table A6: Model 3)

| Variable | VIF |
| --- | --- |
| L. ln_odaplus_nonhealth_pub_gdp | 6.40 |
| L. ln_odaplus_health_pub_gdp | 5.88 |
| L. ln_odaplus_nonhealth_civpriv_gdp | 5.72 |
| ln_gdp_cap_c | 5.42 |

Table A7: Model 4)

| Variable | VIF |
| --- | --- |
| L. ln_odaplus_nonhealth_pub_gdp | 6.40 |
| L. ln_odaplus_nonhealth_civpriv_gdp | 5.86 |
| L. ln_odaplus_health_pub_gdp | 5.85 |
| ln_gdp_cap_c | 5.03 |

**Model 1a)**

None

**Model 2a)**

None

Table A8: Model 3a)

| Variable | VIF |
| --- | --- |
| L. ghes_che | 17.84 |
| L2. ghes_che | 17.37 |

**Model 4a)**

None

### Unit root testing of dependent variables

AIC= Akaike Information Criterion. Null hypothesis: Panels contain unit root. For Im-Pesaran Shin unit-root test: T-bar needs to have a higher absolute value than cut-off to reject null.

Table A9: OOP/CHE

| Test type | ADF lags | Trend? | Demean? | Test statistic | Value | 5% alpha-level cutoff |
| --- | --- | --- | --- | --- | --- | --- |
| Fisher-type unit-root test (Augmented Dickey-Fuller) | 1 | Yes | No | Inverse chi-squared (p-value) | 0.0000 |  |
| Fisher-type unit-root test (Augmented Dickey-Fuller) | 1 | Yes | Yes | Inverse chi-squared (p-value) | 0.0000 |  |
| Fisher-type unit-root test (Phillips-Perron) | 1 | Yes | No | Inverse chi-squared (p-value) | 0.0000 |  |
| Fisher-type unit-root test (Phillips-Perron) | 1 | Yes | Yes | Inverse chi-squared (p-value) | 0.0000 |  |
| Im-Pesaran-Shin unit-root test | 0 | Yes | No | T-bar | -2.3920 | -2.320 |
| Im-Pesaran-Shin unit-root test | 0 | Yes | No | Z-t-tilde-bar (p-value) | 0.0000 |  |
| Im-Pesaran-Shin unit-root test | 0 | Yes | Yes | T-bar | -2.3511 | -2.320 |
| Im-Pesaran-Shin unit-root test | 0 | Yes | Yes | Z-t-tilde-bar (p-value) | 0.0000 |  |
| Im-Pesaran-Shin unit-root test | 2.00 (chosen by AIC) | Yes | No | W-t-bar (p-value) | 0.0000 |  |
| Im-Pesaran-Shin unit-root test | 2.07 (chosen by AIC) | Yes | Yes | W-t-bar (p-value) | 0.0000 |  |
| Levin-Lin-Chu | 1 | Yes | No | Adjusted t (p-value) | 0.0000 |  |
| Levin-Lin-Chu | 1 | Yes | Yes | Adjusted t (p-value) | 0.0000 |  |
| Levin-Lin-Chu | 2.00 (chosen by AIC) | Yes | No | Adjusted t (p-value) | 0.0000 |  |
| Levin-Lin-Chu | 2.07 (chosen by AIC) | Yes | Yes | Adjusted t (p-value) | 0.0000 |  |

Table A10: Ln(OOP/GDP)

| Test type | Lags | Trend? | Demean? | Test statistic | Value | 5% alpha-level cutoff |
| --- | --- | --- | --- | --- | --- | --- |
| Fisher-type unit-root test (Augmented Dickey-Fuller) | 1 | Yes | No | Inverse chi-squared (p-value) | 0.0000 |  |
| Fisher-type unit-root test (Augmented Dickey-Fuller) | 1 | Yes | Yes | Inverse chi-squared (p-value) | 0.0000 |  |
| Fisher-type unit-root test (Phillips-Perron) | 1 | Yes | No | Inverse chi-squared (p-value) | 0.0000 |  |
| Fisher-type unit-root test (Phillips-Perron) | 1 | Yes | Yes | Inverse chi-squared (p-value) | 0.0000 |  |
| Im-Pesaran-Shin unit-root test | 0 | Yes | No | T-bar | -2.3856 | -2.320 |
| Im-Pesaran-Shin unit-root test | 0 | Yes | No | Z-t-tilde-bar (p-value) | 0.0000 |  |
| Im-Pesaran-Shin unit-root test | 0 | Yes | Yes | T-bar | -2.3941 | -2.320 |
| Im-Pesaran-Shin unit-root test | 0 | Yes | Yes | Z-t-tilde-bar (p-value) | 0.0000 |  |
| Im-Pesaran-Shin unit-root test | 2.13 (chosen by AIC) | Yes | No | W-t-bar (p-value) | 0.0004 |  |
| Im-Pesaran-Shin unit-root test | 2.21 (chosen by AIC) | Yes | Yes | W-t-bar (p-value) | 0.0000 |  |
| Levin-Lin-Chu | 1 | Yes | No | Adjusted t (p-value) | 0.0000 |  |
| Levin-Lin-Chu | 1 | Yes | Yes | Adjusted t (p-value) | 0.0000 |  |
| Levin-Lin-Chu | 2.13 (chosen by AIC) | Yes | No | Adjusted t (p-value) | 0.0223 |  |
| Levin-Lin-Chu | 1.21 (chosen by AIC) | Yes | Yes | Adjusted t (p-value) | 0.0000 |  |

Table A11: GHES/CHE

| Test type | Lags | Trend? | Demean? | Test statistic | Value | 5% alpha-level cutoff |
| --- | --- | --- | --- | --- | --- | --- |
| Fisher-type unit-root test (Augmented Dickey-Fuller) | 1 | Yes | No | Inverse chi-squared (p-value) | 0.0000 |  |
| Fisher-type unit-root test (Augmented Dickey-Fuller) | 1 | Yes | Yes | Inverse chi-squared (p-value) | 0.0000 |  |
| Fisher-type unit-root test (Phillips-Perron) | 1 | Yes | No | Inverse chi-squared (p-value) | 0.0000 |  |
| Fisher-type unit-root test (Phillips-Perron) | 1 | Yes | Yes | Inverse chi-squared (p-value) | 0.0000 |  |
| Im-Pesaran-Shin unit-root test | 0 | Yes | No | T-bar | -2.5662 | -2.320 |
| Im-Pesaran-Shin unit-root test | 0 | Yes | No | Z-t-tilde-bar (p-value) | 0.0000 |  |
| Im-Pesaran-Shin unit-root test | 0 | Yes | Yes | T-bar | -2.5712 | -2.320 |
| Im-Pesaran-Shin unit-root test | 0 | Yes | Yes | Z-t-tilde-bar (p-value) | 0.0000 |  |
| Im-Pesaran-Shin unit-root test | 1.78 (chosen by AIC) | Yes | No | W-t-bar (p-value) | 0.0247 |  |
| Im-Pesaran-Shin unit-root test | 1.85 (chosen by AIC) | Yes | Yes | W-t-bar (p-value) | 0.0019 |  |
| Levin-Lin-Chu | 1 | Yes | No | Adjusted t (p-value) | 0.0000 |  |
| Levin-Lin-Chu | 1 | Yes | Yes | Adjusted t (p-value) | 0.0000 |  |
| Levin-Lin-Chu | 1.78 (chosen by AIC) | Yes | No | Adjusted t (p-value) | 0.0000 |  |
| Levin-Lin-Chu | 1.85 (chosen by AIC) | Yes | Yes | Adjusted t (p-value) | 0.0000 |  |

Table A12: Ln(GHES/GDP)

| Test type | Lags | Trend? | Demean? | Test statistic | Value | 5% alpha-level cutoff |
| --- | --- | --- | --- | --- | --- | --- |
| Fisher-type unit-root test (Augmented Dickey-Fuller) | 1 | Yes | No | Inverse chi-squared (p-value) | 0.0000 |  |
| Fisher-type unit-root test (Augmented Dickey-Fuller) | 1 | Yes | Yes | Inverse chi-squared (p-value) | 0.0000 |  |
| Fisher-type unit-root test (Phillips-Perron) | 1 | Yes | No | Inverse chi-squared (p-value) | 0.0000 |  |
| Fisher-type unit-root test (Phillips-Perron) | 1 | Yes | Yes | Inverse chi-squared (p-value) | 0.0000 |  |
| Im-Pesaran-Shin unit-root test | 0 | Yes | No | T-bar | -2.4913 | -2.320 |
| Im-Pesaran-Shin unit-root test | 0 | Yes | No | Z-t-tilde-bar (p-value) | 0.0000 |  |
| Im-Pesaran-Shin unit-root test | 0 | Yes | Yes | T-bar | -2.5598 | -2.320 |
| Im-Pesaran-Shin unit-root test | 0 | Yes | Yes | Z-t-tilde-bar (p-value) | 0.0000 |  |
| Im-Pesaran-Shin unit-root test | 1.98 (chosen by AIC) | Yes | No | W-t-bar (p-value) | 0.0000 |  |
| Im-Pesaran-Shin unit-root test | 2.25 (chosen by AIC) | Yes | Yes | W-t-bar (p-value) | 0.0000 |  |
| Levin-Lin-Chu | 1 | Yes | No | Adjusted t (p-value) | 0.0000 |  |
| Levin-Lin-Chu | 1 | Yes | Yes | Adjusted t (p-value) | 0.0000 |  |
| Levin-Lin-Chu | 1.98 (chosen by AIC) | Yes | No | Adjusted t (p-value) | 0.0000 |  |
| Levin-Lin-Chu | 2.25 (chosen by AIC) | Yes | Yes | Adjusted t (p-value) | 0.0000 |  |

## Sensitivity analysis

### Alternative lag-specifications

*(Year dummies were included in all models but not presented here for brevity. GMM-style instrument generation limited to 5-year lags to show isolated effect of changing lag structures).*

**No lags on independent variables of interest:**

- Full models:

Table A13: Model 1

| oop_che | Coef. | | St.Err. | t-value | | p-value | [95% Conf | | Interval] | | Sig |
| --- | --- | --- | --- | --- | --- | --- | --- | --- | --- | --- | --- |
| L. oop_che | .72 | | .06 | 13.12 | | 0 | .61 | | .83 | | *** |
| ln_odaplus_health_pub_gdp | -.27 | | .29 | -0.93 | | .35 | -.83 | | .3 | |  |
| ln_odaplus_health_civpriv_gdp | -.37 | | .21 | -1.80 | | .08 | -.78 | | .04 | | * |
| ln_odaplus_nonhealth_pub_gdp | .53 | | .55 | 0.97 | | .33 | -.55 | | 1.62 | |  |
| ln_odaplus_nonhealth_civpriv_gdp | .11 | | .37 | 0.28 | | .78 | -.64 | | .85 | |  |
| ln_debt_serv_gdp | -.18 | | .54 | -0.34 | | .74 | -1.26 | | .9 | |  |
| ln_debt_stock_gdp | .19 | | .78 | 0.25 | | .8 | -1.36 | | 1.74 | |  |
| imf_cond_ba1tot | -.01 | | .01 | -0.57 | | .57 | -.03 | | .02 | |  |
| imf_part | .43 | | .54 | 0.80 | | .43 | -.64 | | 1.49 | |  |
| ln_ghes_gdp | -6.94 | | 1.49 | -4.67 | | 0 | -9.89 | | -3.99 | | *** |
| ln_gdp_cap_c | -.19 | | 2.09 | -0.09 | | .93 | -4.34 | | 3.95 | |  |
| imr | -.11 | | .06 | -1.91 | | .06 | -.23 | | 0 | | * |
| gov_eff | -.39 | | 1.76 | -0.22 | | .83 | -3.88 | | 3.1 | |  |
| corr_control | -1.19 | | 2.27 | -0.53 | | .6 | -5.7 | | 3.31 | |  |
| battle_dum | .84 | | .83 | 1.00 | | .32 | -.82 | | 2.49 | |  |
| col_indfrom_uk | -1.99 | | 1.68 | -1.18 | | .24 | -5.33 | | 1.35 | |  |
| col_indfrom_fra | -.87 | | 1.72 | -0.51 | | .61 | -4.29 | | 2.55 | |  |
| col_indfrom_spa | .34 | | 1.36 | 0.25 | | .8 | -2.37 | | 3.05 | |  |
| Mean dependent var | | 40.102 | | | SD dependent var | | | 19.365 | |  |  |
| Number of obs | | 1443 | | | F-test | | | 766.482 | |  |  |
| **** p<.01, ** p<.05, * p<.1* | | | | | | | | | | | |
|  | | | | | | | | | | | |

Countries: 105

Instrument count: 92

Lag limit (years): 4

AB test (AR2) (p-value): 0.916

Hansen test (p-value): 0.220

Difference-in-Hansen test (p-value): 0.525

Table A14: Model 2

| ln_oop_gdp | Coef. | | St.Err. | t-value | | p-value | [95% Conf | | Interval] | | Sig |
| --- | --- | --- | --- | --- | --- | --- | --- | --- | --- | --- | --- |
| L. ln_oop_gdp | .94 | | .05 | 17.69 | | 0 | .83 | | 1.04 | | *** |
| ln_odaplus_health_pub_gdp | -.01 | | .01 | -1.29 | | .2 | -.03 | | .01 | |  |
| ln_odaplus_health_civpriv_gdp | -.01 | | .01 | -1.21 | | .23 | -.03 | | .01 | |  |
| ln_odaplus_nonhealth_pub_gdp | .01 | | .02 | 0.33 | | .74 | -.03 | | .05 | |  |
| ln_odaplus_nonhealth_civpriv_gdp | .01 | | .01 | 0.59 | | .56 | -.02 | | .04 | |  |
| ln_debt_serv_gdp | -.01 | | .01 | -0.86 | | .39 | -.03 | | .01 | |  |
| ln_debt_stock_gdp | .03 | | .03 | 0.92 | | .36 | -.03 | | .08 | |  |
| imf_cond_ba1tot | 0 | | 0 | -0.75 | | .46 | 0 | | 0 | |  |
| imf_part | .01 | | .02 | 0.43 | | .67 | -.03 | | .05 | |  |
| ln_ghes_gdp | .07 | | .04 | 1.71 | | .09 | -.01 | | .14 | | * |
| ln_gdp_cap_c | -.07 | | .07 | -1.00 | | .32 | -.19 | | .06 | |  |
| imr | 0 | | 0 | 0.13 | | .9 | 0 | | 0 | |  |
| gov_eff | 0 | | .05 | 0.10 | | .92 | -.09 | | .1 | |  |
| corr_control | -.02 | | .05 | -0.45 | | .65 | -.12 | | .08 | |  |
| battle_dum | .03 | | .02 | 1.66 | | .1 | -.01 | | .07 | | * |
| col_indfrom_uk | -.03 | | .04 | -0.72 | | .47 | -.11 | | .05 | |  |
| col_indfrom_fra | -.01 | | .05 | -0.14 | | .89 | -.1 | | .08 | |  |
| col_indfrom_spa | 0 | | .06 | 0.05 | | .96 | -.12 | | .13 | |  |
| Mean dependent var | | 0.550 | | | SD dependent var | | | 0.733 | |  |  |
| Number of obs | | 1443 | | | F-test | | | 261.474 | |  |  |
| **** p<.01, ** p<.05, * p<.1* | | | | | | | | | | | |
|  | | | | | | | | | | | |

Countries: 105

Instrument count: 92

Lag limit (years): 4

AB test (AR2) (p-value): 0.458

Hansen test (p-value): 0.275

Difference-in-Hansen test (p-value): 0.170

Table A15: Model 3

| ghes_che | Coef. | | St.Err. | t-value | | p-value | [95% Conf | | Interval] | | Sig |
| --- | --- | --- | --- | --- | --- | --- | --- | --- | --- | --- | --- |
| L. ghes_che | .83 | | .08 | 9.84 | | 0 | .66 | | .99 | | *** |
| ln_odaplus_health_pub_gdp | .03 | | .25 | 0.11 | | .92 | -.48 | | .53 | |  |
| ln_odaplus_health_civpriv_gdp | -.16 | | .19 | -0.82 | | .41 | -.54 | | .22 | |  |
| ln_odaplus_nonhealth_pub_gdp | -.09 | | .56 | -0.17 | | .87 | -1.21 | | 1.02 | |  |
| ln_odaplus_nonhealth_civpriv_gdp | .6 | | .5 | 1.19 | | .24 | -.4 | | 1.6 | |  |
| ln_debt_serv_gdp | .28 | | .3 | 0.93 | | .35 | -.32 | | .88 | |  |
| ln_debt_stock_gdp | -.45 | | .78 | -0.58 | | .56 | -1.99 | | 1.09 | |  |
| imf_cond_ba1tot | .01 | | .01 | 1.15 | | .25 | -.01 | | .04 | |  |
| imf_part | -1.13 | | .51 | -2.21 | | .03 | -2.14 | | -.12 | | ** |
| ln_gdp_cap_c | 2.23 | | 2.4 | 0.93 | | .36 | -2.53 | | 7 | |  |
| imr | -.05 | | .07 | -0.73 | | .47 | -.19 | | .09 | |  |
| gov_eff | -2.29 | | 1.5 | -1.53 | | .13 | -5.27 | | .68 | |  |
| corr_control | 2.52 | | 1.85 | 1.36 | | .18 | -1.15 | | 6.2 | |  |
| battle_dum | .21 | | .58 | 0.37 | | .71 | -.93 | | 1.35 | |  |
| col_indfrom_uk | .72 | | 1.18 | 0.61 | | .54 | -1.61 | | 3.06 | |  |
| col_indfrom_fra | .24 | | 1.03 | 0.23 | | .82 | -1.8 | | 2.28 | |  |
| col_indfrom_spa | 1.61 | | 1.86 | 0.86 | | .39 | -2.09 | | 5.3 | |  |
| Mean dependent var | | 40.225 | | | SD dependent var | | | 19.033 | |  |  |
| Number of obs | | 1443 | | | F-test | | | 985.136 | |  |  |
| **** p<.01, ** p<.05, * p<.1* | | | | | | | | | | | |
|  | | | | | | | | | | | |

Countries: 105

Instrument count: 73

Lag limit (years): 3

AB test (AR2) (p-value): 0.192

Hansen test (p-value): 0.430

Difference-in-Hansen test (p-value): 0.768

Table A16: Model 4

| ln_ghes_gdp | Coef. | | St.Err. | t-value | | p-value | [95% Conf | | Interval] | | Sig |
| --- | --- | --- | --- | --- | --- | --- | --- | --- | --- | --- | --- |
| L. ghes_dp | .82 | | .07 | 12.32 | | 0 | .69 | | .95 | | *** |
| ln_odaplus_health_pub_gdp | .01 | | .01 | 1.35 | | .18 | 0 | | .02 | |  |
| ln_odaplus_health_civpriv_gdp | 0 | | .01 | 0.03 | | .97 | -.02 | | .02 | |  |
| ln_odaplus_nonhealth_pub_gdp | 0 | | .02 | -0.09 | | .93 | -.04 | | .03 | |  |
| ln_odaplus_nonhealth_civpriv_gdp | .01 | | .02 | 0.75 | | .45 | -.02 | | .05 | |  |
| ln_debt_serv_gdp | .02 | | .01 | 1.60 | | .11 | 0 | | .04 | |  |
| ln_debt_stock_gdp | 0 | | .03 | -0.17 | | .87 | -.06 | | .05 | |  |
| imf_cond_ba1tot | 0 | | 0 | 0.02 | | .98 | 0 | | 0 | |  |
| imf_part | -.01 | | .02 | -0.56 | | .58 | -.05 | | .03 | |  |
| ln_gdp_cap_c | .09 | | .07 | 1.20 | | .23 | -.06 | | .24 | |  |
| imr | 0 | | 0 | -1.18 | | .24 | -.01 | | 0 | |  |
| gov_eff | -.04 | | .05 | -0.83 | | .41 | -.14 | | .06 | |  |
| corr_control | .01 | | .06 | 0.21 | | .83 | -.11 | | .14 | |  |
| battle_dum | .03 | | .03 | 1.26 | | .21 | -.02 | | .09 | |  |
| col_indfrom_uk | -.01 | | .04 | -0.16 | | .87 | -.09 | | .07 | |  |
| col_indfrom_fra | -.04 | | .04 | -0.92 | | .36 | -.13 | | .05 | |  |
| col_indfrom_spa | .03 | | .04 | 0.69 | | .49 | -.05 | | .11 | |  |
| Mean dependent var | | 0.560 | | | SD dependent var | | | 0.721 | |  |  |
| Number of obs | | 1443 | | | F-test | | | 377.266 | |  |  |
| **** p<.01, ** p<.05, * p<.1* | | | | | | | | | | | |
|  | | | | | | | | | | | |

Countries: 105

Instrument count: 73

Lag limit (years): 3

AB test (AR2) (p-value): 0.458

Hansen test (p-value): 0.357

Difference-in-Hansen test (p-value): 0.397

- Reduced models:

Table A17: Model 1a

| oop_che | Coef. | | St.Err. | t-value | | p-value | [95% Conf | | Interval] | | Sig |
| --- | --- | --- | --- | --- | --- | --- | --- | --- | --- | --- | --- |
| L. oop_che | .68 | | .06 | 10.98 | | 0 | .55 | | .8 | | *** |
| ln_odaplus_health_pub_gdp | -.03 | | .26 | -0.13 | | .89 | -.55 | | .48 | |  |
| ln_odaplus_health_civpriv_gdp | -.09 | | .24 | -0.39 | | .7 | -.57 | | .38 | |  |
| ln_debt_serv_gdp | .18 | | .57 | 0.31 | | .76 | -.96 | | 1.31 | |  |
| ln_ghes_gdp | -7.9 | | 1.49 | -5.31 | | 0 | -10.85 | | -4.95 | | *** |
| ln_gdp_cap_c | .29 | | 2.05 | 0.14 | | .89 | -3.79 | | 4.36 | |  |
| imr | -.13 | | .05 | -2.36 | | .02 | -.23 | | -.02 | | ** |
| gov_eff | .36 | | 1.2 | 0.30 | | .76 | -2.02 | | 2.75 | |  |
| corr_control | -.73 | | 2.05 | -0.35 | | .72 | -4.79 | | 3.34 | |  |
| battle_dum | .51 | | .81 | 0.63 | | .53 | -1.1 | | 2.12 | |  |
| col_indfrom_uk | -2.72 | | 1.91 | -1.42 | | .16 | -6.5 | | 1.07 | |  |
| col_indfrom_fra | -.36 | | 1.82 | -0.20 | | .84 | -3.97 | | 3.25 | |  |
| col_indfrom_spa | .59 | | 1.45 | 0.41 | | .68 | -2.29 | | 3.47 | |  |
| Mean dependent var | | 40.102 | | | SD dependent var | | | 19.365 | |  |  |
| Number of obs | | 1443 | | | F-test | | | 483.073 | |  |  |
| **** p<.01, ** p<.05, * p<.1* | | | | | | | | | | | |
|  | | | | | | | | | | | |

Countries: 105

Instrument count: 87

Lag limit (years): 6

AB test (AR2) (p-value): 0.926

Hansen test (p-value): 0.301

Difference-in-Hansen test (p-value): 0.228

Table A18: Model 2a

| ln_oop_gdp | Coef. | | St.Err. | t-value | | p-value | [95% Conf | | Interval] | | Sig |
| --- | --- | --- | --- | --- | --- | --- | --- | --- | --- | --- | --- |
| L. ln_oop_gdp | .92 | | .06 | 14.83 | | 0 | .8 | | 1.05 | | *** |
| ln_odaplus_health_pub_gdp | -.01 | | .01 | -1.22 | | .22 | -.03 | | .01 | |  |
| ln_odaplus_health_civpriv_gdp | -.01 | | .01 | -0.71 | | .48 | -.02 | | .01 | |  |
| ln_odaplus_nonhealth_civpriv_gdp | .02 | | .02 | 1.30 | | .2 | -.01 | | .06 | |  |
| ln_ghes_gdp | .11 | | .04 | 2.47 | | .02 | .02 | | .19 | | ** |
| ln_gdp_cap_c | -.14 | | .07 | -1.87 | | .06 | -.28 | | .01 | | * |
| imr | 0 | | 0 | -0.88 | | .38 | -.01 | | 0 | |  |
| gov_eff | .05 | | .06 | 0.97 | | .34 | -.06 | | .17 | |  |
| corr_control | -.03 | | .06 | -0.41 | | .68 | -.15 | | .1 | |  |
| battle_dum | .03 | | .02 | 1.42 | | .16 | -.01 | | .08 | |  |
| col_indfrom_uk | -.04 | | .06 | -0.74 | | .46 | -.15 | | .07 | |  |
| col_indfrom_fra | .03 | | .06 | 0.59 | | .56 | -.08 | | .15 | |  |
| col_indfrom_spa | .05 | | .08 | 0.70 | | .48 | -.1 | | .21 | |  |
| Mean dependent var | | 0.550 | | | SD dependent var | | | 0.733 | |  |  |
| Number of obs | | 1443 | | | F-test | | | 128.934 | |  |  |
| **** p<.01, ** p<.05, * p<.1* | | | | | | | | | | | |
|  | | | | | | | | | | | |

Countries: 105

Instrument count: 87

Lag limit (years): 6

AB test (AR2) (p-value): 0.518

Hansen test (p-value): 0.182

Difference-in-Hansen test (p-value): 0.375

Table A19: Model 3a

| ghes_che | Coef. | | St.Err. | t-value | | p-value | [95% Conf | | Interval] | | Sig |
| --- | --- | --- | --- | --- | --- | --- | --- | --- | --- | --- | --- |
| L. ghes_che | .71 | | .08 | 9.12 | | 0 | .56 | | .87 | | *** |
| L2. ghes_che | .22 | | .08 | 2.92 | | 0 | .07 | | .37 | | *** |
| ln_odaplus_nonhealth_civpriv_gdp | -.28 | | .52 | -0.53 | | .6 | -1.32 | | .76 | |  |
| ln_debt_serv_gdp | .05 | | .34 | 0.14 | | .89 | -.62 | | .72 | |  |
| ln_gdp_cap_c | -.18 | | 2.32 | -0.08 | | .94 | -4.78 | | 4.42 | |  |
| imr | -.03 | | .06 | -0.51 | | .61 | -.15 | | .09 | |  |
| gov_eff | -1.28 | | 1.31 | -0.98 | | .33 | -3.88 | | 1.31 | |  |
| corr_control | 2.82 | | 1.55 | 1.81 | | .07 | -.26 | | 5.89 | | * |
| battle_dum | -.21 | | .54 | -0.39 | | .7 | -1.28 | | .86 | |  |
| col_indfrom_uk | .22 | | .89 | 0.24 | | .81 | -1.55 | | 1.99 | |  |
| col_indfrom_fra | .35 | | .93 | 0.38 | | .71 | -1.5 | | 2.2 | |  |
| col_indfrom_spa | 1.42 | | 1.03 | 1.37 | | .17 | -.63 | | 3.46 | |  |
| Mean dependent var | | 40.566 | | | SD dependent var | | | 19.169 | |  |  |
| Number of obs | | 1356 | | | F-test | | | 2714.024 | |  |  |
| **** p<.01, ** p<.05, * p<.1* | | | | | | | | | | | |
|  | | | | | | | | | | | |

Countries: 105

Instrument count: 88

Lag limit (years): 8

AB test (AR2) (p-value): 0.647

Hansen test (p-value): 0.225

Difference-in-Hansen test (p-value): 0.721

Table A20: Model 4a

| ln_ghes_gdp | Coef. | | St.Err. | t-value | | p-value | [95% Conf | | Interval] | | Sig |
| --- | --- | --- | --- | --- | --- | --- | --- | --- | --- | --- | --- |
| L. ln_ghes_gdp | .74 | | .09 | 7.92 | | 0 | .55 | | .92 | | *** |
| ln_odaplus_health_pub_gdp | .01 | | .01 | 0.64 | | .52 | -.01 | | .02 | |  |
| ln_gdp_cap_c | .21 | | .1 | 1.99 | | .05 | 0 | | .41 | | ** |
| imr | 0 | | 0 | 0.42 | | .67 | 0 | | .01 | |  |
| gov_eff | -.04 | | .06 | -0.61 | | .54 | -.15 | | .08 | |  |
| corr_control | .03 | | .07 | 0.42 | | .68 | -.12 | | .18 | |  |
| battle_dum | .03 | | .03 | 1.03 | | .31 | -.03 | | .09 | |  |
| col_indfrom_uk | -.04 | | .06 | -0.73 | | .47 | -.15 | | .07 | |  |
| col_indfrom_fra | -.11 | | .07 | -1.49 | | .14 | -.26 | | .04 | |  |
| col_indfrom_spa | -.02 | | .07 | -0.35 | | .73 | -.16 | | .11 | |  |
| Mean dependent var | | 0.563 | | | SD dependent var | | | 0.721 | |  |  |
| Number of obs | | 1460 | | | F-test | | | 241.976 | |  |  |
| **** p<.01, ** p<.05, * p<.1* | | | | | | | | | | | |
|  | | | | | | | | | | | |

Countries: 105

Instrument count: 73

Lag limit (years): 7

AB test (AR2) (p-value): 0.200

Hansen test (p-value): 0.230

Difference-in-Hansen test (p-value): 0.317

**Two lags on independent variables of interest:**

- Full models (only significant variations shown):

Table A21: Model 2

| ln_oop_gdp | Coef. | | St.Err. | t-value | | p-value | [95% Conf | | Interval] | | Sig |
| --- | --- | --- | --- | --- | --- | --- | --- | --- | --- | --- | --- |
| L. ln_oop_gdp | .93 | | .07 | 12.62 | | 0 | .78 | | 1.08 | | *** |
| L2. ln_odaplus_health_pub_gdp | -.02 | | .01 | -1.95 | | .05 | -.04 | | 0 | | * |
| L2. ln_odaplus_health_civpriv_gdp | -.01 | | .01 | -1.56 | | .12 | -.03 | | 0 | |  |
| L2. ln_odaplus_nonhealth_pub_gdp | .01 | | .01 | 0.91 | | .37 | -.02 | | .04 | |  |
| L2. ln_odaplus_nonhealth_civpriv_gdp | -.03 | | .01 | -2.52 | | .01 | -.06 | | -.01 | | ** |
| ln_debt_serv_gdp | 0 | | .02 | -0.03 | | .98 | -.03 | | .03 | |  |
| ln_debt_stock_gdp | .02 | | .04 | 0.52 | | .6 | -.06 | | .1 | |  |
| imf_cond_ba1tot | 0 | | 0 | -0.68 | | .5 | 0 | | 0 | |  |
| imf_part | .02 | | .03 | 0.96 | | .34 | -.03 | | .07 | |  |
| ln_ghes_gdp | .07 | | .06 | 1.16 | | .25 | -.05 | | .18 | |  |
| ln_gdp_cap_c | -.22 | | .11 | -1.97 | | .05 | -.45 | | 0 | | * |
| imr | 0 | | 0 | -1.31 | | .19 | -.01 | | 0 | |  |
| gov_eff | .03 | | .06 | 0.50 | | .62 | -.09 | | .14 | |  |
| corr_control | -.01 | | .08 | -0.16 | | .87 | -.16 | | .14 | |  |
| battle_dum | .02 | | .02 | 0.85 | | .4 | -.03 | | .07 | |  |
| col_indfrom_uk | .03 | | .06 | 0.49 | | .63 | -.09 | | .15 | |  |
| col_indfrom_fra | .05 | | .06 | 0.79 | | .43 | -.07 | | .16 | |  |
| col_indfrom_spa | .06 | | .06 | 0.93 | | .36 | -.06 | | .18 | |  |
| Mean dependent var | | 0.547 | | | SD dependent var | | | 0.733 | |  |  |
| Number of obs | | 1326 | | | F-test | | | 132.998 | |  |  |
| **** p<.01, ** p<.05, * p<.1* | | | | | | | | | | | |
|  | | | | | | | | | | | |

Countries: 105

Instrument count: 76

Lag limit (years): 3

AB test (AR2) (p-value): 0.567

Hansen test (p-value): 0.151

Difference-in-Hansen test (p-value): 0.330

- Reduced models (only significant variations shown):

Table A22: Model 2a

| ln_oop_gdp | Coef. | | St.Err. | t-value | | p-value | [95% Conf | | Interval] | | Sig |
| --- | --- | --- | --- | --- | --- | --- | --- | --- | --- | --- | --- |
| L. ln_oop_gdp | .94 | | .05 | 17.51 | | 0 | .84 | | 1.05 | | *** |
| L2. ln_odaplus_health_pub_gdp | -.02 | | .01 | -2.29 | | .02 | -.04 | | 0 | | ** |
| L2. ln_odaplus_health_civpriv_gdp | -.02 | | .01 | -2.31 | | .02 | -.03 | | 0 | | ** |
| ln_odaplus_nonhealth_civpriv_gdp | .01 | | .02 | 0.54 | | .59 | -.02 | | .04 | |  |
| ln_ghes_gdp | .08 | | .04 | 2.08 | | .04 | 0 | | .16 | | ** |
| ln_gdp_cap_c | -.2 | | .07 | -2.97 | | 0 | -.33 | | -.07 | | *** |
| imr | 0 | | 0 | -1.39 | | .17 | -.01 | | 0 | |  |
| gov_eff | .06 | | .05 | 1.15 | | .25 | -.04 | | .16 | |  |
| corr_control | -.03 | | .05 | -0.64 | | .53 | -.12 | | .06 | |  |
| battle_dum | .04 | | .02 | 1.55 | | .12 | -.01 | | .08 | |  |
| col_indfrom_uk | 0 | | .05 | 0.07 | | .94 | -.1 | | .11 | |  |
| col_indfrom_fra | .02 | | .05 | 0.44 | | .66 | -.08 | | .12 | |  |
| col_indfrom_spa | .04 | | .05 | 0.86 | | .39 | -.05 | | .14 | |  |
| Mean dependent var | | 0.547 | | | SD dependent var | | | 0.733 | |  |  |
| Number of obs | | 1326 | | | F-test | | | 194.769 | |  |  |
| **** p<.01, ** p<.05, * p<.1* | | | | | | | | | | | |
|  | | | | | | | | | | | |

Countries: 105

Instrument count: 86

Lag limit (years): 6

AB test (AR2) (p-value): 0.595

Hansen test (p-value): 0.337

Difference-in-Hansen test (p-value): 0.687

Table A23: Model 4a

| ln_ghes_gdp | Coef. | | St.Err. | t-value | | p-value | [95% Conf | | Interval] | | Sig |
| --- | --- | --- | --- | --- | --- | --- | --- | --- | --- | --- | --- |
| L. ln_ghes_gdp | .7 | | .09 | 8.02 | | 0 | .53 | | .87 | | *** |
| L2. ln_odaplus_health_pub_gdp | -.02 | | .01 | -2.21 | | .03 | -.04 | | 0 | | ** |
| ln_gdp_cap_c | .14 | | .1 | 1.44 | | .15 | -.05 | | .34 | |  |
| imr | 0 | | 0 | 0.13 | | .9 | -.01 | | .01 | |  |
| gov_eff | 0 | | .06 | -0.06 | | .95 | -.12 | | .11 | |  |
| corr_control | .03 | | .09 | 0.29 | | .77 | -.16 | | .21 | |  |
| battle_dum | .06 | | .03 | 1.70 | | .09 | -.01 | | .13 | | * |
| col_indfrom_uk | -.01 | | .08 | -0.18 | | .86 | -.17 | | .14 | |  |
| col_indfrom_fra | -.13 | | .09 | -1.35 | | .18 | -.31 | | .06 | |  |
| col_indfrom_spa | -.02 | | .09 | -0.24 | | .81 | -.21 | | .16 | |  |
| Mean dependent var | | 0.571 | | | SD dependent var | | | 0.720 | |  |  |
| Number of obs | | 1356 | | | F-test | | | 111.931 | |  |  |
| **** p<.01, ** p<.05, * p<.1* | | | | | | | | | | | |
|  | | | | | | | | | | | |

Countries: 105

Instrument count: 72

Lag limit (years): 7

AB test (AR2) (p-value): 0.340

Hansen test (p-value): 0.470

Difference-in-Hansen test (p-value): 0.664

### Alternative lag limits

The below table shows how changing model lag limits affects the p-value of significant variables identified in our model specifications. P-values less than 0.05 are highlighted in **bold**. Insignificant variables are not shown. Dependent variable lags were significant in all models but not shown. The instrument matrix is collapsed for all specifications:

Table A24: Model 1

| oop_che | Lag limits | | | | | Direction of  association |
| --- | --- | --- | --- | --- | --- | --- |
| Independent variable | 2 | 3 | 4 | 5 | 6 |  |
| L. ln_odaplus_health_civpriv_gdp (p-value) | 0.078 | 0.085 | **0.021** | **0.012** | 0.076 | - |
| ln_ghes_gdp (p-value) | **0.000** | **0.000** | **0.000** | **0.000** | **0.000** | - |
| imr (p-value) | **0.024** | **0.010** | **0.038** | **0.006** | **0.011** | - |
| Hansen-test p-value | 0.206 | 0.506 | 0.262 | 0.558 | 0.856 |  |
| Instrument count | 62 | 77 | 92 | 107 | 122 |  |

Table A25: Model 2

| ln_oop_gdp | Lag limits | | | | | Direction of  association |
| --- | --- | --- | --- | --- | --- | --- |
| Independent variable | 2 | 3 | 4 | 5 | 6 |  |
| L. ln_odaplus_health_pub_gdp (p-value) | 0.227 | 0.082 | 0.086 | 0.099 | **0.027** | - |
| L. ln_odaplus_health_civpriv_gdp (p-value) | 0.107 | 0.078 | 0.050 | **0.005** | **0.028** | - |
| ln_ghes_gdp (p-value) | **0.018** | 0.063 | 0.092 | 0.105 | 0.067 | + |
| ln_gdp_cap_c (p-value) | 0.057 | **0.019** | **0.029** | **0.012** | **0.002** | - |
| Hansen-test p-value | 0.052 | 0.113 | 0.366 | 0.546 | 0.869 |  |
| Instrument count | 62 | 77 | 92 | 107 | 122 |  |

Model 3

None significant

Table A26: Model 4

| ln_ghes_gdp | Lag limits | | | | | Direction of  association |
| --- | --- | --- | --- | --- | --- | --- |
| Independent variable | 2 | 3 | 4 | 5 | 6 |  |
| ln_odaplus_health_pub_gdp (p-value) | 0.128 | 0.062 | 0.075 | **0.008** | **0.035** | - |
| Hansen-test p-value | 0.153 | 0.592 | 0.615 | 0.480 | 0.670 |  |
| Instrument count | 59 | 73 | 87 | 101 | 115 |  |

Table A27: Model 1a

| oop_che | Lag limits | | | | | Direction of  association |
| --- | --- | --- | --- | --- | --- | --- |
| Independent variable | 3 | 4 | 5 | 6 | 7 |  |
| L. ln_debt_serv_gdp (p-value) | 0.066 | 0.060 | **0.041** | 0.065 | 0.060 | + |
| ln_ghes_gdp (p-value) | **0.000** | **0.000** | **0.000** | **0.000** | **0.000** | - |
| imr (p-value) | **0.011** | **0.013** | **0.008** | **0.005** | **0.006** | - |
| Hansen-test p-value | 0.224 | 0.194 | 0.402 | 0.624 | 0.493 |  |
| Instrument count | 57 | 67 | 77 | 78 | 97 |  |

Table A28: Model 2a

| ln_oop_gdp | Lag limits | | | | | Direction of  association |
| --- | --- | --- | --- | --- | --- | --- |
| Independent variable | 5 | 6 | 7 | 8 | 9 |  |
| L. ln_odaplus_health_pub_gdp (p-value) | 0.107 | 0.061 | **0.017** | **0.007** | **0.002** | - |
| L. ln_odaplus_health_civpriv_gdp (p-value) | **0.048** | 0.092 | **0.047** | **0.036** | 0.067 | - |
| L. ln_odaplus_nonhealth_civpriv_gdp (p-value) | 0.121 | 0.050 | **0.037** | 0.061 | 0.097 | - |
| ln_ghes_gdp (p-value) | 0.085 | **0.030** | **0.048** | **0.039** | **0.016** | + |
| ln_gdp_cap_c (p-value) | **0.030** | **0.004** | **0.001** | **0.000** | **0.001** | - |
| Hansen-test p-value | 0.263 | 0.330 | 0.469 | 0.403 | 0.803 |  |
| Instrument count | 77 | 87 | 97 | 107 | 117 |  |

Table A29: Model 3a

| ghes_che | Lag limits | | | | | Direction of  association |
| --- | --- | --- | --- | --- | --- | --- |
| Independent variable | 6 | 7 | 8 | 9 | 10 |  |
| L. ln_debt_serv_gdp (p-value) | 0.061 | 0.101 | **0.023** | **0.038** | **0.042** | - |
| corr_control (p-value) | 0.075 | 0.111 | **0.017** | **0.010** | **0.027** | + |
| Hansen-test p-value | 0.224 | 0.296 | 0.242 | 0.223 | 0.211 |  |
| Instrument count | 72 | 80 | 88 | 96 | 109 |  |

Table A30: Model 4a

| ln_ghes_gdp | Lag limits | | | | | Direction of  association |
| --- | --- | --- | --- | --- | --- | --- |
| Independent variable | 5 | 6 | 7 | 8 | 9 |  |
| ln_odaplus_health_pub_gdp (p-value) | 0.082 | 0.065 | **0.045** | **0.037** | 0.143 | - |
| col_indfrom_fra (p-value) | 0.370 | 0.247 | 0.191 | 0.107 | **0.049** | - |
| Hansen-test p-value | 0.334 | 0.283 | 0.346 | 0.302 | 0.309 |  |
| Instrument count | 59 | 66 | 77 | 80 | 87 |  |

We also examined the sensitivity of our findings for IMF participation in a contemporaneous version of model 3:

Table A31: Model 3, contemporaneous specification

| ln_ghes_che | Lag limits | | | | | Direction of  association |
| --- | --- | --- | --- | --- | --- | --- |
| Independent variable | 2 | 3 | 4 | 5 | 6 |  |
| imf_part (p-value) | 0.389 | **0.029** | **0.036** | **0.042** | 0.085 | - |
| Hansen-test p-value | 0.359 | 0.430 | 0.302 | 0.450 | 0.829 |  |
| Instrument count | 59 | 73 | 87 | 101 | 115 |  |

### Alternative variables

The below table displays the sensitivity of our findings to swapping variables with alternative variables. Only significant variables shown. Lagged dependent variables significant in all models but not shown for brevity. Year dummies included in all models but not shown for brevity. Per GDP to per cap: All financial variables measured per GDP swapped to per capita, including dependent variables. IMR: Infant Mortality Rate. U5MR: Under-5-Mortality Rate. Cond: Conditionality variable (imf_cond_ba1tot swapped for imf_cond_ba2tot and imf_cond_ba3tot). Colony: Country colonial independence was obtained from. SSA: Sub-Saharan Africa. WB Inc: World Bank Income Group.

Italic used to highlight problematically high instrument counts necessary to maintain acceptable test statistics in alternative model, or problematically high Hansen-test p-values.

Table A32: Model 1

| oop_che |  |  |  |  |  |  |  |  |  | Direction of  association |
| --- | --- | --- | --- | --- | --- | --- | --- | --- | --- | --- |
| Original variable: | IMR | IMR | Cond. 1 | Cond. 1 | Colony | Colony | Colony | Per GDP |  |  |
| New variable: | U5MR | MMR | Cond. 2 | Cond. 3 | - | WB Inc. | SSA | Per cap.* |  |  |
| L. ln_odaplus_health_civpriv_gdp (p-value) | **0.009** | **0.013** | **0.039** | **0.028** | **0.027** | 0.069 | **0.034** | **0.023** |  | - |
| ln_ghes_gdp (p-value) | **0.000** | **0.000** | **0.000** | **0.000** | **0.000** | **0.000** | **0.000** | **0.000** |  | - |
| ln_gdp_cap_c (p-value) | 0.611 | 0.281 | 0.943 | 0.957 | 0.762 | 0.269 | 0.849 | **0.005** |  | + |
| imr (p-value) | 0.219 | 0.713 | **0.041** | **0.038** | **0.029** | **0.033** | 0.076 | 0.088 |  | - |
| Hansen-test p-value | 0.172 | 0.124 | 0.205 | 0.242 | 0.158 | 0.154 | 0.163 | 0.294 |  |  |
| Instrument count | 92 | 92 | 92 | 92 | 89 | 91 | 90 | 92 |  |  |

*Financial independent variables per capita.

Table A33: Model 2

| ln_oop_gdp |  |  |  |  |  |  |  |  |  | Direction of  association |
| --- | --- | --- | --- | --- | --- | --- | --- | --- | --- | --- |
| Original variable: | IMR | IMR | Cond. 1 | Cond. 1 | Colony | Colony | Colony | Per GDP |  |  |
| New variable: | U5MR | MMR | Cond. 2 | Cond. 3 | - | WB Inc. | SSA | Per cap.* |  |  |
| L. ln_odaplus_health_civpriv_gdp | 0.080 | 0.081 | **0.043** | **0.046** | **0.028** | 0.068 | **0.032** | 0.210 |  | - |
| ln_gdp_cap_c (p-value) | **0.045** | **0.049** | **0.031** | **0.029** | **0.014** | 0.102 | **0.019** | **0.037** |  | + |
| imr (p-value) | 0.639 | 0.862 | 0.335 | 0.344 | 0.198 | 0.389 | 0.076 | **0.027** |  | - |
| corr_control | 0.746 | 0.523 | 0.772 | 0.789 | 0.814 | 0.807 | 0.673 | **0.036** |  | + |
| Hansen-test p-value | 0.342 | 0.124 | 0.368 | 0.371 | 0.358 | 0.289 | 0.371 | 0.180 |  |  |
| Instrument count | 92 | 92 | 92 | 92 | 89 | 91 | 90 | *107* |  |  |

*Both dependent variable and financial independent variables per capita.

Model 3

None significant.

Table A34: Model 4

| ln_ghes_gdp |  |  |  |  |  |  |  |  |  | Direction of  association |
| --- | --- | --- | --- | --- | --- | --- | --- | --- | --- | --- |
| Original variable: | IMR | IMR | Cond. 1 | Cond. 1 | Colony | Colony | Colony | Per GDP |  |  |
| New variable: | U5MR | MMR | Cond. 2 | Cond. 3 | - | WB Inc. | SSA | Per cap.* |  |  |
| L. ln_odaplus_health_pub_gdp | **0.040** | **0.049** | **0.041** | **0.042** | 0.053 | 0.072 | **0.018** | 0.071 |  | - |
| L. ln_odaplus_nonhealth_pub_gdp | 0.756 | 0.567 | 0.870 | 0.805 | 0.786 | 0.778 | 0.762 | **0.019** |  | + |
| ln_gdp_cap_c (p-value) | 0.299 | 0.104 | 0.341 | 0.393 | 0.238 | 0.366 | 0.333 | **0.000** |  | + |
| Hansen-test p-value | *0.600* | 0.575 | 0.565 | 0.553 | *0.631* | *0.619* | *0.651* | 0.166 |  |  |
| Instrument count | 73 | 73 | 73 | 73 | 70 | 72 | 71 | 73 |  |  |

*Both dependent variable and financial independent variables per capita.

Table A35: Model 1a

| oop_che |  |  |  |  |  |  |  | Direction of  association |
| --- | --- | --- | --- | --- | --- | --- | --- | --- |
| Original variable: | IMR | IMR | Colony | Colony | Colony | Per GDP |  |  |
| New variable: | U5MR | MMR | - | WB Inc. | SSA | Per cap.* |  |  |
| L. ln_debt_serv_gdp (p-value) | 0.062 | 0.076 | **0.036** | **0.017** | **0.037** | 0.151 |  | + |
| ln_ghes_gdp (p-value) | **0.000** | **0.000** | **0.000** | **0.000** | **0.000** | **0.000** |  | - |
| ln_gdp_cap_c (p-value) | 0.893 | 0.874 | 0.528 | 0.604 | 0.485 | **0.018** |  | + |
| imr (p-value) | 0.138 | 0.638 | **0.005** | **0.013** | **0.015** | **0.021** |  | - |
| Hansen-test p-value | 0.367 | 0.238 | 0.400 | 0.310 | 0.404 | 0.396 |  |  |
| Instrument count | 97 | 97 | 97 | 74 | 75 | 77 |  |  |

*Financial independent variables per capita.

Table A36: Model 2a

| ln_oop_gdp |  |  |  |  |  |  |  | Direction of  association |
| --- | --- | --- | --- | --- | --- | --- | --- | --- |
| Original variable: | IMR | IMR | Colony | Colony | Colony | Per GDP |  |  |
| New variable: | U5MR | MMR | - | WB Inc. | SSA | Per cap.* |  |  |
| L. ln_odaplus_health_pub_gdp | **0.022** | **0.031** | **0.020** | 0.062 | **0.023** | 0.759 |  | - |
| L. ln_odaplus_health_civpriv_gdp | **0.018** | **0.024** | **0.024** | **0.015** | **0.040** | 0.717 |  | - |
| L. ln_odaplus_nonhealth_civpriv_gdp | **0.040** | **0.031** | **0.033** | **0.011** | **0.040** | 0.334 |  | - |
| ln_ghes_gdp | **0.044** | 0.071 | **0.082** | 0.386 | 0.067 | 0.440 |  | + |
| ln_gdp_cap_c | **0.006** | **0.008** | **0.001** | 0.094 | **0.001** | 0.139 |  | - |
| imr (p-value) | 0.138 | 0.638 | 0.072 | **0.013** | 0.179 | **0.002** |  | - |
| Hansen-test p-value | 0.473 | 0.467 | 0.438 | 0.194 | 0.465 | 0.195 |  |  |
| Instrument count | 97 | 97 | 94 | 96 | 95 | *107* |  |  |

*Both dependent variable and financial independent variables per capita.

Table A37: Model 3a

| ghes_che |  |  |  |  |  |  |  | Direction of  association |
| --- | --- | --- | --- | --- | --- | --- | --- | --- |
| Original variable: | IMR | IMR | Colony | Colony | Colony | Per GDP |  |  |
| New variable: | U5MR | MMR | - | WB Inc. | SSA | Per cap.* |  |  |
| L. ln_debt_serv_gdp | **0.022** | **0.014** | **0.019** | **0.012** | **0.028** | **0.020** |  | - |
| corr_control | **0.015** | **0.009** | **0.037** | 0.269 | 0.077 | **0.017** |  | + |
| Hansen-test p-value | 0.268 | 0.242 | 0.219 | 0.202 | 0.219 | 0.208 |  |  |
| Instrument count | 88 | 88 | 85 | 94 | 86 | 88 |  |  |

*Financial independent variables per capita.

Table A38: Model 4a

| ln_ghes_gdp |  |  |  |  |  |  |  | Direction of  association |
| --- | --- | --- | --- | --- | --- | --- | --- | --- |
| Original variable: | IMR | IMR | Colony | Colony | Colony | Per GDP |  |  |
| New variable: | U5MR | MMR | - | WB Inc. | SSA | Per cap.* |  |  |
| L. ln_odaplus_health_pub_gdp | **0.034** | **0.025** | **0.037** | **0.035** | **0.017** | 0.229 |  | - |
| ln_gdp_cap_c | 0.157 | **0.113** | 0.158 | 0.182 | 0.220 | **0.000** |  | + |
| Hansen-test p-value | 0.268 | 0.413 | 0.290 | 0.265 | 0.323 | 0.341 |  |  |
| Instrument count | 73 | 73 | 70 | 72 | 71 | *108* |  |  |

*Both dependent variable and financial independent variables per capita.

We also examined the sensitivity of our findings for IMF participation in a contemporaneous version of model 3:

Table A39: Model 3, contemporaneous specification

| ln_ghes_che |  |  |  |  |  |  |  | Direction of  association |
| --- | --- | --- | --- | --- | --- | --- | --- | --- |
| Original variable: | IMR | IMR | Colony | Colony | Colony | Per GDP |  |  |
| New variable: | U5MR | MMR | - | WB Inc. | SSA | Per cap.* |  |  |
| imf_part | **0.025** | 0.080 | **0.024** | **0.030** | **0.025** | **0.033** |  | - |
| Hansen-test p-value | 0.474 | *0.619* | 0.437 | 0.441 | 0.397 | 0.480 |  |  |
| Instrument count | 73 | 73 | 70 | 72 | 71 | 73 |  |  |

*Financial independent variables per capita.

### Interaction terms

Only models with significant interaction terms displayed. Year dummies included in all models but not shown for brevity. Problematic diagnostic test statistics highlighted in italic.

- Region being Sub-Saharan Africa:

Table A40: Model 3a

| ghes_che | Coef. | | St.Err. | t-value | | p-value | [95% Conf | | Interval] | | Sig |
| --- | --- | --- | --- | --- | --- | --- | --- | --- | --- | --- | --- |
| L. ghes_che | .72 | | .07 | 10.13 | | 0 | .58 | | .86 | | *** |
| L2. ghes_che | .24 | | .07 | 3.35 | | 0 | .1 | | .38 | | *** |
| L. ln_odaplus_nonhealth_civpriv_gdp | -.06 | | .43 | -0.14 | | .89 | -.91 | | .79 | |  |
| reg_ssf*L.ln_odaplus_nonhealth_civpriv_gdp | 2.37 | | .75 | 3.15 | | 0 | .88 | | 3.86 | | *** |
| L. ln_debt_serv_gdp | -.56 | | .48 | -1.17 | | .24 | -1.5 | | .38 | |  |
| reg_ssf*ln_debt_serv_gdp | .02 | | .56 | 0.04 | | .97 | -1.08 | | 1.12 | |  |
| ln_gdp_cap_c | .88 | | 2.6 | 0.34 | | .74 | -4.27 | | 6.03 | |  |
| imr | -.04 | | .06 | -0.57 | | .57 | -.16 | | .09 | |  |
| gov_eff | -1.02 | | 1.26 | -0.81 | | .42 | -3.53 | | 1.48 | |  |
| corr_control | 2.81 | | 1.64 | 1.72 | | .09 | -.43 | | 6.06 | | * |
| battle_dum | -.36 | | .67 | -0.54 | | .59 | -1.7 | | .97 | |  |
| reg_ssf | 3.26 | | 1.93 | 1.69 | | .09 | -.56 | | 7.08 | | * |
| Mean dependent var | | 40.566 | | | SD dependent var | | | 19.169 | |  |  |
| Number of obs | | 1356 | | | F-test | | | 2080.197 | |  |  |
| **** p<.01, ** p<.05, * p<.1* | | | | | | | | | | | |
|  | | | | | | | | | | | |

Countries: 105

Instrument count: 94

Lag limit (years): 7

AB test (AR2) (p-value): 0.693

Hansen test (p-value): 0.303

Difference-in-Hansen test (p-value): 0.804

- World Bank income group being low-income:

This model is possibly overspecified, and the two significant interaction terms become insignificant with any lag limit change.

Table A41: Model 4

| ln_ghes_gdp | Coef. | | St.Err. | t-value | | p-value | [95% Conf | | Interval] | | Sig |
| --- | --- | --- | --- | --- | --- | --- | --- | --- | --- | --- | --- |
| L. ln_ghes_gdp | .97 | | .05 | 18.03 | | 0 | .87 | | 1.08 | | *** |
| L. ln_odaplus_health_pub_gdp | -.01 | | .01 | -1.49 | | .14 | -.03 | | 0 | |  |
| inc_lic* L.ln_odaplus_health_pub_gdp | -.07 | | .03 | -2.13 | | .04 | -.13 | | 0 | | ** |
| L. ln_odaplus_health_civpriv_gdp | 0 | | .01 | -0.14 | | .89 | -.02 | | .01 | |  |
| inc_lic* L.ln_odaplus_health_civpriv_gdp | .06 | | .02 | 2.93 | | 0 | .02 | | .1 | | *** |
| L. ln_odaplus_nonhealth_pub_gdp | 0 | | .02 | 0.04 | | .97 | -.03 | | .03 | |  |
| L. ln_odaplus_nonhealth_civpriv_gdp | .02 | | .02 | 1.11 | | .27 | -.02 | | .06 | |  |
| L. ln_debt_serv_gdp | -.01 | | .01 | -0.49 | | .63 | -.03 | | .02 | |  |
| L. ln_debt_stock_gdp | -.02 | | .03 | -0.63 | | .53 | -.07 | | .04 | |  |
| imf_cond_ba1tot | 0 | | 0 | 0.08 | | .94 | 0 | | 0 | |  |
| imf_part | 0 | | .02 | -0.10 | | .92 | -.05 | | .04 | |  |
| ln_gdp_cap_c | -.05 | | .11 | -0.47 | | .64 | -.27 | | .16 | |  |
| imr | 0 | | 0 | 0.10 | | .92 | -.01 | | .01 | |  |
| gov_eff | .06 | | .05 | 1.19 | | .24 | -.04 | | .15 | |  |
| corr_control | .05 | | .05 | 1.02 | | .31 | -.05 | | .16 | |  |
| battle_dum | .03 | | .03 | 1.08 | | .28 | -.02 | | .08 | |  |
| inc_lic | -.05 | | .1 | -0.49 | | .62 | -.24 | | .15 | |  |
| Mean dependent var | | 0.557 | | | SD dependent var | | | 0.723 | |  |  |
| Number of obs | | 1429 | | | F-test | | | 571.200 | |  |  |
| **** p<.01, ** p<.05, * p<.1* | | | | | | | | | | | |
|  | | | | | | | | | | | |

Countries: 105

Instrument count: 77

Lag limit (years): 3

AB test (AR2) (p-value): 0.418

Hansen test (p-value): *0.679*

Difference-in-Hansen test (p-value): 0.306

### Aggregate ODA-variables

Year dummies included in all models but not shown for brevity.

Table A42: Model 1

| oop_che | Coef. | | St.Err. | t-value | | p-value | [95% Conf | | Interval] | | Sig |
| --- | --- | --- | --- | --- | --- | --- | --- | --- | --- | --- | --- |
| L. oop_che | .7 | | .05 | 14.15 | | 0 | .6 | | .8 | | *** |
| L. ln_odaplus_health_gdp | -.73 | | .39 | -1.89 | | .06 | -1.5 | | .04 | | * |
| L. ln_odaplus_nonhealth_gdp | .36 | | .57 | 0.63 | | .53 | -.77 | | 1.49 | |  |
| L. ln_debt_serv_gdp | .37 | | .27 | 1.34 | | .18 | -.18 | | .91 | |  |
| L. ln_debt_stock_gdp | .62 | | .77 | 0.80 | | .42 | -.91 | | 2.14 | |  |
| imf_cond_ba1tot | -.02 | | .01 | -1.33 | | .19 | -.04 | | .01 | |  |
| imf_part | .71 | | .52 | 1.37 | | .17 | -.32 | | 1.74 | |  |
| ln_ghes_gdp | -7.55 | | 1.39 | -5.43 | | 0 | -10.3 | | -4.79 | | *** |
| ln_gdp_cap_c | -1.15 | | 1.81 | -0.64 | | .52 | -4.74 | | 2.43 | |  |
| imr | -.12 | | .05 | -2.20 | | .03 | -.22 | | -.01 | | ** |
| gov_eff | 1.41 | | 1.5 | 0.94 | | .35 | -1.57 | | 4.39 | |  |
| corr_control | -.86 | | 1.56 | -0.55 | | .58 | -3.95 | | 2.23 | |  |
| battle_dum | .3 | | .75 | 0.40 | | .69 | -1.19 | | 1.79 | |  |
| col_indfrom_uk | -2.2 | | 1.51 | -1.46 | | .15 | -5.21 | | .8 | |  |
| col_indfrom_fra | -.73 | | 1.74 | -0.42 | | .68 | -4.19 | | 2.73 | |  |
| col_indfrom_spa | .36 | | 1.68 | 0.22 | | .83 | -2.97 | | 3.7 | |  |
| Mean dependent var | | 39.952 | | | SD dependent var | | | 19.411 | |  |  |
| Number of obs | | 1461 | | | F-test | | | 468.132 | |  |  |
| **** p<.01, ** p<.05, * p<.1* | | | | | | | | | | | |

Countries: 105

Instrument count: 95

Lag limit (years): 5

AB test (AR2) (p-value): 0.998

Hansen test (p-value): 0.257

Difference-in-Hansen test (p-value): 0.168

Table A43: Model 2

| ln_oop_gdp | Coef. | | St.Err. | t-value | | p-value | [95% Conf | | Interval] | | Sig |
| --- | --- | --- | --- | --- | --- | --- | --- | --- | --- | --- | --- |
| L. ln_oop_gdp | .88 | | .05 | 16.69 | | 0 | .78 | | .98 | | *** |
| L. ln_odaplus_health_gdp | -.03 | | .01 | -2.61 | | .01 | -.06 | | -.01 | | ** |
| L. ln_odaplus_nonhealth_gdp | 0 | | .02 | -0.08 | | .94 | -.05 | | .04 | |  |
| L. ln_debt_serv_gdp | .02 | | .01 | 1.17 | | .25 | -.01 | | .05 | |  |
| L. ln_debt_stock_gdp | -.02 | | .02 | -0.69 | | .49 | -.06 | | .03 | |  |
| imf_cond_ba1tot | 0 | | 0 | -0.91 | | .36 | 0 | | 0 | |  |
| imf_part | .03 | | .02 | 1.52 | | .13 | -.01 | | .07 | |  |
| ln_ghes_gdp | .07 | | .04 | 1.74 | | .08 | -.01 | | .14 | | * |
| ln_gdp_cap_c | -.17 | | .07 | -2.45 | | .02 | -.32 | | -.03 | | ** |
| imr | 0 | | 0 | -0.78 | | .44 | 0 | | 0 | |  |
| gov_eff | .02 | | .06 | 0.43 | | .67 | -.09 | | .14 | |  |
| corr_control | .01 | | .05 | 0.19 | | .85 | -.1 | | .12 | |  |
| battle_dum | .04 | | .02 | 1.56 | | .12 | -.01 | | .08 | |  |
| col_indfrom_uk | -.04 | | .05 | -0.79 | | .43 | -.14 | | .06 | |  |
| col_indfrom_fra | 0 | | .06 | 0.06 | | .95 | -.11 | | .12 | |  |
| col_indfrom_spa | .01 | | .05 | 0.16 | | .88 | -.09 | | .1 | |  |
| Mean dependent var | | 0.543 | | | SD dependent var | | | 0.737 | |  |  |
| Number of obs | | 1461 | | | F-test | | | 235.018 | |  |  |
| **** p<.01, ** p<.05, * p<.1* | | | | | | | | | | | |
| Countries: 105  Instrument count: 95  Lag limit (years): 5  AB test (AR2) (p-value): 0.626  Hansen test (p-value): 0.266  Difference-in-Hansen test (p-value): 0.360 | | | | | | | | | | | |

Table A44: Model 3

| ghes_che | Coef. | | St.Err. | t-value | | p-value | [95% Conf | | Interval] | | Sig |
| --- | --- | --- | --- | --- | --- | --- | --- | --- | --- | --- | --- |
| L. ghes_che | .69 | | .08 | 8.26 | | 0 | .52 | | .85 | | *** |
| L2. ghes_che | .2 | | .07 | 2.93 | | 0 | .06 | | .33 | | *** |
| L. ln_odaplus_health_gdp | -.26 | | .33 | -0.78 | | .44 | -.92 | | .4 | |  |
| L. ln_odaplus_nonhealth_gdp | -.83 | | .51 | -1.65 | | .1 | -1.84 | | .17 | |  |
| L. ln_debt_serv_gdp | -.23 | | .27 | -0.82 | | .41 | -.77 | | .32 | |  |
| L. ln_debt_stock_gdp | .43 | | .73 | 0.59 | | .56 | -1.02 | | 1.88 | |  |
| imf_cond_ba1tot | 0 | | .01 | 0.03 | | .98 | -.02 | | .03 | |  |
| imf_part | -.75 | | .63 | -1.19 | | .24 | -2 | | .5 | |  |
| ln_gdp_cap_c | -.43 | | 2.43 | -0.18 | | .86 | -5.26 | | 4.39 | |  |
| imr | -.03 | | .06 | -0.52 | | .61 | -.15 | | .09 | |  |
| gov_eff | -.63 | | 1.36 | -0.47 | | .64 | -3.32 | | 2.06 | |  |
| corr_control | 2.67 | | 2.21 | 1.21 | | .23 | -1.72 | | 7.06 | |  |
| battle_dum | -.39 | | .68 | -0.57 | | .57 | -1.74 | | .96 | |  |
| col_indfrom_uk | .25 | | 1.05 | 0.24 | | .81 | -1.84 | | 2.34 | |  |
| col_indfrom_fra | .65 | | .95 | 0.68 | | .5 | -1.24 | | 2.54 | |  |
| col_indfrom_spa | .3 | | 1.7 | 0.18 | | .86 | -3.07 | | 3.67 | |  |
| Mean dependent var | | 40.566 | | | SD dependent var | | | 19.169 | |  |  |
| Number of obs | | 1356 | | | F-test | | | 1856.911 | |  |  |
| **** p<.01, ** p<.05, * p<.1* | | | | | | | | | | | |
|  | | | | | | | | | | | |
| Countries: 105  Instrument count: 100  Lag limit (years): 6  AB test (AR2) (p-value): 0.593  Hansen test (p-value): 0.301  Difference-in-Hansen test (p-value): 0.450 | | | | | | | | | | | |

Table A45: Model 4

| ln_ghes_gdp | Coef. | | St.Err. | t-value | | p-value | [95% Conf | | Interval] | | Sig |
| --- | --- | --- | --- | --- | --- | --- | --- | --- | --- | --- | --- |
| L. ln_ghes_gdp | .76 | | .08 | 9.10 | | 0 | .59 | | .92 | | *** |
| L. ln_odaplus_health_gdp | -.02 | | .02 | -0.94 | | .35 | -.06 | | .02 | |  |
| L. ln_odaplus_nonhealth_gdp | .01 | | .03 | 0.40 | | .69 | -.04 | | .06 | |  |
| L. ln_debt_serv_gdp | -.01 | | .01 | -0.48 | | .63 | -.04 | | .02 | |  |
| L. ln_debt_stock_gdp | 0 | | .03 | -0.14 | | .89 | -.06 | | .05 | |  |
| imf_cond_ba1tot | 0 | | 0 | 0.22 | | .83 | 0 | | 0 | |  |
| imf_part | -.01 | | .02 | -0.23 | | .82 | -.05 | | .04 | |  |
| ln_gdp_cap_c | .08 | | .09 | 0.95 | | .34 | -.09 | | .26 | |  |
| imr | 0 | | 0 | -0.96 | | .34 | -.01 | | 0 | |  |
| gov_eff | -.03 | | .07 | -0.42 | | .67 | -.16 | | .11 | |  |
| corr_control | .02 | | .06 | 0.26 | | .79 | -.1 | | .13 | |  |
| battle_dum | .03 | | .03 | 0.86 | | .39 | -.04 | | .09 | |  |
| col_indfrom_uk | .05 | | .05 | 0.93 | | .36 | -.06 | | .16 | |  |
| col_indfrom_fra | -.05 | | .05 | -0.99 | | .32 | -.14 | | .05 | |  |
| col_indfrom_spa | .04 | | .06 | 0.64 | | .53 | -.08 | | .15 | |  |
| Mean dependent var | | 0.563 | | | SD dependent var | | | 0.721 | |  |  |
| Number of obs | | 1461 | | | F-test | | | 222.786 | |  |  |
| **** p<.01, ** p<.05, * p<.1* | | | | | | | | | | | |
| Countries: 105  Instrument count: 89  Lag limit (years): 5  AB test (AR2) (p-value): 0.179  Hansen test (p-value): 0.246  Difference-in-Hansen test (p-value): 0.204 | | | | | | | | | | | |
| Table A46: Model 1a oop_che \| Coef. \| \| St.Err. \| t-value \| \| p-value \| [95% Conf \| \| Interval] \| \| Sig; L. oop_che \| .68 \| \| .06 \| 11.56 \| \| 0 \| .56 \| \| .8 \| \| ***; L. ln_odaplus_health_gdp \| -.83 \| \| .44 \| -1.87 \| \| .06 \| -1.71 \| \| .05 \| \| *; L. ln_odaplus_nonhealth_gdp \| -.21 \| \| .86 \| -0.25 \| \| .8 \| -1.92 \| \| 1.49 \| \|; L. ln_debt_serv_gdp \| .71 \| \| .34 \| 2.08 \| \| .04 \| .03 \| \| 1.39 \| \| **; ln_ghes_gdp \| -7.59 \| \| 1.43 \| -5.29 \| \| 0 \| -10.43 \| \| -4.74 \| \| ***; ln_gdp_cap_c \| -2.71 \| \| 1.94 \| -1.39 \| \| .17 \| -6.56 \| \| 1.14 \| \|; imr \| -.12 \| \| .06 \| -1.96 \| \| .05 \| -.24 \| \| 0 \| \| *; gov_eff \| 1.8 \| \| 1.37 \| 1.31 \| \| .19 \| -.92 \| \| 4.52 \| \|; corr_control \| -.81 \| \| 1.67 \| -0.48 \| \| .63 \| -4.12 \| \| 2.51 \| \|; battle_dum \| .01 \| \| .72 \| 0.02 \| \| .99 \| -1.42 \| \| 1.44 \| \|; col_indfrom_uk \| -2.16 \| \| 1.8 \| -1.20 \| \| .23 \| -5.72 \| \| 1.41 \| \|; col_indfrom_fra \| -.43 \| \| 1.85 \| -0.23 \| \| .82 \| -4.09 \| \| 3.24 \| \|; col_indfrom_spa \| .03 \| \| 1.66 \| 0.02 \| \| .98 \| -3.26 \| \| 3.33 \| \|; Mean dependent var \| \| 39.952 \| \| \| SD dependent var \| \| \| 19.411 \| \| \|; Number of obs \| \| 1461 \| \| \| F-test \| \| \| 354.401 \| \| \|; **** p<.01, ** p<.05, * p<.1* \| \| \| \| \| \| \| \| \| \| \| Countries: 105 Instrument count: 71 Lag limit (years): 5 AB test (AR2) (p-value): 0.965 Hansen test (p-value): 0.507 Difference-in-Hansen test (p-value): 0.313 Table A47: Model 2a ln_oop_gdp \| Coef. \| \| St.Err. \| t-value \| \| p-value \| [95% Conf \| \| Interval] \| \| Sig; L. ln_oop_gdp \| .94 \| \| .05 \| 17.38 \| \| 0 \| .83 \| \| 1.05 \| \| ***; L. ln_odaplus_health_gdp \| -.05 \| \| .02 \| -2.86 \| \| .01 \| -.08 \| \| -.01 \| \| ***; L. ln_odaplus_nonhealth_gdp \| -.03 \| \| .03 \| -1.20 \| \| .23 \| -.08 \| \| .02 \| \|; ln_ghes_gdp \| .08 \| \| .04 \| 1.92 \| \| .06 \| 0 \| \| .16 \| \| *; ln_gdp_cap_c \| -.27 \| \| .07 \| -3.93 \| \| 0 \| -.41 \| \| -.13 \| \| ***; imr \| 0 \| \| 0 \| -2.15 \| \| .03 \| -.01 \| \| 0 \| \| **; gov_eff \| .02 \| \| .05 \| 0.37 \| \| .71 \| -.08 \| \| .11 \| \|; corr_control \| .04 \| \| .06 \| 0.74 \| \| .46 \| -.07 \| \| .15 \| \|; battle_dum \| .05 \| \| .03 \| 2.02 \| \| .05 \| 0 \| \| .11 \| \| **; col_indfrom_uk \| .03 \| \| .06 \| 0.54 \| \| .59 \| -.08 \| \| .14 \| \|; col_indfrom_fra \| .06 \| \| .06 \| 1.06 \| \| .29 \| -.05 \| \| .17 \| \|; col_indfrom_spa \| -.01 \| \| .07 \| -0.15 \| \| .88 \| -.15 \| \| .13 \| \|; Mean dependent var \| \| 0.543 \| \| \| SD dependent var \| \| \| 0.737 \| \| \|; Number of obs \| \| 1461 \| \| \| F-test \| \| \| 179.523 \| \| \|; **** p<.01, ** p<.05, * p<.1* \| \| \| \| \| \| \| \| \| \| \| Countries: 105 Instrument count: 98 Lag limit (years): 8 AB test (AR2) (p-value): 0.695 Hansen test (p-value): 0.240 Difference-in-Hansen test (p-value): 0.485 | | | | | | | | | | | |

Table A48: Model 3a

*L. ln_odaplus_health_gdp added to explore fungibility hypothesis in this model.*

2^nd^-order lag on dependent variable necessary to convincingly pass AB test (AR2).

| ghes_che | Coef. | | St.Err. | t-value | | p-value | [95% Conf | | Interval] | | Sig |
| --- | --- | --- | --- | --- | --- | --- | --- | --- | --- | --- | --- |
| L. ghes_che | .66 | | .09 | 7.78 | | 0 | .49 | | .83 | | *** |
| L2. ghes_che | .2 | | .07 | 3.14 | | 0 | .08 | | .33 | | *** |
| L. ln_odaplus_health_gdp | -.33 | | .36 | -0.91 | | .36 | -1.06 | | .39 | |  |
| L. ln_odaplus_nonhealth_gdp | -.21 | | .58 | -0.36 | | .72 | -1.36 | | .94 | |  |
| L. ln_debt_serv_gdp | -.41 | | .32 | -1.30 | | .2 | -1.05 | | .22 | |  |
| ln_gdp_cap_c | 1.69 | | 2.33 | 0.73 | | .47 | -2.92 | | 6.3 | |  |
| imr | .01 | | .05 | 0.28 | | .78 | -.09 | | .12 | |  |
| gov_eff | -1.34 | | 1.28 | -1.04 | | .3 | -3.88 | | 1.21 | |  |
| corr_control | 2.75 | | 1.78 | 1.55 | | .13 | -.78 | | 6.28 | |  |
| battle_dum | -.37 | | .69 | -0.55 | | .59 | -1.74 | | .99 | |  |
| col_indfrom_uk | .14 | | .89 | 0.16 | | .88 | -1.62 | | 1.9 | |  |
| col_indfrom_fra | -.1 | | 1.02 | -0.10 | | .92 | -2.12 | | 1.92 | |  |
| col_indfrom_spa | .86 | | 1.69 | 0.51 | | .61 | -2.5 | | 4.21 | |  |
| Mean dependent var | | 40.566 | | | SD dependent var | | | 19.169 | |  |  |
| Number of obs | | 1356 | | | F-test | | | 1262.818 | |  |  |
| **** p<.01, ** p<.05, * p<.1* | | | | | | | | | | | |
|  | | | | | | | | | | | |

Countries: 105

Instrument count: 97

Lag limit (years): 8

AB test (AR2) (p-value): 0.625

Hansen test (p-value): 0.184

Difference-in-Hansen test (p-value): 0.713

Table A49: Model 4a

| ln_ghes_gdp | Coef. | | St.Err. | t-value | | p-value | [95% Conf | | Interval] | | Sig |
| --- | --- | --- | --- | --- | --- | --- | --- | --- | --- | --- | --- |
| L. ln_ghes_gdp | .7 | | .08 | 8.60 | | 0 | .54 | | .86 | | *** |
| L. ln_odaplus_health_gdp | -.05 | | .03 | -1.61 | | .11 | -.11 | | .01 | |  |
| ln_gdp_cap_c | .07 | | .09 | 0.79 | | .43 | -.11 | | .25 | |  |
| imr | 0 | | 0 | -0.43 | | .67 | -.01 | | 0 | |  |
| gov_eff | -.05 | | .07 | -0.72 | | .48 | -.18 | | .08 | |  |
| corr_control | .05 | | .08 | 0.68 | | .5 | -.11 | | .22 | |  |
| battle_dum | .04 | | .03 | 1.10 | | .27 | -.03 | | .1 | |  |
| col_indfrom_uk | .06 | | .09 | 0.65 | | .52 | -.11 | | .22 | |  |
| col_indfrom_fra | -.08 | | .06 | -1.18 | | .24 | -.2 | | .05 | |  |
| col_indfrom_spa | -.03 | | .08 | -0.37 | | .71 | -.2 | | .14 | |  |
| Mean dependent var | | 0.563 | | | SD dependent var | | | 0.721 | |  |  |
| Number of obs | | 1461 | | | F-test | | | 108.486 | |  |  |
| **** p<.01, ** p<.05, * p<.1* | | | | | | | | | | | |
|  | | | | | | | | | | | |

Countries: 105

Instrument count: 73

Lag limit (years): 7

AB test (AR2) (p-value): 0.181

Hansen test (p-value): 0.343

Difference-in-Hansen test (p-value): 0.650

### No log-transformation

Year dummies included in all models but not shown for brevity. Problematic diagnostic tests and instrument counts highlighted in italic. Shown models have “cherry-picked” lag-structures, looking for significance (confirmation bias).

Table A50: Model 1

*We were unable to produce a specification that convincingly passes all diagnostic tests and where instruments do not outnumber groups. Below is for “cherry-picked” variable lag-specification, looking for significance (confirmation bias). Model is overspecified and becomes invalid at lower instrument counts. Findings for debt variables disappear at different ODA+ lag-specifications.*

| oop_che | Coef. | | St.Err. | t-value | | p-value | [95% Conf | | Interval] | | Sig |
| --- | --- | --- | --- | --- | --- | --- | --- | --- | --- | --- | --- |
| L. oop_che | .72 | | .06 | 11.54 | | 0 | .59 | | .84 | | *** |
| L. odaplus_health_pub_gdp | -.51 | | .8 | -0.64 | | .53 | -2.1 | | 1.08 | |  |
| L. odaplus_health_civpriv_gdp | -2.38 | | 2.08 | -1.15 | | .25 | -6.5 | | 1.74 | |  |
| odaplus_nonhealth_pub_gdp | -.1 | | .14 | -0.71 | | .48 | -.37 | | .17 | |  |
| odaplus_nonhealth_civpriv_gdp | -.44 | | .81 | -0.55 | | .59 | -2.05 | | 1.16 | |  |
| debt_serv_gdp | -.19 | | .09 | -2.07 | | .04 | -.37 | | -.01 | | ** |
| debt_stock_gdp | .06 | | .03 | 2.32 | | .02 | .01 | | .12 | | ** |
| imf_cond_ba1tot | 0 | | .01 | -0.34 | | .74 | -.03 | | .02 | |  |
| imf_part | .83 | | .62 | 1.33 | | .19 | -.4 | | 2.05 | |  |
| ghes_gdp | -3.38 | | .83 | -4.07 | | 0 | -5.02 | | -1.73 | | *** |
| gdp_cap_c | 0 | | 0 | -0.53 | | .6 | 0 | | 0 | |  |
| imr | -.13 | | .05 | -2.49 | | .01 | -.23 | | -.03 | | ** |
| gov_eff | -1.25 | | 1.56 | -0.80 | | .42 | -4.33 | | 1.84 | |  |
| corr_control | -.99 | | 1.77 | -0.56 | | .58 | -4.51 | | 2.52 | |  |
| battle_dum | .67 | | .85 | 0.79 | | .43 | -1.01 | | 2.35 | |  |
| col_indfrom_uk | -1.97 | | 1.56 | -1.26 | | .21 | -5.06 | | 1.12 | |  |
| col_indfrom_fra | -1.24 | | 1.72 | -0.72 | | .47 | -4.65 | | 2.17 | |  |
| col_indfrom_spa | .66 | | 1.65 | 0.40 | | .69 | -2.61 | | 3.93 | |  |
| Mean dependent var | | 40.183 | | | SD dependent var | | | 19.385 | |  |  |
| Number of obs | | 1433 | | | F-test | | | 391.839 | |  |  |
| **** p<.01, ** p<.05, * p<.1* | | | | | | | | | | | |
|  | | | | | | | | | | | |

Countries: 105

Instrument count: *107*

Lag limit (years): 5

AB test (AR2) (p-value): 1.000

Hansen test (p-value): 0.186

Difference-in-Hansen test (p-value): 0.230

Table A51: Model 2

| oop_gdp | Coef. | | St.Err. | t-value | | p-value | [95% Conf | | Interval] | | Sig |
| --- | --- | --- | --- | --- | --- | --- | --- | --- | --- | --- | --- |
| L. oop_gdp | .79 | | .09 | 8.97 | | 0 | .62 | | .97 | | *** |
| L. odaplus_health_pub_gdp | -.09 | | .04 | -2.11 | | .04 | -.17 | | 0 | | ** |
| odaplus_health_civpriv_gdp | -.02 | | .17 | -0.12 | | .9 | -.36 | | .32 | |  |
| L. odaplus_nonhealth_pub_gdp | .01 | | .01 | 0.47 | | .64 | -.02 | | .03 | |  |
| odaplus_nonhealth_civpriv_gdp | .11 | | .06 | 1.90 | | .06 | 0 | | .23 | | * |
| L. debt_serv_gdp | -.01 | | .02 | -0.58 | | .56 | -.04 | | .02 | |  |
| L. debt_serv_gdp | 0 | | 0 | -0.70 | | .49 | 0 | | 0 | |  |
| L2. imf_cond_ba1tot | 0 | | 0 | -1.51 | | .13 | 0 | | 0 | |  |
| L2. imf_part | .17 | | .05 | 3.46 | | 0 | .07 | | .27 | | *** |
| ghes_gdp | .02 | | .05 | 0.41 | | .68 | -.07 | | .11 | |  |
| gdp_cap_c | 0 | | 0 | -1.14 | | .26 | 0 | | 0 | |  |
| imr | 0 | | 0 | -1.10 | | .27 | -.01 | | 0 | |  |
| gov_eff | .17 | | .14 | 1.26 | | .21 | -.1 | | .45 | |  |
| corr_control | -.26 | | .22 | -1.16 | | .25 | -.7 | | .18 | |  |
| battle_dum | .13 | | .06 | 2.15 | | .03 | .01 | | .24 | | ** |
| col_indfrom_uk | -.08 | | .1 | -0.79 | | .43 | -.28 | | .12 | |  |
| col_indfrom_fra | -.06 | | .1 | -0.59 | | .56 | -.26 | | .14 | |  |
| col_indfrom_spa | -.06 | | .17 | -0.35 | | .72 | -.4 | | .28 | |  |
| Mean dependent var | | 2.146 | | | SD dependent var | | | 1.342 | |  |  |
| Number of obs | | 1347 | | | F-test | | | 287.666 | |  |  |
| **** p<.01, ** p<.05, * p<.1* | | | | | | | | | | | |
|  | | | | | | | | | | | |

Countries: 105

Instrument count: 91

Lag limit (years): 4

AB test (AR2) (p-value): 0.541

Hansen test (p-value): 0.235

Difference-in-Hansen test (p-value): 0.714

Table A52: Model 3

| ghes_che | Coef. | | St.Err. | t-value | | p-value | [95% Conf | | Interval] | | Sig |
| --- | --- | --- | --- | --- | --- | --- | --- | --- | --- | --- | --- |
| L. ghes_che | .84 | | .08 | 11.00 | | 0 | .69 | | .99 | | *** |
| L. odaplus_health_pub_gdp | -.86 | | .66 | -1.30 | | .2 | -2.16 | | .45 | |  |
| odaplus_health_civ_priv_gdp | .75 | | 1.1 | 0.68 | | .5 | -1.44 | | 2.93 | |  |
| L. odaplus_nonhealth_pub_gdp | -.03 | | .08 | -0.39 | | .7 | -.2 | | .13 | |  |
| odaplus_nonhealth_civpriv_gdp | -.28 | | .47 | -0.59 | | .55 | -1.2 | | .65 | |  |
| L. debt_serv_gdp | -.04 | | .1 | -0.41 | | .68 | -.25 | | .16 | |  |
| L. debt_stock_gdp | -.06 | | .02 | -2.46 | | .02 | -.1 | | -.01 | | ** |
| imf_cond_ba1tot | .01 | | .01 | 1.33 | | .19 | -.01 | | .04 | |  |
| imf_part | -.99 | | .58 | -1.70 | | .09 | -2.14 | | .17 | | * |
| gdp_cap_c | 0 | | 0 | 1.02 | | .31 | 0 | | 0 | |  |
| imr | -.05 | | .05 | -0.84 | | .4 | -.15 | | .06 | |  |
| gov_eff | -1.21 | | 1.3 | -0.92 | | .36 | -3.79 | | 1.38 | |  |
| corr_control | 2.21 | | 2.03 | 1.09 | | .28 | -1.81 | | 6.24 | |  |
| battle_dum | .4 | | .6 | 0.67 | | .5 | -.78 | | 1.58 | |  |
| col_indfrom_uk | -.03 | | 1.38 | -0.02 | | .98 | -2.78 | | 2.71 | |  |
| col_indfrom_fra | -.3 | | 1.38 | -0.22 | | .83 | -3.04 | | 2.43 | |  |
| col_indfrom_spa | .56 | | 1.67 | 0.33 | | .74 | -2.76 | | 3.88 | |  |
| Mean dependent var | | 40.261 | | | SD dependent var | | | 19.024 | |  |  |
| Number of obs | | 1447 | | | F-test | | | 667.835 | |  |  |
| **** p<.01, ** p<.05, * p<.1* | | | | | | | | | | | |
|  | | | | | | | | | | | |

Countries: 105

Instrument count: 73

Lag limit (years): 3

AB test (AR2) (p-value): 0.190

Hansen test (p-value): 0.378

Difference-in-Hansen test (p-value): 0.680

Table A53: Model 4

| ghes_gdp | Coef. | | St.Err. | t-value | | p-value | [95% Conf | | Interval] | | Sig |
| --- | --- | --- | --- | --- | --- | --- | --- | --- | --- | --- | --- |
| L. ghes_gdp | .89 | | .04 | 21.29 | | 0 | .81 | | .98 | | *** |
| L. odaplus_health_pub_gdp | -.05 | | .03 | -1.44 | | .15 | -.11 | | .02 | |  |
| odaplus_health_civpriv_gdp | .17 | | .08 | 2.12 | | .04 | .01 | | .32 | | ** |
| L2. odaplus_nonhealth_pub_gdp | 0 | | .01 | -0.50 | | .62 | -.01 | | .01 | |  |
| odaplus_nonhealth_civpriv_gdp | .01 | | .03 | 0.47 | | .64 | -.05 | | .08 | |  |
| L. debt_serv_gdp | 0 | | .01 | 0.13 | | .89 | -.01 | | .02 | |  |
| L. debt_stock_gdp | 0 | | 0 | 0.23 | | .82 | 0 | | 0 | |  |
| L. imf_cond_ba1tot | 0 | | 0 | -0.90 | | .37 | 0 | | 0 | |  |
| L. imf_part | .05 | | .05 | 1.03 | | .31 | -.05 | | .15 | |  |
| gdp_cap_c | 0 | | 0 | -0.03 | | .98 | 0 | | 0 | |  |
| imr | 0 | | 0 | -0.91 | | .37 | -.01 | | 0 | |  |
| gov_eff | .07 | | .09 | 0.79 | | .43 | -.11 | | .26 | |  |
| corr_control | .13 | | .1 | 1.23 | | .22 | -.08 | | .33 | |  |
| battle_dum | .01 | | .04 | 0.38 | | .71 | -.06 | | .09 | |  |
| col_indfrom_uk | -.01 | | .06 | -0.18 | | .86 | -.14 | | .12 | |  |
| col_indfrom_fra | 0 | | .05 | 0.06 | | .95 | -.1 | | .11 | |  |
| col_indfrom_spa | .17 | | .1 | 1.61 | | .11 | -.04 | | .37 | |  |
| Mean dependent var | | 2.225 | | | SD dependent var | | | 1.429 | |  |  |
| Number of obs | | 1347 | | | F-test | | | 1114.232 | |  |  |
| **** p<.01, ** p<.05, * p<.1* | | | | | | | | | | | |
|  | | | | | | | | | | | |

Countries: 105

Instrument count: 86

Lag limit (years): 4

AB test (AR2) (p-value): 0.535

Hansen test (p-value): 0.204

Difference-in-Hansen test (p-value): 0.779

Table A54: Model 1a

| oop_che | Coef. | | St.Err. | t-value | | p-value | [95% Conf | | Interval] | | Sig |
| --- | --- | --- | --- | --- | --- | --- | --- | --- | --- | --- | --- |
| L. oop_che | .72 | | .05 | 13.28 | | 0 | .62 | | .83 | | *** |
| L2. odaplus_health_pub_gdp | .05 | | .7 | 0.08 | | .94 | -1.34 | | 1.45 | |  |
| L. odaplus_health_civpriv_gdp | -3.68 | | 2.76 | -1.33 | | .19 | -9.16 | | 1.79 | |  |
| L2. debt_serv_gdp | .14 | | .13 | 1.08 | | .28 | -.11 | | .39 | |  |
| ghes_gdp | -3.5 | | .83 | -4.19 | | 0 | -5.15 | | -1.84 | | *** |
| gdp_cap_c | 0 | | 0 | 0.06 | | .95 | 0 | | 0 | |  |
| imr | -.15 | | .05 | -2.97 | | 0 | -.25 | | -.05 | | *** |
| gov_eff | -.2 | | 1.5 | -0.13 | | .89 | -3.18 | | 2.78 | |  |
| corr_control | -3.95 | | 1.97 | -2.01 | | .05 | -7.85 | | -.06 | | ** |
| battle_dum | .29 | | .64 | 0.45 | | .65 | -.98 | | 1.55 | |  |
| col_indfrom_uk | -1.85 | | 1.36 | -1.36 | | .18 | -4.55 | | .86 | |  |
| col_indfrom_fra | -.81 | | 1.45 | -0.56 | | .58 | -3.69 | | 2.07 | |  |
| col_indfrom_spa | -.91 | | 1.77 | -0.51 | | .61 | -4.42 | | 2.6 | |  |
| Mean dependent var | | 39.858 | | | SD dependent var | | | 19.368 | |  |  |
| Number of obs | | 1343 | | | F-test | | | 553.256 | |  |  |
| **** p<.01, ** p<.05, * p<.1* | | | | | | | | | | | |
|  | | | | | | | | | | | |

Countries: 105

Instrument count: 96

Lag limit (years): 7

AB test (AR2) (p-value): 0.732

Hansen test (p-value): 0.332

Difference-in-Hansen test (p-value): 0.832

Table A55: Model 2a

| oop_gdp | Coef. | | St.Err. | t-value | | p-value | [95% Conf | | Interval] | | Sig |
| --- | --- | --- | --- | --- | --- | --- | --- | --- | --- | --- | --- |
| L. oop_gdp | .74 | | .11 | 6.43 | | 0 | .51 | | .96 | | *** |
| L2. odaplus_health_pub_gdp | -.12 | | .06 | -1.99 | | .05 | -.25 | | 0 | | ** |
| L2. odaplus_health_civpriv_gdp | -.02 | | .13 | -0.15 | | .88 | -.27 | | .23 | |  |
| odaplus_nonhealth_civpriv_gdp | .11 | | .05 | 2.12 | | .04 | .01 | | .21 | | ** |
| ghes_gdp | -.03 | | .05 | -0.60 | | .55 | -.12 | | .06 | |  |
| gdp_cap_c | 0 | | 0 | -1.96 | | .05 | 0 | | 0 | | * |
| imr | 0 | | 0 | -1.29 | | .2 | -.01 | | 0 | |  |
| gov_eff | .1 | | .1 | 1.00 | | .32 | -.1 | | .31 | |  |
| corr_control | -.12 | | .17 | -0.71 | | .48 | -.47 | | .22 | |  |
| battle_dum | .1 | | .07 | 1.53 | | .13 | -.03 | | .24 | |  |
| col_indfrom_uk | -.21 | | .14 | -1.48 | | .14 | -.48 | | .07 | |  |
| col_indfrom_fra | -.13 | | .14 | -0.94 | | .35 | -.4 | | .14 | |  |
| col_indfrom_spa | -.05 | | .21 | -0.25 | | .81 | -.47 | | .36 | |  |
| Mean dependent var | | 2.157 | | | SD dependent var | | | 1.347 | |  |  |
| Number of obs | | 1330 | | | F-test | | | 225.319 | |  |  |
| **** p<.01, ** p<.05, * p<.1* | | | | | | | | | | | |
|  | | | | | | | | | | | |

Countries: 105

Instrument count: 86

Lag limit (years): 6

AB test (AR2) (p-value): 0.590

Hansen test (p-value): 0.337

Difference-in-Hansen test (p-value): 0.384

Table A56: Model 3a

| ghes_che | Coef. | | St.Err. | t-value | | p-value | [95% Conf | | Interval] | | Sig |
| --- | --- | --- | --- | --- | --- | --- | --- | --- | --- | --- | --- |
| L. ghes_che | .74 | | .08 | 9.15 | | 0 | .58 | | .89 | | *** |
| L2. ghes_che | .2 | | .07 | 2.69 | | .01 | .05 | | .34 | | *** |
| odaplus_nonhealth_civpriv_gdp | -1.13 | | .51 | -2.20 | | .03 | -2.14 | | -.11 | | ** |
| debt_serv_gdp | -.02 | | .09 | -0.17 | | .86 | -.2 | | .17 | |  |
| gdp_cap_c | 0 | | 0 | 1.35 | | .18 | 0 | | 0 | |  |
| imr | .01 | | .06 | 0.08 | | .93 | -.11 | | .12 | |  |
| gov_eff | -1.4 | | 1.12 | -1.25 | | .21 | -3.61 | | .82 | |  |
| corr_control | 1.5 | | 1.84 | 0.82 | | .42 | -2.14 | | 5.14 | |  |
| battle_dum | -.19 | | .54 | -0.35 | | .73 | -1.25 | | .88 | |  |
| col_indfrom_uk | .02 | | .87 | 0.02 | | .98 | -1.71 | | 1.75 | |  |
| col_indfrom_fra | -.15 | | .69 | -0.22 | | .83 | -1.51 | | 1.21 | |  |
| col_indfrom_spa | .06 | | 1.24 | 0.05 | | .96 | -2.41 | | 2.53 | |  |
| Mean dependent var | | 40.566 | | | SD dependent var | | | 19.169 | |  |  |
| Number of obs | | 1356 | | | F-test | | | 2750.879 | |  |  |
| **** p<.01, ** p<.05, * p<.1* | | | | | | | | | | | |
|  | | | | | | | | | | | |

Countries: 105

Instrument count: 64

Lag limit (years): 5

AB test (AR2) (p-value): 0.558

Hansen test (p-value): 0.191

Difference-in-Hansen test (p-value): 0.787

Table A57: Model 4a

| ghes_gdp | Coef. | | St.Err. | t-value | | p-value | [95% Conf | | Interval] | | Sig |
| --- | --- | --- | --- | --- | --- | --- | --- | --- | --- | --- | --- |
| L. ghes_gdp | .95 | | .05 | 20.53 | | 0 | .86 | | 1.04 | | *** |
| L. odaplus_health_pub_gdp | .07 | | .09 | 0.81 | | .42 | -.11 | | .25 | |  |
| gdp_cap_c | 0 | | 0 | -0.26 | | .79 | 0 | | 0 | |  |
| imr | 0 | | 0 | -1.16 | | .25 | -.01 | | 0 | |  |
| gov_eff | .05 | | .08 | 0.65 | | .51 | -.1 | | .2 | |  |
| corr_control | .06 | | .09 | 0.67 | | .51 | -.12 | | .25 | |  |
| battle_dum | .03 | | .04 | 0.83 | | .41 | -.05 | | .12 | |  |
| col_indfrom_uk | 0 | | .05 | -0.01 | | .99 | -.1 | | .1 | |  |
| col_indfrom_fra | .02 | | .05 | 0.44 | | .66 | -.08 | | .13 | |  |
| col_indfrom_spa | .09 | | .08 | 1.11 | | .27 | -.07 | | .25 | |  |
| Mean dependent var | | 2.210 | | | SD dependent var | | | 1.420 | |  |  |
| Number of obs | | 1460 | | | F-test | | | 1705.671 | |  |  |
| **** p<.01, ** p<.05, * p<.1* | | | | | | | | | | | |
|  | | | | | | | | | | | |

Countries: 105

Instrument count: 73

Lag limit (years): 7

AB test (AR2) (p-value): *0.085*

Hansen test (p-value): 0.173

Difference-in-Hansen test (p-value): 0.602

### With Inverse Mills Ratios

Below is shown the results of the used Probit regression of known factors predicting IMF programme participation following the available literature (16, 66-69). Alternative specifications were tested as well, however this model proved superior when weighing predictive capacity, parsimoniousness and data availability.

Table A58: Probit regression

| imf_part | Coef. | | St.Err. | t-value | | p-value | [95% Conf | | Interval] | | Sig |
| --- | --- | --- | --- | --- | --- | --- | --- | --- | --- | --- | --- |
| L. imf_part | 2.04 | | .1 | 20.59 | | 0 | 1.84 | | 2.23 | | *** |
| L. cur_acc_bal_gdp | -.02 | | .01 | -3.52 | | 0 | -.03 | | -.01 | | *** |
| L. res_imp | -.07 | | .02 | -3.25 | | 0 | -.12 | | -.03 | | *** |
| L. excrate | 0 | | 0 | -1.79 | | .07 | 0 | | 0 | | * |
| L. gdp_growth | -.02 | | .01 | -1.77 | | .08 | -.05 | | 0 | | * |
| L. ln_gdp_cap_c | -.18 | | .06 | -2.91 | | 0 | -.3 | | -.06 | | *** |
| L. voice_acc | .1 | | .09 | 1.21 | | .22 | -.06 | | .27 | |  |
| Constant | .47 | | .53 | 0.90 | | .37 | -.56 | | 1.51 | |  |
| Mean dependent var | | 0.311 | | | SD dependent var | | | 0.463 | |  |  |
| Pseudo r-squared | | 0.458 | | | Number of obs | | | 1245 | |  |  |
| Chi-square | | 707.303 | | | Prob > chi2 | | | 0.000 | |  |  |
| Akaike crit. (AIC) | | 851.934 | | | Bayesian crit. (BIC) | | | 892.949 | |  |  |
| **** p<.01, ** p<.05, * p<.1* | | | | | | | | | | | |
|  | | | | | | | | | | | |

*imf_part* is a binary variable capturing IMF programme participation, based on (15), *cur_acc_bal_gdp* is the current account balance in % of GDP (36, 70); *res_imp* is the total reserves in months of imports (36, 70); *excrate* is the official exchange rate in local currency units per US$ (36, 70); *gdp_growth* is GDP growth in % (36); *voice_acc* is Voice and Accountability from the Worldwide Governance Indicators (WGI) dataset (40), measured in standard deviations over a normal distribution. It captures perceptions of to what extent citizens are able to select their government, as well as freedoms of expression, association and media. Other authors (16, 66, 68) have used a Democracy estimate from the Polity5 dataset, however support for this data ended in 2020, and the dataset does not cover 2019 (71). For these reasons, we proxied democracy using the WGI variable, which showed the strongest correlation with IMF programme participation among the six main variables in the dataset.

Similar to Kentikelenis, Stubbs et al. (16, 66), below is shown a classification table illustrating the predictive capacity of the above Probit regression model.

|  | | True | | |
| --- | --- | --- | --- | --- |
|  |  | + | - | Total |
| Classified | + | 311 | 79 | 390 |
|  | - | 76 | 779 | 855 |
|  | Total | 387 | 858 | 1245 |

This model correctly classified 87.6% of country-year observations:

$\frac{true positives+true negatives}{total observations}*100\%=$ $\frac{311+779}{1245}*100\%=87.6\%$

Below is shown regression results from models 1-4 when including Inverse Mills Ratios. Year dummies included in all models but not shown for brevity. Please note: The inclusion of predictive variables for IMF participation lowered the number of countries with data from 105 to 92, and observations from 1429 to 1215. Instrument lag limits were adjusted accordingly to avoid overspecification. Potentially problematic diagnostic test statistics are highlighted in *italic*.

Adding Inverse Mills Ratios to our full models did not cause any substantive changes to our overall results or conclusions. Changes to coefficients and p-values seen were most likely due to the loss of degrees of freedom resulting from the inclusion of new explanatory variables with incomplete data for our Heckman selection model (214 observations and 13 units of observation lost), and the accompanying need to adjust some model lag limits to avoid overspecification. This resulted in the p-value for the relationship between ODA+ for health via civil/private sector per GDP and OOP/CHE being pushed from 0.02 to 0.12, and that for the relationship with OOP/GDP from 0.05 to 0.02. If the two explanatory variables with significant missing data were dropped (current account balance and total reserves), the relationship with OOP/CHE became significant again, with similar predictive capacity of the probit model. The p-value for the relationship between ODA+ for health channelled via the public sector per GDP and GHE-S/GDP went from 0.04 to 0.07, however it regained its significance at 1 deeper lag limit on GMM-style instruments. The negative relationship between IMF participation and GHE-S/CHE in a contemporaneous model became stronger, from -1.13 percentage points to -1.57 percentage points, though still with an insignificant Inverse Mills Ratio. The finding remained sensitive to the lag structure of other variables.

Table A59: Model 1

| oop_che | Coef. | | St.Err. | t-value | | p-value | [95% Conf | | Interval] | | Sig |
| --- | --- | --- | --- | --- | --- | --- | --- | --- | --- | --- | --- |
| L. oop_che | .71 | | .05 | 15.53 | | 0 | .62 | | .8 | | *** |
| L. ln_odaplus_health_pub_gdp | -.4 | | .35 | -1.13 | | .26 | -1.09 | | .3 | |  |
| L. ln_odaplus_health_civpriv_gdp | -.3 | | .19 | -1.57 | | .12 | -.69 | | .08 | |  |
| L. ln_odaplus_nonhealth_pub_gdp | -.19 | | .38 | -0.49 | | .62 | -.94 | | .56 | |  |
| L. ln_odaplus_nonhealth_civpriv_gdp | -.33 | | .49 | -0.66 | | .51 | -1.31 | | .65 | |  |
| L. ln_debt_serv_gdp | .39 | | .31 | 1.23 | | .22 | -.24 | | 1.01 | |  |
| L. ln_debt_stock_gdp | .49 | | .75 | 0.65 | | .52 | -1.01 | | 1.99 | |  |
| imf_cond_ba1tot | .01 | | .01 | 0.38 | | .7 | -.02 | | .03 | |  |
| imf_part | .39 | | .55 | 0.70 | | .48 | -.71 | | 1.48 | |  |
| ln_ghes_gdp | -6.83 | | 1.71 | -3.99 | | 0 | -10.24 | | -3.43 | | *** |
| ln_gdp_cap_c | -.78 | | 1.55 | -0.51 | | .61 | -3.86 | | 2.29 | |  |
| imr | -.15 | | .06 | -2.44 | | .02 | -.27 | | -.03 | | ** |
| gov_eff | -.33 | | 1.47 | -0.22 | | .82 | -3.25 | | 2.59 | |  |
| corr_control | -3.2 | | 2.22 | -1.44 | | .15 | -7.62 | | 1.21 | |  |
| battle_dum | .47 | | .82 | 0.58 | | .57 | -1.15 | | 2.09 | |  |
| col_indfrom_uk | -1.65 | | 1.35 | -1.22 | | .22 | -4.32 | | 1.03 | |  |
| col_indfrom_fra | -2.08 | | 1.68 | -1.24 | | .22 | -5.41 | | 1.25 | |  |
| col_indfrom_spa | -2.07 | | 1.72 | -1.20 | | .23 | -5.49 | | 1.35 | |  |
| Inverse Mills Ratio | .06 | | .26 | 0.24 | | .81 | -.45 | | .57 | |  |
| Mean dependent var | | 38.611 | | | SD dependent var | | | 19.623 | |  |  |
| Number of obs | | 1215 | | | F-test | | | 497.347 | |  |  |
| **** p<.01, ** p<.05, * p<.1* | | | | | | | | | | | |
|  | | | | | | | | | | | |

Countries: 92

Instrument count: 81

Lag limit (years): 3

AB test (AR2) (p-value): .701

Hansen test (p-value): 0.418

Difference-in-Hansen test (p-value): *0.148*

Table A60: Model 2

| ln_oop_gdp | Coef. | | St.Err. | t-value | | p-value | [95% Conf | | Interval] | | Sig |
| --- | --- | --- | --- | --- | --- | --- | --- | --- | --- | --- | --- |
| L. ln_oop_gdp | .89 | | .06 | 13.97 | | 0 | .77 | | 1.02 | | *** |
| L. ln_odaplus_health_pub_gdp | -.01 | | .01 | -0.99 | | .32 | -.03 | | .01 | |  |
| L. ln_odaplus_health_civpriv_gdp | -.02 | | .01 | -2.32 | | .02 | -.04 | | 0 | | ** |
| L. ln_odaplus_nonhealth_pub_gdp | -.01 | | .02 | -0.47 | | .64 | -.04 | | .03 | |  |
| L. ln_odaplus_nonhealth_civpriv_gdp | -.03 | | .02 | -1.24 | | .22 | -.08 | | .02 | |  |
| L. ln_debt_serv_gdp | .03 | | .02 | 1.60 | | .11 | -.01 | | .08 | |  |
| L. ln_debt_stock_gdp | -.04 | | .03 | -1.45 | | .15 | -.1 | | .02 | |  |
| imf_cond_ba1tot | 0 | | 0 | -0.08 | | .94 | 0 | | 0 | |  |
| imf_part | 0 | | .02 | 0.11 | | .92 | -.05 | | .05 | |  |
| ln_ghes_gdp | .04 | | .05 | 0.75 | | .46 | -.06 | | .13 | |  |
| ln_gdp_cap_c | -.16 | | .06 | -2.40 | | .02 | -.28 | | -.03 | | ** |
| imr | 0 | | 0 | -0.53 | | .6 | 0 | | 0 | |  |
| gov_eff | -.03 | | .05 | -0.74 | | .46 | -.13 | | .06 | |  |
| corr_control | .03 | | .07 | 0.38 | | .71 | -.11 | | .16 | |  |
| battle_dum | .03 | | .03 | 0.93 | | .35 | -.03 | | .09 | |  |
| col_indfrom_uk | -.02 | | .04 | -0.65 | | .52 | -.1 | | .05 | |  |
| col_indfrom_fra | .02 | | .06 | 0.37 | | .72 | -.1 | | .14 | |  |
| col_indfrom_spa | -.02 | | .07 | -0.26 | | .8 | -.15 | | .12 | |  |
| Inverse Mills Ratio | 0 | | .01 | 0.03 | | .97 | -.02 | | .02 | |  |
| Mean dependent var | | 0.508 | | | SD dependent var | | | 0.755 | |  |  |
| Number of obs | | 1215 | | | F-test | | | 108.790 | |  |  |
| **** p<.01, ** p<.05, * p<.1* | | | | | | | | | | | |
|  | | | | | | | | | | | |

Countries: 92

Instrument count: 81

Lag limit (years): 3

AB test (AR2) (p-value): 0.853

Hansen test (p-value): 0.278

Difference-in-Hansen test (p-value): 0.865

Table A61: Model 3

| ghes_che | Coef. | | St.Err. | t-value | | p-value | [95% Conf | | Interval] | | Sig |
| --- | --- | --- | --- | --- | --- | --- | --- | --- | --- | --- | --- |
| L. ghes_che | .83 | | .09 | 9.38 | | 0 | .65 | | 1 | | *** |
| L. ln_odaplus_health_pub_gdp | -.45 | | .27 | -1.69 | | .1 | -.97 | | .08 | | * |
| L. ln_odaplus_health_civpriv_gdp | .06 | | .25 | 0.22 | | .82 | -.45 | | .56 | |  |
| L. ln_odaplus_nonhealth_pub_gdp | -.07 | | .37 | -0.20 | | .84 | -.81 | | .66 | |  |
| L. ln_odaplus_nonhealth_civpriv_gdp | -.15 | | .39 | -0.37 | | .71 | -.93 | | .63 | |  |
| L. ln_debt_serv_gdp | -.1 | | .42 | -0.23 | | .82 | -.94 | | .75 | |  |
| L. ln_debt_stock_gdp | -.98 | | .86 | -1.14 | | .26 | -2.68 | | .73 | |  |
| imf_cond_ba1tot | .02 | | .02 | 0.96 | | .34 | -.02 | | .05 | |  |
| imf_part | -1.42 | | .74 | -1.92 | | .06 | -2.89 | | .05 | | * |
| ln_gdp_cap_c | -.37 | | 1.87 | -0.20 | | .84 | -4.08 | | 3.35 | |  |
| imr | -.05 | | .06 | -0.84 | | .4 | -.17 | | .07 | |  |
| gov_eff | .6 | | 1.98 | 0.30 | | .76 | -3.33 | | 4.53 | |  |
| corr_control | 2.38 | | 2.49 | 0.96 | | .34 | -2.57 | | 7.33 | |  |
| battle_dum | .23 | | .78 | 0.30 | | .77 | -1.32 | | 1.79 | |  |
| col_indfrom_uk | .17 | | 1.56 | 0.11 | | .92 | -2.92 | | 3.26 | |  |
| col_indfrom_fra | .21 | | 1.05 | 0.20 | | .84 | -1.88 | | 2.31 | |  |
| col_indfrom_spa | .54 | | 1.66 | 0.33 | | .74 | -2.75 | | 3.84 | |  |
| Inverse Mills Ratio | -.05 | | .32 | -0.16 | | .87 | -.7 | | .59 | |  |
| Mean dependent var | | 42.422 | | | SD dependent var | | | 19.049 | |  |  |
| Number of obs | | 1215 | | | F-test | | | 840.373 | |  |  |
| **** p<.01, ** p<.05, * p<.1* | | | | | | | | | | | |
|  | | | | | | | | | | | |

Countries: 92

Instrument count: 77

Lag limit (years): 3

AB test (AR2) (p-value): 0.151

Hansen test (p-value): 0.278

Difference-in-Hansen test (p-value): 0.354

Table A62: Model 4

| ln_ghes_gdp | Coef. | | St.Err. | t-value | | p-value | [95% Conf | | Interval] | | Sig |
| --- | --- | --- | --- | --- | --- | --- | --- | --- | --- | --- | --- |
| L. ln_ghes_gdp | .83 | | .06 | 13.16 | | 0 | .7 | | .95 | | *** |
| L. ln_odaplus_health_pub_gdp | -.02 | | .01 | -1.82 | | .07 | -.05 | | 0 | | * |
| L. ln_odaplus_health_civpriv_gdp | .01 | | .01 | 0.66 | | .51 | -.01 | | .02 | |  |
| L. ln_odaplus_nonhealth_pub_gdp | .01 | | .02 | 0.48 | | .63 | -.03 | | .04 | |  |
| L. ln_odaplus_nonhealth_civpriv_gdp | -.01 | | .02 | -0.58 | | .56 | -.05 | | .03 | |  |
| L. ln_debt_serv_gdp | 0 | | .02 | 0.26 | | .8 | -.03 | | .04 | |  |
| L. ln_debt_stock_gdp | -.05 | | .03 | -1.54 | | .13 | -.12 | | .02 | |  |
| imf_cond_ba1tot | 0 | | 0 | 0.02 | | .99 | 0 | | 0 | |  |
| imf_part | -.01 | | .03 | -0.54 | | .59 | -.07 | | .04 | |  |
| ln_gdp_cap_c | -.06 | | .07 | -0.78 | | .44 | -.2 | | .09 | |  |
| imr | 0 | | 0 | -1.00 | | .32 | -.01 | | 0 | |  |
| gov_eff | .04 | | .07 | 0.56 | | .58 | -.1 | | .18 | |  |
| corr_control | .08 | | .06 | 1.34 | | .18 | -.04 | | .2 | |  |
| battle_dum | .04 | | .03 | 1.29 | | .2 | -.02 | | .11 | |  |
| col_indfrom_uk | -.01 | | .06 | -0.11 | | .91 | -.12 | | .11 | |  |
| col_indfrom_fra | -.02 | | .05 | -0.43 | | .67 | -.11 | | .07 | |  |
| col_indfrom_spa | .04 | | .05 | 0.79 | | .43 | -.05 | | .13 | |  |
| Inverse Mills Ratio | -.01 | | .01 | -0.68 | | .5 | -.03 | | .02 | |  |
| Mean dependent var | | 0.638 | | | SD dependent var | | | 0.721 | |  |  |
| Number of obs | | 1215 | | | F-test | | | 213.828 | |  |  |
| **** p<.01, ** p<.05, * p<.1* | | | | | | | | | | | |
|  | | | | | | | | | | | |

Countries: 92

Instrument count: 77

Lag limit (years): 3

AB test (AR2) (p-value): 0.271

Hansen test (p-value): 0.426

Difference-in-Hansen test (p-value): 0.312

### Additional study limitations

Public debt can sometimes be underreported (e.g. (Reuters, 2024, Miriri, 2024)), which means our estimated debt effects should then serve as a lower bound.

We have addressed and discussed the potential issue of selection bias into IMF programmes, and our choice of covariates should have partially addressed the issue of selection bias in terms of who obtains development assistance including loans. Residual selection bias will however necessarily remain. Measurement error is also an omnipresent and underrecognized issue in the general development economic and global health economic literature (Jerven, 2013). We have used standardised data from the best available data sources for our research questions, removed countries with significant missing data, performed a range of sensitivity tests, and used GMM that helps overcome this source of endogeneity (Biørn, 2012), however some degree of bias from measurement error will remain by default (Jerven, 2013). Also, what is counted as ODA constitutes a heated topic of debate, and measurement methods change over time (Ritchie, 2023). However, we used OECD data directly in order to be able to disaggregate on- and off-budget ODA.

We have explored whether non-linear relationships were present by examining relevant interaction terms, however further non-linear relationships are conceivable.

## Metadata for variables in dataset

Table A63: Variable metadata

| Variable name | Description | Unit | Type | Original variable name in database | WB opendata code | Original source | URL | Comment |
| --- | --- | --- | --- | --- | --- | --- | --- | --- |
| *General* |  |  |  |  |  |  |  |  |
| countryname | Country name; Recipient country of development assistance; debtor country. | - | Text | - | - | - | - |  |
| iso3c | 3-letter isocode of country | - | Text | Country Code | - | Azevedo, J.P. (2011) "wbopendata: Stata module to access World Bank databases," Statistical Software Components S457234, Boston College Department of Economics. http://ideas.repec.org/c/boc/bocode/s457234.html | <https://databank.worldbank.org/data/source/world-development-indicators/preview/on> | Obtained with "wbopendata" command in Stata |
| iso3n | 3-digit isocode of country | - | Numerical | - | - | Raciborski, R. (2008). "kountry: A Stata utility for merging cross-country data from multiple sources," The Stata Journal, 8(3), 390-400. | <https://databank.worldbank.org/data/source/world-development-indicators/preview/on> | Obtained with "kountry" command in STATA |
| wb_regioncode | 3-letter code of World Bank Region | - | Text | region | - | *Reference:* Azevedo, J.P. (2011) "wbopendata: Stata module to access World Bank databases," Statistical Software Components S457234, Boston College Department of Economics. http://ideas.repec.org/c/boc/bocode/s457234.html | <https://databank.worldbank.org/data/source/world-development-indicators/preview/on> | Obtained with "wbopendata" command in Stata |
| wb_region | World Bank region name | - | Text | regionname | - | *Reference:* Azevedo, J.P. (2011) "wbopendata: Stata module to access World Bank databases," Statistical Software Components S457234, Boston College Department of Economics. http://ideas.repec.org/c/boc/bocode/s457234.html | <https://databank.worldbank.org/data/source/world-development-indicators/preview/on> | Obtained with "wbopendata" command in Stata |
| un_reg_det | United Nations region name (detailed) | - | Text | - | - | Raciborski, R. (2008). "kountry: A Stata utility for merging cross-country data from multiple sources," The Stata Journal, 8(3), 390-400;  United Nations Statistics Division, Methodology, Standard country or area codes for statistical use (M49) | https://unstats.un.org/unsd/methodology/m49/ | Obtained with "kountry" command in STATA |
| incomelevel | World Bank income group (abbreviation) | - | Text | incomelevel | - | *Reference:* Azevedo, J.P. (2011) "wbopendata: Stata module to access World Bank databases," Statistical Software Components S457234, Boston College Department of Economics. http://ideas.repec.org/c/boc/bocode/s457234.html | <https://databank.worldbank.org/data/source/world-development-indicators/preview/on> | Obtained with "wbopendata" command in Stata |
| incomelevelname | World Bank income group (full name) | - | Text | incomelevelname | - | *Reference:* Azevedo, J.P. (2011) "wbopendata: Stata module to access World Bank databases," Statistical Software Components S457234, Boston College Department of Economics. http://ideas.repec.org/c/boc/bocode/s457234.html | <https://databank.worldbank.org/data/source/world-development-indicators/preview/on> | Obtained with "wbopendata" command in Stata |
| lendingtype | World Bank lending group code | - | Text | lendingtype | - | *Reference:* Azevedo, J.P. (2011) "wbopendata: Stata module to access World Bank databases," Statistical Software Components S457234, Boston College Department of Economics. http://ideas.repec.org/c/boc/bocode/s457234.html | <https://databank.worldbank.org/data/source/world-development-indicators/preview/on> | Obtained with "wbopendata" command in Stata |
| lendingtypename | World Bank lending group name | - | Text | lendingtypename | - | *Reference:* Azevedo, J.P. (2011) "wbopendata: Stata module to access World Bank databases," Statistical Software Components S457234, Boston College Department of Economics. http://ideas.repec.org/c/boc/bocode/s457234.html | <https://databank.worldbank.org/data/source/world-development-indicators/preview/on> | Obtained with "wbopendata" command in Stata |
| year | Calendar year of development assistance disbursed, debt repayment made, health expenditures made, etc. | Number | Integer | - | - | - | - |  |
| *Health financing* |  |  |  |  |  |  |  |  |
| ghes_che | "Domestic General Government Health Expenditure (GGHE-D) as % Current Health Expenditure (CHE)" | % | continuous | "Domestic General Government Health Expenditure (GGHE-D) as % Current Health Expenditure (CHE)" | SH.XPD.GHED.CH.ZS | WHO Global Health Expenditure Database | <http://apps.who.int/nha/database/Select/Indicators/en> |  |
| ghes_cap | "Domestic General Government Health Expenditure (GGHE-D) per Capita in US$" | current US$ | continuous | "Domestic General Government Health Expenditure (GGHE-D) per Capita in US$" | SH.XPD.GHED.PC.CD | WHO Global Health Expenditure Database | <http://apps.who.int/nha/database/Select/Indicators/en> |  |
| ghes_gdp | "Domestic General Government Health Expenditure (GGHE-D) as % Gross Domestic Product (GDP)" | % | continuous | "Domestic General Government Health Expenditure (GGHE-D) as % Gross Domestic Product (GDP)" | SH.XPD.CHEX.GD.ZS | WHO Global Health Expenditure Database | <http://apps.who.int/nha/database/Select/Indicators/en> |  |
| oop_che | "Out-of-pocket (OOPS) as % of Current Health Expenditure (CHE)" | % | continuous | "Out-of-pocket (OOPS) as % of Current Health Expenditure (CHE)" | SH.XPD.OOPC.CH.ZS | WHO Global Health Expenditure Database | <http://apps.who.int/nha/database/Select/Indicators/en> |  |
| oop_cap | "Out-of-Pocket Expenditure (OOPS) per Capita in US$" | current US$ | continuous | "Out-of-Pocket Expenditure (OOPS) per Capita in US$" | SH.XPD.OOPC.PC.CD | WHO Global Health Expenditure Database | <http://apps.who.int/nha/database/Select/Indicators/en> |  |
| *Development assistance* |  |  |  |  |  |  |  |  |
| odaplus_health | Official Development Assistance (ODA) and Bill and Melinda Gates Foundation (BMGF) funding for health | current US$ | continuous | - | - | Organisation for Economic Co-operation and Development (OECD) Creditor Reporting System (CRS) | https://stats.oecd.org/ | For sectoral categories 120: "Health, Total" and 130: "Population Policies/Programmes & Reproductive Health, Total" - negative debt relief, administrative expenses and other in-donor country expenses |
| odaplus_health_pub | Official Development Assistance (ODA) and Bill and Melinda Gates Foundation (BMGF) funding for health channeled via the public sector | current US$ | continuous | - | - | Organisation for Economic Co-operation and Development (OECD) Creditor Reporting System (CRS) | https://stats.oecd.org/ | For sectoral categories 120: "Health, Total" and 130: "Population Policies/Programmes & Reproductive Health, Total" - negative debt relief, administrative expenses and other in-donor country expenses. |
| odaplus_health_civpriv | Official Development Assistance (ODA) and Bill and Melinda Gates Foundation (BMGF) funding for health channeled via NGO's & civil socity, and private sector institutions | current US$ | continuous | - | - | Organisation for Economic Co-operation and Development (OECD) Creditor Reporting System (CRS) | https://stats.oecd.org/ | For sectoral categories 120: "Health, Total" and 130: "Population Policies/Programmes & Reproductive Health, Total" - negative debt relief, administrative expenses and other in-donor country expenses. |
| odaplus_nonhealth | Official Development Assistance (ODA) and Bill and Melinda Gates Foundation (BMGF) funding not for health | current US$ | continuous | - | - | Organisation for Economic Co-operation and Development (OECD) Creditor Reporting System (CRS) | https://stats.oecd.org/ | For all other categories - negative debt relief, administrative expenses and other in-donor country expenses |
| odaplus_nonhealth_pub | Official Development Assistance (ODA) and Bill and Melinda Gates Foundation (BMGF) funding not for health channelled via public sector | current US$ | continuous | - | - | Organisation for Economic Co-operation and Development (OECD) Creditor Reporting System (CRS) | https://stats.oecd.org/ | For all other categories - negative debt relief, administrative expenses and other in-donor country expenses |
| odaplus_nonhealth_civpriv | Official Development Assistance (ODA) and Bill and Melinda Gates Foundation (BMGF) financial assistance not for health channelled via NGO's & civil society and private sector institutions | current US$ | continuous | - | - | Organisation for Economic Co-operation and Development (OECD) Creditor Reporting System (CRS) | https://stats.oecd.org/ | For all other categories - negative debt relief, administrative expenses and other in-donor country expenses |
| *Debt* |  |  |  |  |  |  |  |  |
| debt_stock_eppg | "External debt stocks, public and publicly guaranteed (PPG) (DOD, current US$)" | current US$ | continuous | "External debt stocks, public and publicly guaranteed (PPG) (DOD, current US$)" | DT.DOD.DPPG.CD | World Bank Databank: International Debt Statistics | https://databank.worldbank.org/source/international-debt-statistics |  |
| debt_serv_eppg | "Debt service on external debt, public and publicly guaranteed (PPG) (TDS, current US$)" | current US$ | continuous | "Debt service on external debt, public and publicly guaranteed (PPG) (TDS, current US$)" | DT.TDS.DPPG.CD | World Bank Databank: International Debt Statistics | https://databank.worldbank.org/source/international-debt-statistics |  |
| *IMF conditionalities* |  |  |  |  |  |  |  |  |
| imf_cond_ba1tot | "The main burden of adjustment indicator (BA1) measures the total number of conditions applicable in a given policy area for a given year in each country." (Total is for all policy areas combined. Includes both binding and non-binding conditions) | number | integer | Burden of Adjustment Indicator 1 | - | IMF Monitor | https://imfmonitor.org/conditionality/ |  |
| imf_cond_ba2tot | "An alternative burden of adjustment indicator (BA2) considers only binding conditions. The IMF attaches greater importance to their implementation and requires waivers if they are missed in order for loan disbursement to occur, thereby carrying greater coercive power than non-binding conditions." (Total is for all policy areas combined). | number | integer | Burden of Adjustment Indicator 2 | - | IMF Monitor | https://imfmonitor.org/conditionality/ |  |
| imf_cond_ba3tot | "Another burden of adjustment indicator (BA3) reflects the greater coercive power of binding conditions while also incorporating non-binding conditions. In this indicator, conditions are assigned a weighting according to the importance that the IMF attaches to their implementation. Binding conditions are assigned a weight of 2, while non-binding conditions are assigned a weight of 1." (Total is for all policy areas combined). | number | integer | Burden of Adjustment Indicator 3 | - | IMF Monitor | https://imfmonitor.org/conditionality/ |  |
| *Covariates* |  |  |  |  |  |  |  |  |
| pop | "Total population is based on the de facto definition of population, which counts all residents regardless of legal status or citizenship. The values shown are midyear estimates." | number | continuous | "Population, total" | SP.POP.TOTL | World Bank Databank: World Development Indicators | <https://databank.worldbank.org/data/source/world-development-indicators/preview/on> |  |
| gdp | "GDP at purchaser's prices is the sum of gross value added by all resident producers in the economy plus any product taxes and minus any subsidies not included in the value of the products. It is calculated without making deductions for depreciation of fabricated assets or for depletion and degradation of natural resources. Data are in current U.S. dollars. Dollar figures for GDP are converted from domestic currencies using single year official exchange rates. For a few countries where the official exchange rate does not reflect the rate effectively applied to actual foreign exchange transactions, an alternative conversion factor is used." | current US$ | continuous | "GDP (current US$)" | NY.GDP.MKTP.CD | World Bank Databank: World Development Indicators | <https://databank.worldbank.org/data/source/world-development-indicators/preview/on> |  |
| gdp_cap | "GDP per capita is gross domestic product divided by midyear population. GDP is the sum of gross value added by all resident producers in the economy plus any product taxes and minus any subsidies not included in the value of the products. It is calculated without making deductions for depreciation of fabricated assets or for depletion and degradation of natural resources. Data are in current U.S. dollars." | current US$ | continuous | "GDP per capita (current US$)" | NY.GDP.PCAP.CD | World Bank Databank: World Development Indicators | <https://databank.worldbank.org/data/source/world-development-indicators/preview/on> |  |
| mmr_me | "Maternal mortality ratio is the number of women who die from pregnancy-related causes while pregnant or within 42 days of pregnancy termination per 100,000 live births. The data are estimated with a regression model using information on the proportion of maternal deaths among non-AIDS deaths in women ages 15-49, fertility, birth attendants, and GDP measured using purchasing power parities (PPPs)." | # per 100,000 live births | continuous | "Maternal mortality ratio (modelled estimate, per 100,000 live births)" | SH.STA.MMRT | WHO, UNICEF, UNFPA, World Bank Group, and UNDESA/Population Division. Trends in Maternal Mortality 2000 to 2020. Geneva, World Health Organization, 2023 | <https://www.who.int/publications/i/item/9789240068759> |  |
| imr | "Infant mortality rate is the number of infants dying before reaching one year of age, per 1,000 live births in a given year." | # per 1000 live births | continuous | Mortality rate, infant (per 1,000 live births) | SP.DYN.IMRT.IN | Estimates developed by the UN Inter-agency Group for Child Mortality Estimation ( UNICEF, WHO, World Bank, UN DESA Population Division ) | [https://childmortality.org](https://childmortality.org/) |  |
| u5mr | "Under-five mortality rate is the probability per 1,000 that a newborn baby will die before reaching age five, if subject to age-specific mortality rates of the specified year." | # per 1000 live births | continuous | Mortality rate, under-5 (per 1,000 live births) | SH.DYN.MORT | Estimates developed by the UN Inter-agency Group for Child Mortality Estimation ( UNICEF, WHO, World Bank, UN DESA Population Division ) | [https://childmortality.org](https://childmortality.org/) |  |
| gov_eff | "Government Effectiveness captures perceptions of the quality of public services, the quality of the civil service and the degree of its independence from political pressures, the quality of policy formulation and implementation, and the credibility of the government's commitment to such policies. Estimate gives the country's score on the aggregate indicator, in units of a standard normal distribution, i.e. ranging from approximately -2.5 to 2.5." | Number (standard deviations) | continuous | "Government Effectiveness: Estimate" | GE.EST | Worldwide Governance Indicators (WGI). Source: "Daniel Kaufmann and Aart Kraay (2023). Worldwide Governance Indicators, 2023 Update" | https://www.govindicators.org/ |  |
| corr_control | "Control of Corruption captures perceptions of the extent to which public power is exercised for private gain, including both petty and grand forms of corruption, as well as "capture" of the state by elites and private interests. Estimate gives the country's score on the aggregate indicator, in units of a standard normal distribution, i.e. ranging from approximately -2.5 to 2.5." | Number (standard deviations) | continuous | "Control of Corruption: Estimate" | CC.EST | Worldwide Governance Indicators (WGI). Source: "Daniel Kaufmann and Aart Kraay (2023). Worldwide Governance Indicators, 2023 Update" | https://www.govindicators.org/ |  |
| battledeaths_orig | "Battle-related deaths are deaths in battle-related conflicts between warring parties in the conflict dyad (two conflict units that are parties to a conflict). Typically, battle-related deaths occur in warfare involving the armed forces of the warring parties. This includes traditional battlefield fighting, guerrilla activities, and all kinds of bombardments of military units, cities, and villages, etc. The targets are usually the military itself and its installations or state institutions and state representatives, but there is often substantial collateral damage in the form of civilians being killed in crossfire, in indiscriminate bombings, etc. All deaths--military as well as civilian--incurred in such situations, are counted as battle-related deaths." | Number | Integer | "Battle-related deaths (number of people)" | VC.BTL.DETH | Uppsala Conflict Data Program (UCDP) / Peace Research Institute Oslo (PRIO) Battle-Related Deaths Dataset version 23.1: - Davies, Shawn, Therese Pettersson & Magnus Öberg (2023). Organized violence 1989-2022 and the return of conflicts between states?. Journal of Peace Research 60(4). | https://ucdp.uu.se/downloads/ |  |
| battledeaths_imp | battledeaths_orig with 0's imputed for country-years without data, as the Battle-Related Deaths dataset only includes country-years with deaths | Number | Integer | - | - | Uppsala Conflict Data Program (UCDP) / Peace Research Institute Oslo (PRIO) Battle-Related Deaths Dataset version 23.1: - Davies, Shawn, Therese Pettersson & Magnus Öberg (2023). Organized violence 1989-2022 and the return of conflicts between states?. Journal of Peace Research 60(4). | https://ucdp.uu.se/downloads/ | Generated variable |
| battle_dum | 1 if number of battle-related deaths is higher than 1 per 1 million population; 0 if less (generated from battledeaths and pop-variables) | Number | Binary | - | - | Uppsala Conflict Data Program (UCDP) / Peace Research Institute Oslo (PRIO) Battle-Related Deaths Dataset version 23.1: - Davies, Shawn, Therese Pettersson & Magnus Öberg (2023). Organized violence 1989-2022 and the return of conflicts between states?. Journal of Peace Research 60(4). | https://ucdp.uu.se/downloads/ | Generated variable |
| col_indfrom | "The COW country code for the state, empire, or other entity from which this state gained independence. This variable is coded as missing where the state did not gain independence from a COW system member (e.g., by unifying local units into a new state)." | Correlates Of War (COW)-country code | Numerical | IndFrom | - | Paul R. Hensel (2018). "ICOW Colonial History Data Set, version 1.1." | <http://www.paulhensel.org/icowcol.html> |  |
| col_indfrom_uk | Independence was gained from United Kingdom. 1= true, 0= false. | Number | Binary | - |  | Paul R. Hensel (2018). "ICOW Colonial History Data Set, version 1.1." | <http://www.paulhensel.org/icowcol.html> | Generated variable |
| col_indfrom_fra | Independence was gained from France. 1= true, 0= false. | Number | Binary | - |  | Paul R. Hensel (2018). "ICOW Colonial History Data Set, version 1.1." | <http://www.paulhensel.org/icowcol.html> | Generated variable |
| col_indfrom_spa | Independence was gained from Spain. 1= true, 0= false. | Number | Binary | - |  | Paul R. Hensel (2018). "ICOW Colonial History Data Set, version 1.1." | <http://www.paulhensel.org/icowcol.html> | Generated variable |
| cpi | "Consumer price index reflects changes in the cost to the average consumer of acquiring a basket of goods and services that may be fixed or changed at specified intervals, such as yearly. The Laspeyres formula is generally used. Data are period averages." 2010 = 100 | Number | Continuous | "Consumer price index (2010 = 100)" | FP.CPI.TOTL | International Monetary Fund (IMF) | https://data.imf.org/?sk=4ffb52b2-3653-409a-b471-d47b46d904b5 |  |
| cpi_us_2020 | Same as above with base-year recalculated to 2020. | Number | Continuous | "Consumer price index” (2020 = 100) | - | International Monetary Fund (IMF) | https://data.imf.org/?sk=4ffb52b2-3653-409a-b471-d47b46d904b5 | Generated variable |
| gdpdefl_us_2020 | “The GDP implicit deflator is the ratio of GDP in current local currency to GDP in constant local currency. The base year varies by country.” 2020 = 100 | Number | Continuous | GDP deflator | NY.GDP.DEFL.ZS | World Bank | https://databank.worldbank.org/metadataglossary/world-development-indicators/series/NY.GDP.DEFL.ZS | Generated variable (recalculated from base year 2015) |
| cur_acc_bal_gdp | “Current account balance is the sum of net exports of goods and services, net primary income, and net secondary income.” | % | continuous | Current account balance (% of GDP) | BN.CAB.XOKA.GD.ZS | International Monetary Fund, Balance of Payments Statistics Yearbook and data files, and World Bank and OECD GDP estimates. | <https://data.imf.org/?sk=4c514d48-b6ba-49ed-8ab9-52b0c1a0179b&sid=1390030341854>  https://data.worldbank.org/indicator/BN.CAB.XOKA.GD.ZS |  |
| res_imp | “Total reserves comprise holdings of monetary gold, special drawing rights, reserves of IMF members held by the IMF, and holdings of foreign exchange under the control of monetary authorities. The gold component of these reserves is valued at year-end (December 31) London prices. This item shows reserves expressed in terms of the number of months of imports of goods and services they could pay for [Reserves/(Imports/12)].” | months | continuous | Total reserves in months of imports | FI.RES.TOTL.MO | International Monetary Fund, International Financial Statistics and data files. | <https://data.imf.org/?sk=4c514d48-b6ba-49ed-8ab9-52b0c1a0179b&sid=1390030341854>  https://data.worldbank.org/indicator/FI.RES.TOTL.MO |  |
| excrate | “Official exchange rate refers to the exchange rate determined by national authorities or to the rate determined in the legally sanctioned exchange market. It is calculated as an annual average based on monthly averages (local currency units relative to the U.S. dollar).” | Local currency units per US$ | continuous | Official exchange rate (LCU per US$, period average) | PA.NUS.FCRF | International Monetary Fund, International Financial Statistics. | <https://data.imf.org/?sk=4c514d48-b6ba-49ed-8ab9-52b0c1a0179b&sid=1390030341854>  https://data.worldbank.org/indicator/FI.RES.TOTL.MO |  |
| gdp_growth | “Annual percentage growth rate of GDP at market prices based on constant local currency. Aggregates are based on constant 2015 prices, expressed in U.S. dollars. GDP is the sum of gross value added by all resident producers in the economy plus any product taxes and minus any subsidies not included in the value of the products. It is calculated without making deductions for depreciation of fabricated assets or for depletion and degradation of natural resources.” | % | Continuous | GDP growth (annual %) | NY.GDP.MKTP.KD.ZG | World Bank national accounts data. | <https://data.worldbank.org/indicator/NY.GDP.MKTP.KD.ZG> |  |
| voice_acc | “Voice and Accountability captures perceptions of the extent to which a country's citizens are able to participate in selecting their government, as well as freedom of expression, freedom of association, and a free media. Estimate gives the country's score on the aggregate indicator, in units of a standard normal distribution, i.e. ranging from approximately -2.5 to 2.5.” | Number (standard deviations) | Continuous | Voice and Accountability: Estimate | VA.EST | Worldwide Governance Indicators (WGI). Source: "Daniel Kaufmann and Aart Kraay (2023). Worldwide Governance Indicators, 2023 Update" | https://www.govindicators.org/ |  |

# References

1.	Arregoces L, Daly F, Pitt C, Hsu J, Martinez-Alvarez M, Greco G, et al. Countdown to 2015: changes in official development assistance to reproductive, maternal, newborn, and child health, and assessment of progress between 2003 and 2012. The Lancet Global Health. 2015;3(7):e410-e21.

2.	OECD. Creditor Reporting System (CRS) 2024 [Available from: <https://stats.oecd.org/Index.aspx?DataSetCode=CRS1>. Last accessed 2023 February 16].

3.	Van de Sijpe N. Is Foreign Aid Fungible? Evidence from the Education and Health Sectors. World Bank Economic Review. 2013;27(2):320-56.

4.	Van de Sijpe N. The fungibility of health aid reconsidered. 2013.

5.	Lu C, Schneider MT, Gubbins P, Leach-Kemon K, Jamison D, Murray CJ. Public financing of health in developing countries: a cross-national systematic analysis. The Lancet. 2010;375(9723):1375-87.

6.	Dieleman JL, Graves CM, Hanlon M. The Fungibility of Health Aid: Reconsidering the Reconsidered. Journal of Development Studies. 2013;49(12):1755-62.

7.	Dieleman JL, Hanlon M. Measuring the displacement and replacement of government health expenditure. Health Econ. 2014;23(2):129-40.

8.	Patenaude BN. The relationship between development assistance for health and public health financing in 134 countries between 2000 and 2015. Health Policy Plan. 2021;36(4):369-83.

9.	Frimpong AO, Amporfu E, Arthur E. Effects of public and external health spending on out-of-pocket payments for healthcare in sub-Saharan Africa. Health Policy Plan. 2022;37(9):1129-37.

10.	WHO. Global Health Expenditure Database 2024 [Available from: <http://apps.who.int/nha/database>. Last accessed 2024 March 28].

11.	Feyzioglu T, Swaroop V, Zhu M. A Panel Data Analysis of the Fungibility of Foreign Aid. The World Bank Economic Review. 1998;12(1):29-58.

12.	Ali MEM, Ebaidalla M, Rizk R. Foreign aid and out-of-pocket health expenditure in Sub-Saharan Africa: does institutional development matter. International Journal of Sustainable Economy. 2020;12(3).

13.	World Bank. International Debt Statistics 2024 [Available from: [https://databank.worldbank.org/source/international-debt-statistics#](https://databank.worldbank.org/source/international-debt-statistics). Last accessed 2024 April 24].

14.	Lora E, Olivera M. Public debt and social expenditure: Friends or foes? Emerging Markets Review. 2007;8(4):299-310.

15.	IMF Monitor. IMF Conditionality Dataset, 1980-2019 2023 [Available from: <https://imfmonitor.org/conditionality/>. Last accessed 2023 November 2].

16.	Stubbs T, Kentikelenis A, Stuckler D, McKee M, King L. The impact of IMF conditionality on government health expenditure: A cross-national analysis of 16 West African nations. Social science & medicine (1982). 2017;174:220-7.

17.	Ooms G, Schrecker T. Expenditure ceilings, multilateral financial institutions, and the health of poor populations. The Lancet. 2005;365(9473):1821-3.

18.	Center for Global Development. Does the IMF Constrain Health Spending in Poor Countries? 2007 [Available from: <https://www.cgdev.org/sites/default/files/14103_file_IMF_report.pdf>. Last accessed 2024 March 19].

19.	Brunswijck G. Unhealthy Conditions. Eurodad; 2018.

20.	Stuckler D, Basu S. The International Monetary Fund's effects on global health: before and after the 2008 financial crisis. Int J Health Serv. 2009;39(4):771-81.

21.	Stuckler D, Basu S. The Body Economic: Why Austerity Kills. Basic Books; 2013. 978-0465063987.

22.	Abocejo F. The Impact of International Monetary Fund (IMF) Structural Adjustment Policies (SAP) on the Philippines. Recoletos Multidisciplinary Research Journal. 2014;2(1):19-24.

23.	Isiani MC, Anthonia Obi-Ani N, Obi-Ani P, Chidume CG, Okoye-Ugwu S. Interrogating the International Monetary Fund (IMF) Policies in Nigeria, 1986–2018. Cogent Arts & Humanities. 2021;8(1).

24.	Kingston K. The Impacts of the World Bank and IMF Structural Adjustment Programmes on Africa: The Case Study of Cote D'Ivoire, Senegal, Uganda, and Zimbabwe. Sacha Journal of Policy and Strategic Studies, Vol 1, No 2, pp 110-130, 2011. 2011.

25.	Foley EE. Your pocket is what cures you: The Politics of health in Senegal. Rutgers University Press; 2010. 978-0-8135-4667-4.

26.	Meurs M, Seidelmann L, Koutsoumpa M. How healthy is a 'healthy economy'? Incompatibility between current pathways towards SDG3 and SDG8. Global Health. 2019;15(1):83.

27.	Birn AE, Nervi L, Siqueira E. Neoliberalism Redux: The Global Health Policy Agenda and the Politics of Cooptation in Latin America and Beyond. Development and Change. 2016;47(4):734-59.

28.	Clements B, Gupta S, Nozaki M. What happens to social spending in IMF-supported programmes? Applied Economics. 2013;45(28):4022-33.

29.	Gupta S. Can a causal link be drawn? A commentary on "the impact of IMF conditionality on government health expenditure: A cross-national analysis of 16 West African nations". Social science & medicine (1982). 2017;181:199-201.

30.	Martin R, Segura-Ubiergo A. Social Spending in IMF-Supported Programs. International Monetary Fund; 2004.

31.	van der Gaag J, Barham T. Health and health expenditures in adjusting and non-adjusting countries. Social Science & Medicine. 1998;46(8):995-1009.

32.	Thomas AH. Do Debt-Service Savings and Grants Boost Social Expenditures? : International Monetary Fund; 2006.

33.	Forster T, Kentikelenis AE, Stubbs TH, King LP. Globalization and health equity: The impact of structural adjustment programs on developing countries. Social science & medicine (1982). 2020;267:112496.

34.	Kentikelenis A, Stubbs T. A Thousand Cuts: Social Protection in the Age of Austerity. editors: Kentikelenis A, Stubbs T. Oxford University Press; 2023. 0 p. 9780190637736.

35.	Stubbs TH, Kentikelenis AE, King LP. Catalyzing Aid? The IMF and Donor Behavior in Aid Allocation. World Development. 2016;78:511-28.

36.	World Bank. World Bank Open Data 2025 [Available from: <https://data.worldbank.org/>. Last accessed 2025 July 25].

37.	Lee SA, Lim J-Y. Does International Health Aid Follow Recipients’ Needs? Extensive and Intensive Margins of Health Aid Allocation. World Development. 2014;64:104-20.

38.	Reidpath DD, Allotey P. Infant mortality rate as an indicator of population health. J Epidemiol Community Health. 2003;57(5):344-6.

39.	UN Inter-agency Group for Child Mortality Estimation. Child Mortality, Stillbirth, and Causes of Death Estimates 2025 [Available from: <https://childmortality.org/>. Last accessed July 25 2025].

40.	Kaufmann D, Kraay A. Worldwide Governance Indicators: World Bank; 2023 [Available from: <https://www.govindicators.org/>. Last accessed 2024 March 21].

41.	Oryema JB, Gyimah-Brempong K, Picone G. The impact of debt relief on under five mortality rate in Sub-Saharan Africa. Glob Public Health. 2017;12(6):795-807.

42.	In’airat M. Aid allocation, selectivity, and the quality of governance. Journal of Economics Finance and Administrative Science. 2014;19(36):63-8.

43.	Sterck O, Roser M, Ncube M, Thewissen S. Allocation of development assistance for health: is the predominance of national income justified? Health Policy Plan. 2018;33(suppl_1):i14-i23.

44.	Rahim M. Quazi. Effects of Foreign Aid on GDP Growh and Fiscal Behavior: An Econometric Case Study of Bangladesh. The Journal of Developing Areas. 2005;38:95-117.

45.	Craig Burnside, David Dollar. Aid, Policies, and Growth: Revisiting the Evidence. World Bank Policy Research Working Paper 3251. 2004.

46.	Jung Y, Kim J, Kim K. Whom is economic aid meant for? The push vs. pull determinant factors of official development assistance. International Review of Economics & Finance. 2024;89:173-95.

47.	Nanda VP. The “Good Governance” Concept Revisited. The ANNALS of the American Academy of Political and Social Science. 2016;603(1):269-83.

48.	IMF. IMF and Good Governance 2024 [Available from: <https://www.imf.org/en/About/Factsheets/Sheets/2023/The-IMF-and-Good-Governance>. Last accessed 2024 February 9].

49.	Jajkowicz O, Drobiszová A. The Effect of Corruption on Government Expenditure Allocation in OECD Countries. Acta Universitatis Agriculturae et Silviculturae Mendelianae Brunensis. 2015;63(4):1251-9.

50.	Hashem EA. The Effects of Corruption on Government Expenditures: Arab

Countries Experience. Journal of Economics and Sustainable Development. 2014;5(4).

51.	Davies S, Pettersson T, Öberg M. Organized violence 1989–2022, and the return of conflict between states. Journal of Peace Research. 2023;60(4):691-708.

52.	World Bank. World Bank Group Assistance to Low-Income Fragile and Conflict-Affected States: An Indepedent Evaluation. 2014:166.

53.	Yogo UT, Mallaye D. Health Aid and Health Improvement in Sub Saharan Africa. Munich Personal RePEc Archive. 2012(44938).

54.	Collier P, Hoeffler A. Aid, Policy, and Growth in Post-Conflict Societies. World Bank Policy Research Working Paper 2902. October 2002.

55.	Bluhm R, Gassebner M, Langlotz S, Schaudt P. Fueling Conflict? (De)Escalation and Bilateral Aid. AidData Working Paper. 2016;31.

56.	Mishra P, Newhouse D. Does health aid matter? J Health Econ. 2009;28(4):855-72.

57.	Dietrich S. The Politics of Public Health Aid: Why Corrupt Governments Have Incentives to Implement Aid Effectively. World Development. 2011;39(1):55-63.

58.	Alesina A, Dollar D. Who Gives Foreign Aid to Whom and Why? Journal of Economic Growth. 2000;5(1):33-63.

59.	Matteis AD. Whose poverty really matters when deciding aid volumes? International Journal of Public Policy. 2016;12(1/2).

60.	Neumayer E. The Determinants of Aid Allocation by Regional Multilateral Development Banks and United Nations Agencies. International Studies Quarterly. 2003;47(1):101-22.

61.	Jones P. History matters: New evidence on the long run impact of colonial rule on institutions. Journal of Comparative Economics. 2013;41(1):181-200.

62.	Robin M. Grier. Colonial Legacies and Economic Growth. Public Choice. 1999;98(3/4):317-35.

63.	Acemoglu D, Robinson JA, Johnson S. The Colonial Origins of Comparative Development:

An Empirical Investigation. American Economic Review 2001;91(5):1369-401.

64.	Azevedo MJ. The State of Health System(s) in Africa: Challenges and Opportunities. In: Azevedo MJ, editor. Historical Perspectives on the State of Health and Health Systems in Africa, Volume II: The Modern Era. Cham: Springer International Publishing; 2017. p. 1-73.

65.	Hensel PR. ICOW Colonial History Data Set, version 1.1 2023 [Available from: <http://www.paulhensel.org/icowcol.html>. Last accessed 2023 November 17].

66.	Kentikelenis AE, Stubbs TH, King LP. Structural adjustment and public spending on health: evidence from IMF programs in low-income countries. Social science & medicine (1982). 2015;126:169-76.

67.	Clements B, Gupta S, Nozaki M. What Happens to Social Spending in IMF-Supported Programs? 2011 [Available from: <http://www.imf.org/external/pubs/ft/sdn/2011/sdn1115.pdf>.

68.	Nooruddin I, Simmons JW. The Politics of Hard Choices: IMF Programs and Government Spending. International Organization. 2006;60(04).

69.	Daoud A, Reinsberg B. Structural adjustment, state capacity and child health: evidence from IMF programmes. Int J Epidemiol. 2019;48(2):445-54.

70.	IMF. International Finance Statistics (IFS) 2024 [Available from: <https://data.imf.org/?sk=4c514d48-b6ba-49ed-8ab9-52b0c1a0179b&sid=1390030341854>. Last accessed 2024 March 21].

71.	Center for Systemic Peace. Polity5 dataset 2020 [Available from: <https://www.systemicpeace.org/polityproject.html>. Last accessed 2024 March 21].

1. We counted all BMGF development finance on the OECD CRS database as grants, even though this is not specified in the database. This was based on the conservative assumption that the vast majority if not all of these transfers are indeed grants, since the BMGF had given 34523 total grants with a substantial proportion to health, and only 10 loans within health as of November 10, 2023 (<https://www.gatesfoundation.org/about/committed-grants>; <https://sif.gatesfoundation.org/portfolio/>).
