# Supplementary material for: Aid, debt, International Monetary Fund conditionalities and domestic health financing in low- and middle-income countries
Source: Health Policy Plan. 2026 May 21;41(7):1049–66. doi: 10.1093/heapol/czag070 (PMC13481784; doi:10.1093/heapol/czag070)
Supplement: Supplementary file 1 [file czag070_supplementary_data.zip › Supplementary_Legends.docx]

Table 2

Title: Child Health Accountability Tracking Technical Advisory Group*^,^ ** recommended health and well-being indicators* for children ages 1 month to 9 years of age, definition, Nurturing Care Framework domain, and description of how the indicator may reflect climate change related effects^

Legend for Table 2:

All indicators to be disaggregated as possible by key population characteristics of relevance (i.e., wealth quintile, geographic location, maternal education, maternal age, and sex of the child) to promote equity.

*The Child Health Accountability Tracking technical advisory group is co-convened by the World Health Organization and the United Nations Children’s Fund. Its main objective is to support the harmonization and standardization of child health and well-being indicators across a wide range of domains including physical and mental health, education, nutrition, safety and security, and the environment. The focus is on children ages 1 month to 9 years of age https://www.who.int/groups/child-health-accountability-tracking-technical-advisory-group/about-us.

** More detailed information on how these indicators were selected, with a focus on the health sector and metadata, is available in: Requejo J, Strong K, Agweyu A, Billah SM, Boschi-Pinto C, Horiuchi S, Jamaluddine Z, Lazzerini M, Maiga A, McKerrow N, Munos M, Park L, Schellenberg J, Weigel R. Measuring and monitoring child health and wellbeing: recommendations for tracking progress with a core set of indicators in the Sustainable Development Goals era. *Lancet Child Adolesc Health*. 2022 May;6(5):345-352. doi: 10.1016/S2352-4642(22)00039-6. PMID: 35429452; PMCID: PMC9764429.

^Research is needed to develop a comprehensive measurement framework for monitoring climate change-related effects and adaptation, mitigation, and response efforts for child health and well-being. These indicators can be used as a starting point for monitoring efforts.

Figure 1

Title: Climate change impacts on child health and well-being, direct and indirect pathways

Legend for Figure 1:

Source: Hellden et al., 2021. Climate change and child health: a scoping review and an expanded conceptual framework. *Lancet Planet Health,* 5(e164-75). Figure 3: Climate change and child health: an expanded framework. The dashed lines show where mitigation and adaptation can hinder the effects of climate change on child health and wellbeing. HFMD= Hand, foot, and mouth disease. *Other infectious diseases, from mumps to bacterial meningitis, varicella viruses, and parasitic diseases.
